# Supplementary material for: Genome-wide assessment identifies novel runs of homozygosity linked to Parkinson’s disease etiology across diverse ancestral populations
Source: medRxiv. 2025 May 22:2025.05.21.25328076. Preprint. [Version 1] doi: 10.1101/2025.05.21.25328076 (PMC12140525; doi:10.1101/2025.05.21.25328076)
Supplement: Supplement 1 [file media-1.pdf]

Appendix

Genome-wide assessment identifies novel runs of homozygosity linked to Parkinson's disease etiology across diverse ancestral populations

Kathryn Step[#] and Carlos F. Hernández[#], Esraa Eltaraifee, Ana Jimena Hernández-Medrano, Pin-Jui Kung, Miriam Ostroževičová, Alexandra Zirra, Eduardo Pérez-Palma, Niccolò E. Mencacci, Ignacio J. Keller Sarmiento, Huw R. Morris, Ignacio F. Mata, Global Parkinson's Genetics Program (GP2), Juliana Acosta-Uribe, Zih-Hua Fang* and Sara Bandres-Ciga*

[#] These authors share first authorship; *these authors share last authorship

Table of contents

Supplementary Materials 1

ROH calculations 1

Supplementary Figures 1

Supplementary Figure 1: Scree plots illustrating eigenvalues for principal component analysis (PCA). The eigenvalues of each principal component indicate the proportion of variance explained by each component. The point at which the eigenvalues level off suggests the optimal number of components to retain as covariates in the analysis. AAC = African Admixed, AFR = African, AJ = Ashkenazi Jewish, AMR = American Admixed, CAS = Central Asian, EAS = East Asian, EUR = European, MDE = Middle-Eastern, SAS = South Asian 1

Supplementary Figure 2: Box plots illustrating the distribution of four ROH parameters assessed colored by phenotype for each ancestral group. The x-axis represents the ancestry and the y-axis represents the ROH parameter. AAC = African Admixed, AFR = African, AJ = Ashkenazi Jewish, AMR = American Admixed, ANC = ancestry, AV_{ROH} = average length of runs of homozygosity, CAS = Central Asian, EAS = East Asian, EUR = European, F_{ROH} = runs of homozygosity-based estimates for inbreeding coefficient, MDE = Middle-Eastern, N_{ROH} = number of runs of homozygosity, SAS = South Asian, S_{ROH} = total length of runs of homozygosity. 2

.... 2

Supplementary Figure 3: (A) Mean N_{ROH} versus S_{ROH} in Mb for each of the nine ancestral groups. (B) Mean N_{ROH} versus the S_{ROH} in Mb for each individual included in the analysis. AAC = African Admixed, AFR = African, AJ = Ashkenazi Jewish, AMR = American Admixed, CAS = Central Asian, EAS = East Asian, EUR = European, MDE = Middle-Eastern, ROH = runs of homozygosity, SAS = South Asian, S_{ROH} = total length of runs of homozygosity. 3

Supplementary Figure 4: Comparison of the runs of homozygosity versus length of runs of homozygosity for cases and controls across the ancestral groups. For phenotype, 1 indicates control and 2 indicates case. AAC = African Admixed, AFR = African, AJ = Ashkenazi Jewish, AMR = American Admixed, CAS = Central Asian, EAS = East Asian, EUR = European, KB = kilobase (S_{ROH}), MDE = Middle-Eastern, NSEG = number of runs of homozygosity (N_{ROH}), PHE = phenotype, SAS = South Asian 4

Supplementary Figure 5: Comparison of the runs of homozygosity versus average length of runs of homozygosity for cases and controls across the ancestral groups. For phenotype, 1 indicates control and 2 indicates case. AAC = African Admixed, AFR = African, AJ = Ashkenazi Jewish, AMR = American Admixed, CAS = Central Asian, EAS = East Asian, EUR = European, KBAVG = average number of runs of homozygosity (AV_{ROH}), MDE = Middle-Eastern, NSEG = number of runs of homozygosity (N_{ROH}), SAS = South Asian.. 5

Supplementary Figure 6: Density plot illustrating the distribution of inbreeding coefficients (F_{ROH}) across the ancestry groups for (A) cases and controls, (B) cases only, and (C) controls only. The x-axis represents the inbreeding coefficient values ranging from -0.5 to 0.25, while the y-axis represents the density of observations. AAC = African Admixed, AFR = African, AJ = Ashkenazi Jewish, AMR = American Admixed, CAS = Central Asian, EAS = East Asian, EUR = European, MDE = Middle-Eastern, SAS = South Asian...6

Supplementary Figure 7: Density plots illustrating the distribution of inbreeding coefficients (F_{ROH}) across the ancestral groups. The x-axis represents the F_{ROH} values, while the y-axis represents the density of observations at each F value. AAC = African Admixed, AFR = African, AJ = Ashkenazi Jewish, AMR =

American Admixed, CAS = Central Asian, EAS = East Asian, EUR = European, MDE = Middle-Eastern, SAS = South Asian....7

Supplementary Figure 8: Density plots illustrating the distribution of inbreeding coefficients (F_{ROH}) colored by phenotype for each ancestral group. For phenotype, 0 indicates control and 1 indicates case. Each curve represents the density of observations for a specific phenotype value. The x-axis represents the values of F, while the y-axis represents the density of observations at each F value. AAC = African Admixed, AFR = African, AJ = Ashkenazi Jewish, AMR = American Admixed, CAS = Central Asian, EAS = East Asian, EUR = European, MDE = Middle-Eastern, SAS = South Asian....8

Supplementary Figure 9: Box plots illustrating the distribution of four ROH parameters assessed colored by phenotype including early and late onset PD for each ancestral group. The y-axis represents the ancestral group. AAC = African Admixed, AFR = African, AJ = Ashkenazi Jewish, AMR = American Admixed, ANC = ancestry, CAS = Central Asian, EAS = East Asian, EUR = European, F_{ROH} = run of homozygosity based estimates for the inbreeding coefficient, KB = kilobases, KBAVG = average length of runs of homozygosity, MDE = Middle-Eastern, NSEG = number of runs of homozygosity, SAS = South Asian. 9

Supplementary Figure 10: Homozygosity mapping plots displaying runs of homozygosity enriched in cases across diverse ancestries. Highlighted in red are the runs of homozygosity present only in cases. The chromosome is along the x-axis and the runs of homozygosity size in base positions in along the y-axis. AAC = African Admixed, AFR = African, AJ = Ashkenazi Jewish, AMR = American Admixed, CAS = Central Asian, EAS = East Asian, EUR = European, MDE = Middle-Eastern, SAS = South Asian....10

Supplementary Figure 11: Heatmap illustrating the number of individuals or runs of homozygosity obtained from the homozygosity mapping analysis for individuals with consanguinity. AAC = African Admixed, AFR = African, AJ = Ashkenazi Jewish, AMR = American Admixed, CAS = Central Asian, EAS = East Asian, EUR = European, MDE = Middle-Eastern, SAS = South Asian. The Bonferroni threshold was adjusted by dividing 0.05 by the number of enriched ROHs to account for multiple comparisons. 11

Supplementary Figure 12: Heatmap illustrating the number of individuals or runs of homozygosity obtained from the homozygosity mapping analysis for early-onset Parkinson's disease patients. AAC = African Admixed, AFR = African, AJ = Ashkenazi Jewish, AMR = American Admixed, CAS = Central Asian, EAS = East Asian, EUR = European, MDE = Middle-Eastern, SAS = South Asian. The Bonferroni threshold was adjusted by dividing 0.05 by the number of enriched ROHs to account for multiple comparisons....12

Supplementary Tables....13

Supplementary Table 1: Study participant demographic information for NeuroBooster array genotyping imputed data....13

Supplementary Table 2: Study participant demographic information for whole-genome sequencing data 14

Supplementary Table 3: Parameters used for the genome-wide homozygosity analyses....15

Supplementary Table 4: Missing age information by Ancestry and the corresponding mean age for imputation....16

Supplementary Table 5: List of the known Parkinson's disease, pallido-pyramidal syndrome, and atypical parkinsonism gene regions and risk loci....16

Supplementary Table 6: Putative family structures identified in the Middle-Eastern group for runs of homozygosity segregating in families....27

Supplementary Table 7: T-test results for measures of genome-wide homozygosity in Parkinson's disease 28

Supplementary Table 8: Measures of genome-wide homozygosity in Parkinson's disease excluding Parkinson's disease recessive genes (PRKN, PINK1, and DJ-1) ... 29

Supplementary Table 9: Linear regression for runs of homozygosity associated with Parkinson's disease at age of onset ... 31

Supplementary Table 10: Runs of homozygosity associated with Parkinson's disease at age of onset excluding Parkinson's disease recessive genes (PRKN, PINK1, and DJ-1) ... 32

Supplementary Table 11: Runs of homozygosity in cases subsetted for different age of onset of Parkinson's disease versus controls ... 33

Supplementary Table 12: T-test results for runs of homozygosity in cases subsetted for early-onset Parkinson's disease and late-onset Parkinson's disease versus controls ... 37

Supplementary Table 13: Runs of homozygosity in cases subsetted for early-onset and late-onset Parkinson's disease versus controls excluding Parkinson's disease recessive genes (PRKN, PINK1, and DJ-1) ... 38

Supplementary Table 14: T-test results for runs of homozygosity in cases early-onset Parkinson's disease versus late-onset Parkinson's disease ... 41

Supplementary Table 15: Runs of homozygosity only in cases early-onset versus late-onset Parkinson's disease excluding Parkinson's disease recessive genes (PRKN, PINK1, and DJ-1) ... 43

Supplementary Table 16: Average runs of homozygosity (2Mb to 10Mb) across ancestry groups ... 44

Supplementary Table 17: Average runs of homozygosity (2Mb to 10Mb) for early-onset and late-onset Parkinson's disease across ancestry groups ... 45

Supplementary Table 18: Homozygosity mapping results for runs of homozygosity enriched in individuals with consanguinity and early-onset Parkinson's disease. ... 50

Supplementary Table 19: Annotated variants only present in Parkinson's disease cases prioritized from homozygosity mapping with additional annotation information ... 52

Supplementary Table 20: Runs of homozygosity pools enriched in individuals with consanguinity that pass Bonferroni correction ... 56

Supplementary Table 21: Runs of homozygosity pools enriched in individuals with consanguinity that pass Bonferroni correction intersecting with known Parkinson's disease, dystonia, and atypical parkinsonism gene regions and risk loci ... 148

Supplementary Table 22: Runs of homozygosity pools enriched in early-onset cases that pass Bonferroni correction ... 161

References ... 163

Banner Authors: Global Parkinson’s Genetics Program (GP2) ... 164

Supplementary Materials

ROH calculations

Sum of ROH (S_{ROH})

The mean length of ROH is calculated by dividing the sum of ROH lengths by number of ROH segments, as follows:

$$Mean\ ROH\ =\ \frac{Sum\ of\ ROH\ lengths}{Number\ of\ ROH\ segments}$$

$$SROH\ =\ \Sigma_{i=1}^{n}\ Length\ of\ ROH_i$$

Where n is the number of ROH segments and the length of ROH and $Length\ of\ ROH_i$ is the number of consecutive homozygous markers in a segment.

Average length of ROH (AVROH)

The AV_{ROH} provides the average measure of length of homozygous regions in an individual’s genome. This is calculated by dividing the sum of the lengths of all the individual ROH by the total number of ROH, as follows:

$$Average\ ROH\ =\ \frac{\Sigma_{i=1}^{n} Length\ of\ ROH_i}{n}$$

Where n is the total number of ROH segments and $Length\ of\ ROH_i$ is the length of the ith ROH.

Number of ROH (N_{ROH})

The N_{ROH} is the count of the total number of occurrences of ROH segments in an individual’s genome. Here each ROH segment is considered one ROH occurrence and the total number of these segments is the N_{ROH} in the genome, as follows:

$$NROH\ =\ Total\ number\ of\ ROH\ segments$$

ROH-based estimates of the inbreeding coefficient per individual (F_{ROH})

The inbreeding coefficient (F_{ROH}) indicates the degree of relatedness due to shared ancestry. It is the probability of an individual inheriting two alleles at a locus from a common ancestor without a recombination event. In a ROH context, F_{ROH} estimates F based on the number of length of ROH segments in an individual’s genome. It is the ratio of the sum of the lengths of ROH segments and the total length of the autosomal genome containing ROH segments. The F_{ROH} provides the proportion of the genome that is homozygous due to recent common ancestry and is calculated as follows:

$$FROH\ =\ \frac{\Sigma_{i=1}^{n} Length\ of\ ROH_i}{Lgenome}$$

Where n is the total number of ROH segments, $Length\ of\ ROH_i$ is the length of the ith ROH segment, and L_{genome} is the total length of the autosomal genome containing ROH segments.

Supplementary Figures

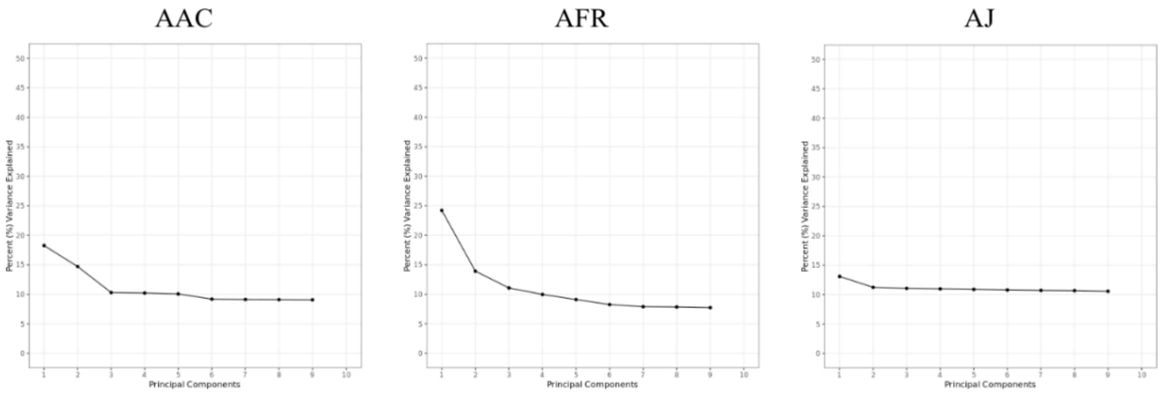

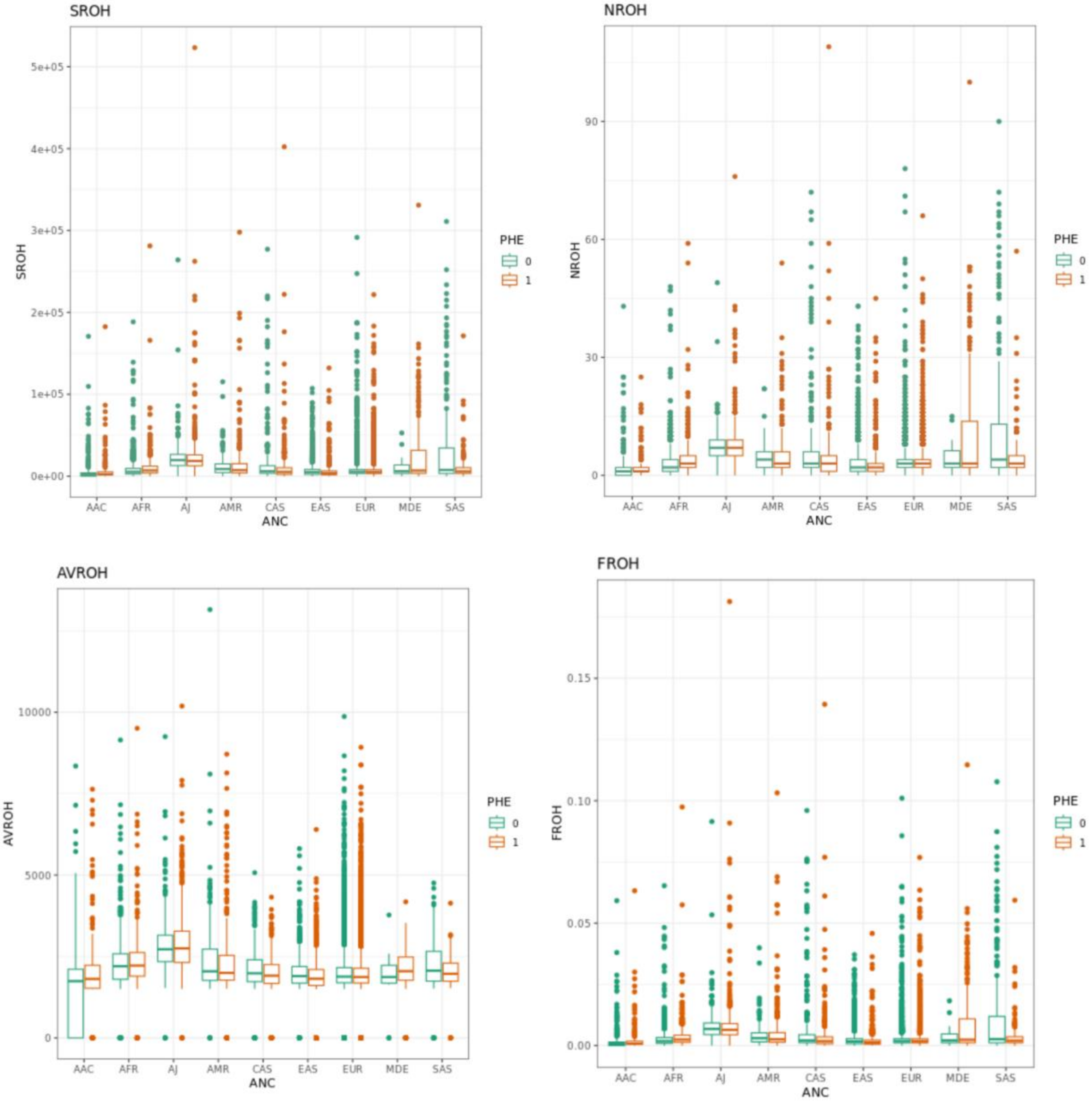

Supplementary Figure 2*: Box plots illustrating the distribution of four ROH parameters assessed colored by phenotype for each ancestral group. The x-axis represents the ancestry and the y-axis represents the ROH parameter. AAC = African Admixed, AFR = African, AJ = Ashkenazi Jewish, AMR = American Admixed, ANC = ancestry, AV_{ROH} = average length of runs of homozygosity, CAS = Central Asian, EAS = East Asian, EUR = European, F_{ROH} = runs of homozygosity-based estimates for inbreeding coefficient, MDE = Middle-Eastern, N_{ROH} = number of runs of homozygosity, SAS = South Asian, S_{ROH} = total length of runs of homozygosity.*

(A) Per ancestral group

(B) Per individual

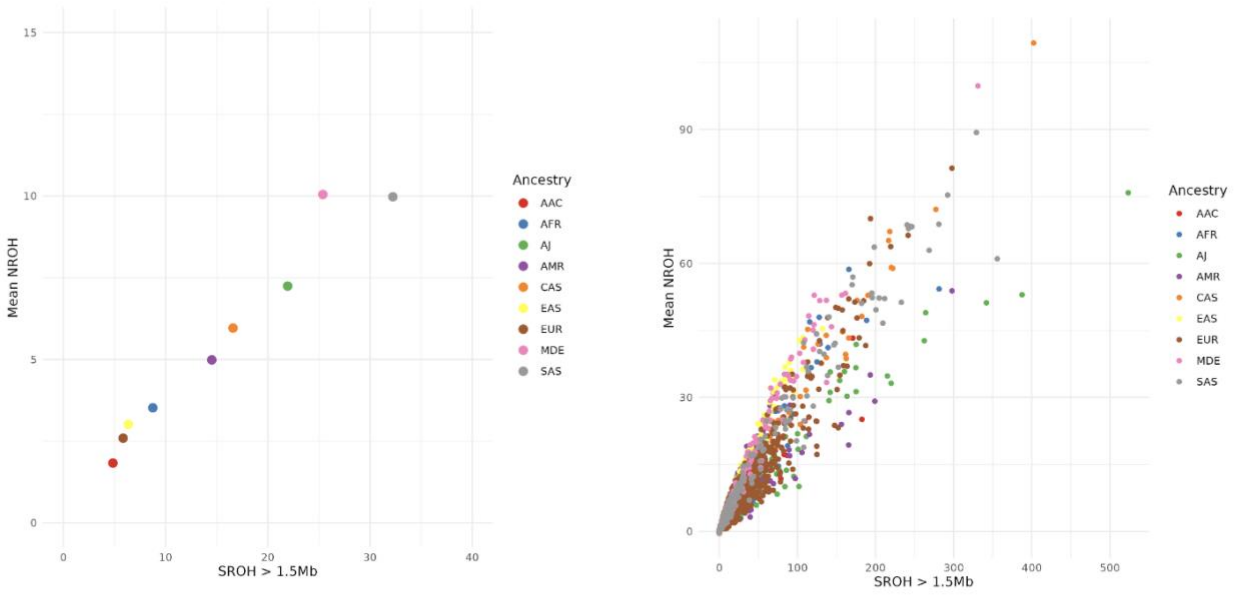

Supplementary Figure 3: *(A) Mean* N_{ROH} *versus* S_{ROH} *in Mb for each of the nine ancestral groups. (B) Mean* N_{ROH} *versus the* S_{ROH} *in Mb for each individual included in the analysis. AAC = African Admixed, AFR = African, AJ = Ashkenazi Jewish, AMR = American Admixed, CAS = Central Asian, EAS = East Asian, EUR = European, MDE = Middle-Eastern, ROH = runs of homozygosity, SAS = South Asian,* S_{ROH} *= total length of runs of homozygosity.*

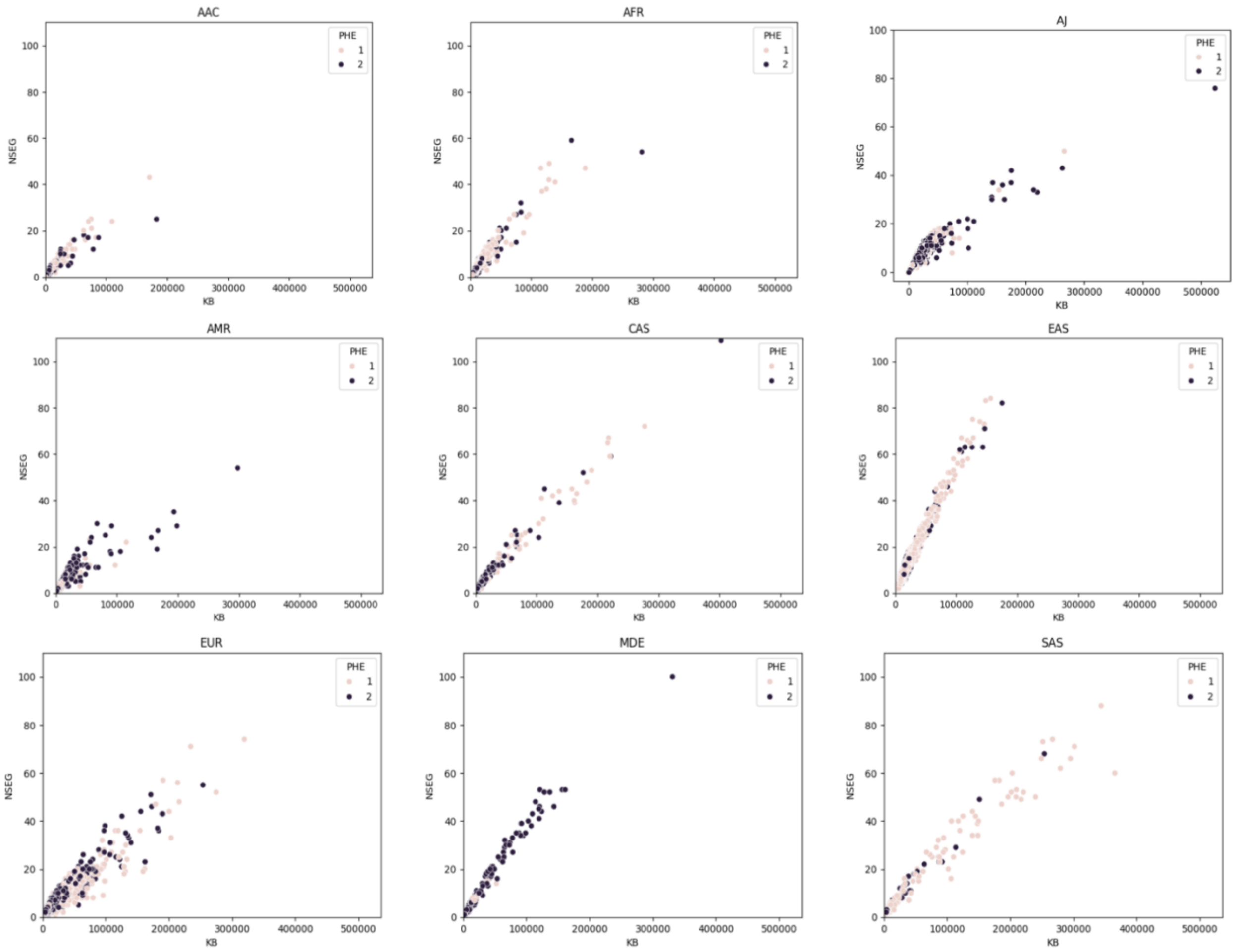

Supplementary Figure 4: *Comparison of the runs of homozygosity versus length of runs of homozygosity for cases and controls across the ancestral groups. For phenotype, 1 indicates control and 2 indicates case. AAC = African Admixed, AFR = African, AJ = Ashkenazi Jewish, AMR = American Admixed, CAS = Central Asian, EAS = East Asian, EUR = European, KB = kilobase (S_{ROH}), MDE = Middle-Eastern, NSEG = number of runs of homozygosity (N_{ROH}), PHE = phenotype, SAS = South Asian*

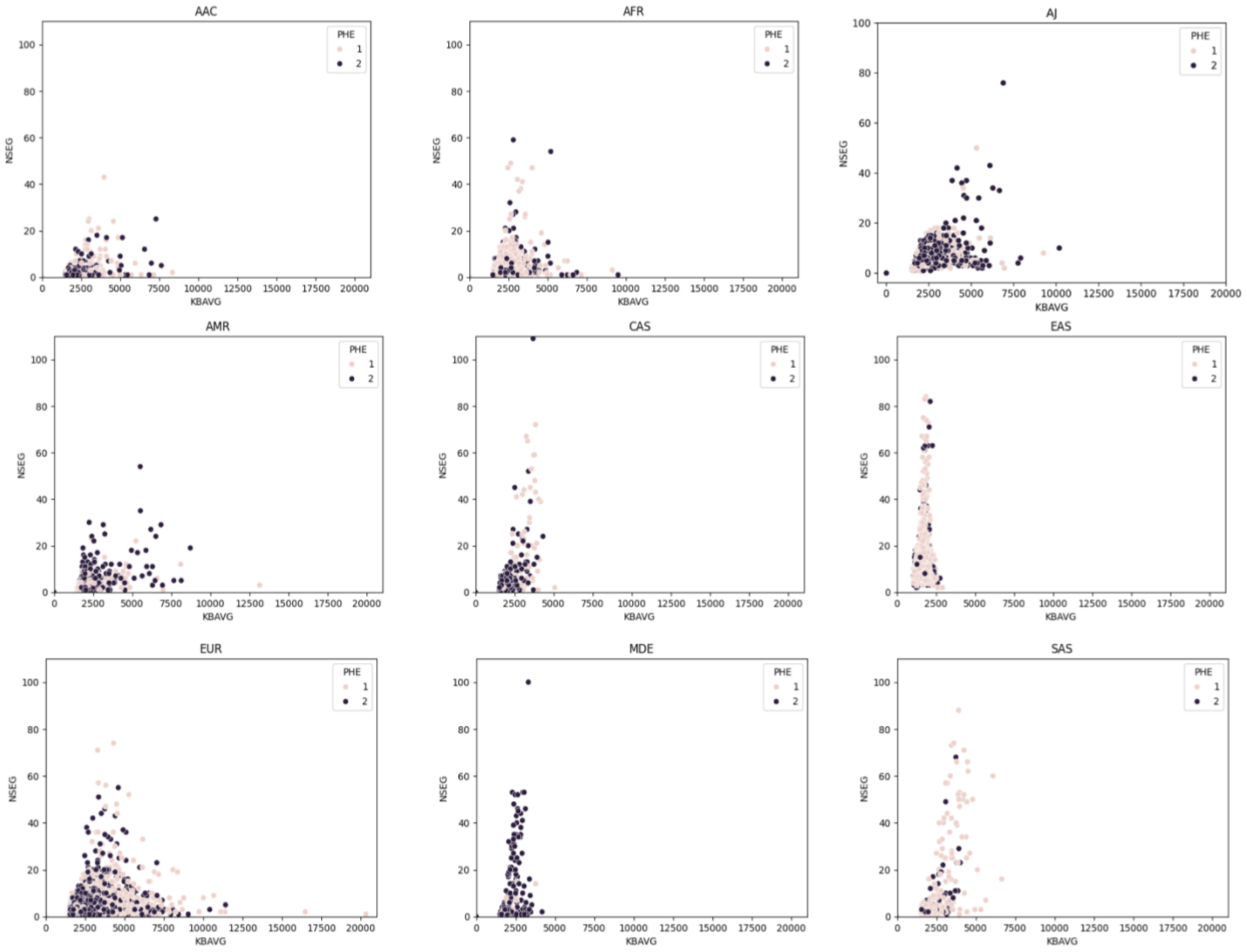

Supplementary Figure 5: *Comparison of the runs of homozygosity versus average length of runs of homozygosity for cases and controls across the ancestral groups. For phenotype, 1 indicates control and 2 indicates case. AAC = African Admixed, AFR = African, AJ = Ashkenazi Jewish, AMR = American Admixed, CAS = Central Asian, EAS = East Asian, EUR = European, KBAVG = average number of runs of homozygosity (*AV_{ROH}*), MDE = Middle-Eastern, NSEG = number of runs of homozygosity (*N_{ROH}*), SAS = South Asian*

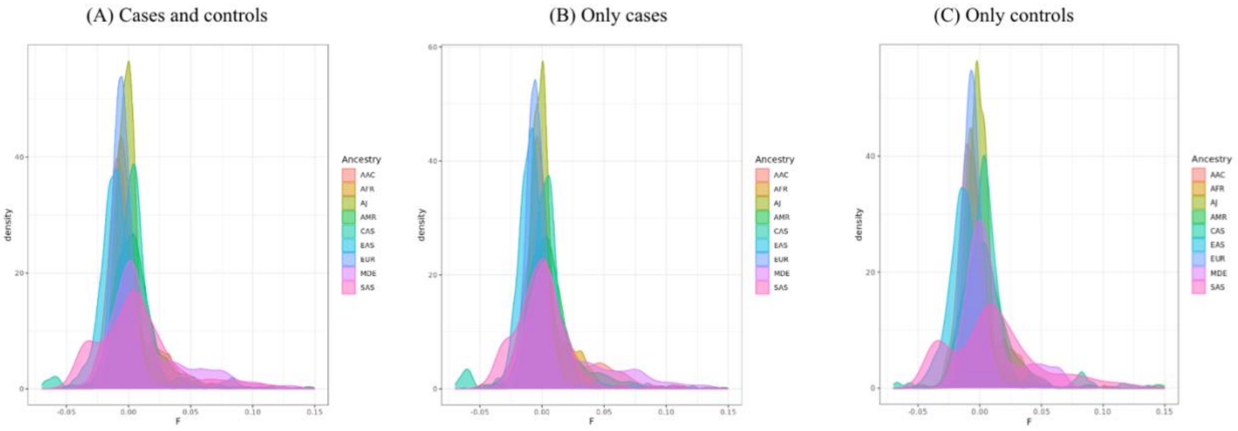

Supplementary Figure 6: *Density plot illustrating the distribution of inbreeding coefficients (F_{ROH}) across the ancestry groups for (A) cases and controls, (B) cases only, and (C) controls only. The x-axis represents the inbreeding coefficient values ranging from -0.5 to 0.25, while the y-axis represents the density of observations. AAC = African Admixed, AFR = African, AJ = Ashkenazi Jewish, AMR = American Admixed, CAS = Central Asian, EAS = East Asian, EUR = European, MDE = Middle-Eastern, SAS = South Asian.*

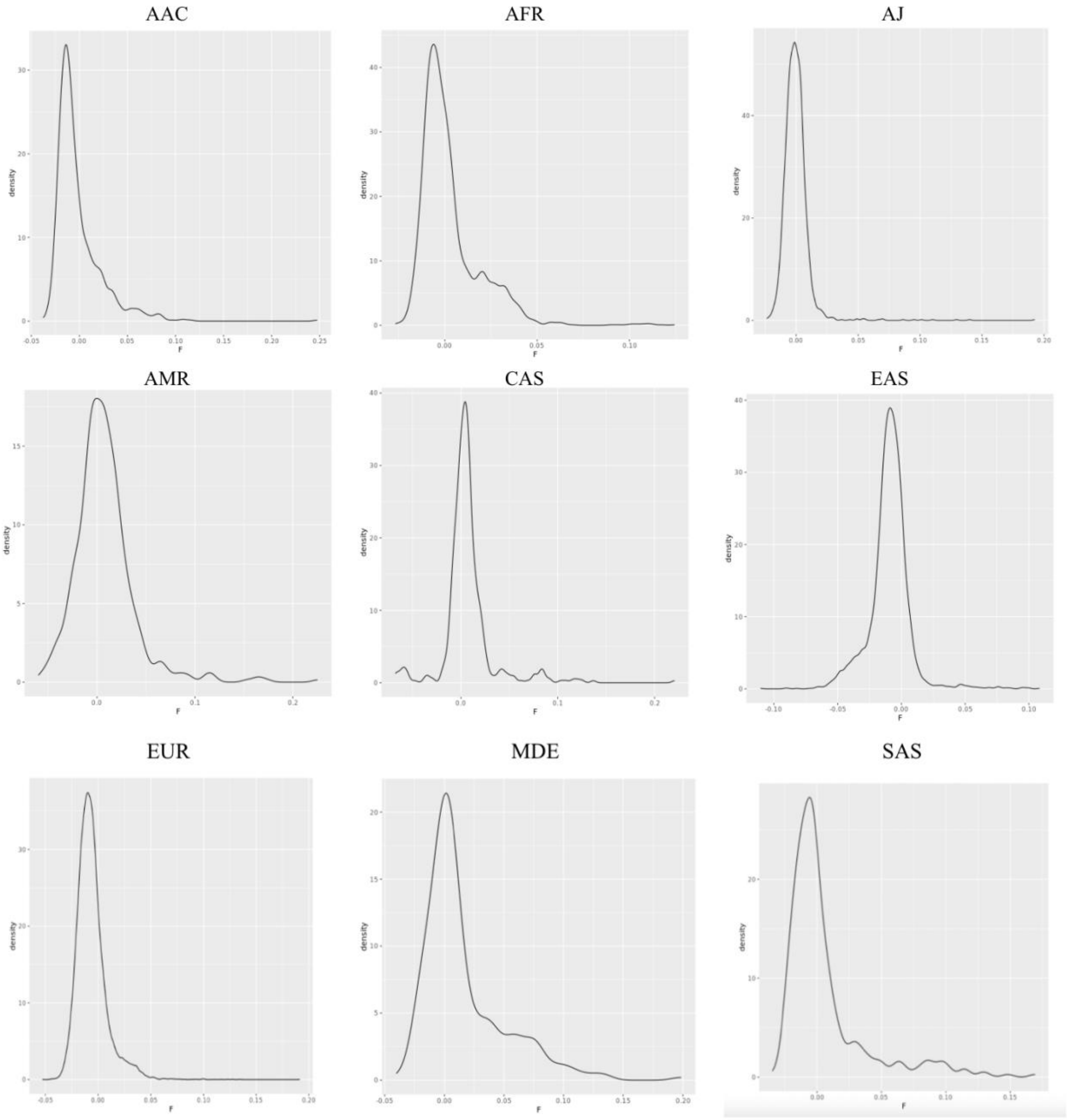

Supplementary Figure 7: *Density plots illustrating the distribution of inbreeding coefficients (F_{ROH}) across the ancestral groups. The x-axis represents the F_{ROH} values, while the y-axis represents the density of observations at each F value. AAC = African Admixed, AFR = African, AJ = Ashkenazi Jewish, AMR = American Admixed, CAS = Central Asian, EAS = East Asian, EUR = European, MDE = Middle-Eastern, SAS = South Asian*

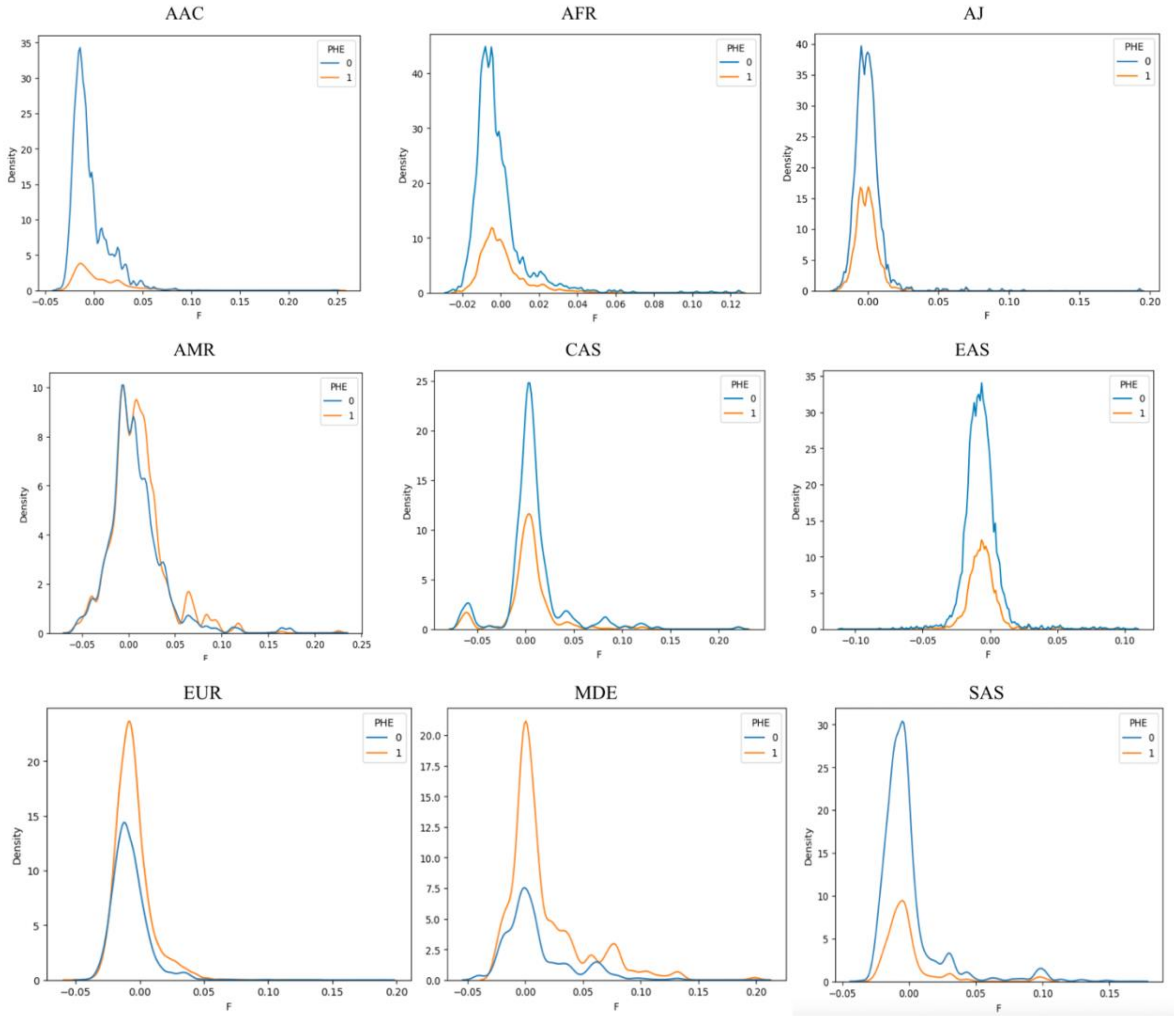

Supplementary Figure 8: *Density plots illustrating the distribution of inbreeding coefficients (F_{ROH}) colored by phenotype for each ancestral group. For phenotype, 0 indicates control and 1 indicates case. Each curve represents the density of observations for a specific phenotype value. The x-axis represents the values of F, while the y-axis represents the density of observations at each F value. AAC = African Admixed, AFR = African, AJ = Ashkenazi Jewish, AMR = American Admixed, CAS = Central Asian, EAS = East Asian, EUR = European, MDE = Middle-Eastern, SAS = South Asian.*

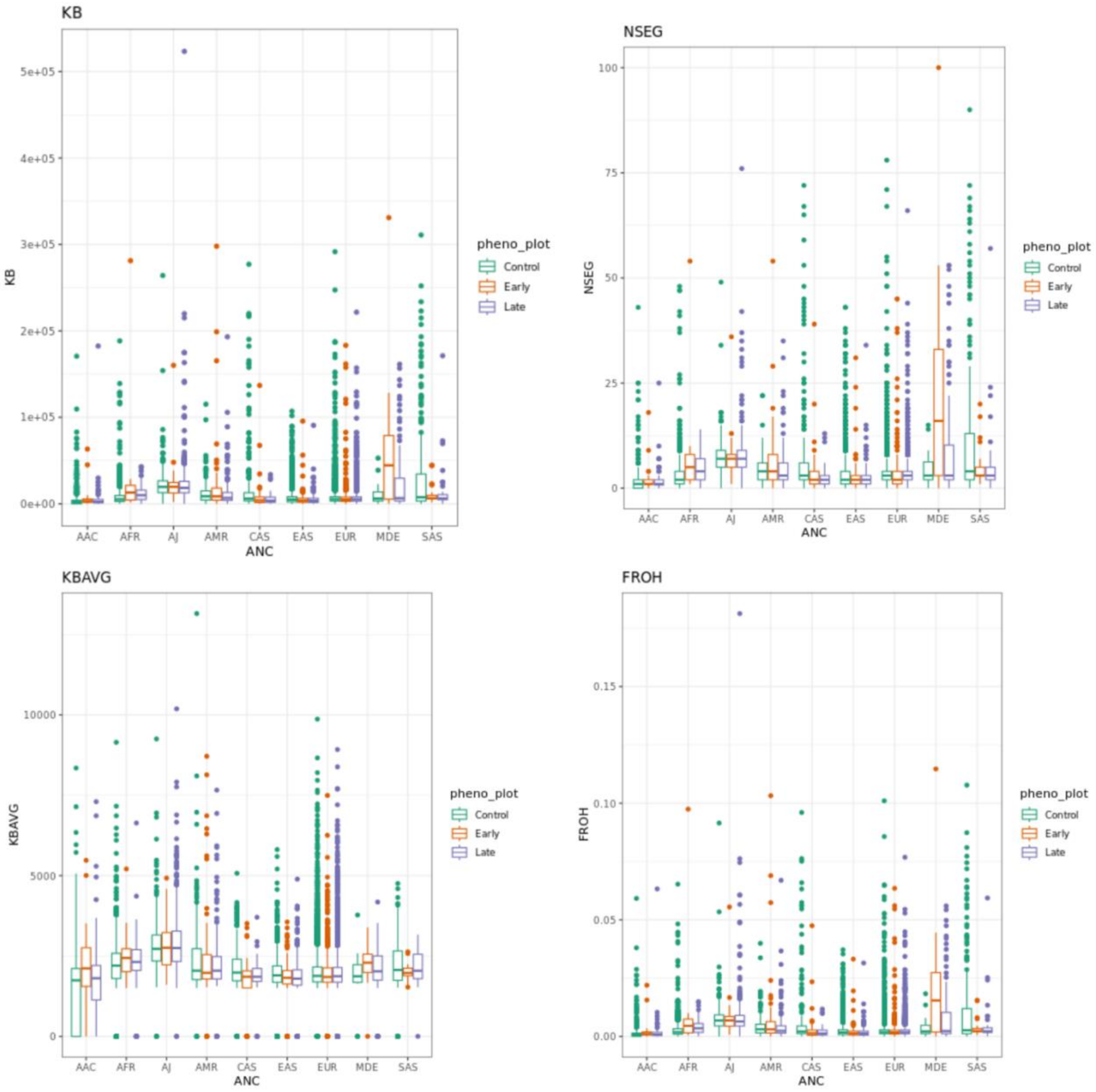

Supplementary Figure 9: *Box plots illustrating the distribution of four ROH parameters assessed colored by phenotype including early and late onset PD for each ancestral group. The y-axis represents the ancestral group. AAC = African Admixed, AFR = African, AJ = Ashkenazi Jewish, AMR = American Admixed, ANC = ancestry, CAS = Central Asian, EAS = East Asian, EUR = European,* F_{ROH} *= run of homozygosity based estimates for the inbreeding coefficient, KB = kilobases, KBAVG = average length of runs of homozygosity, MDE = Middle-Eastern, NSEG = number of runs of homozygosity, SAS = South Asian.*

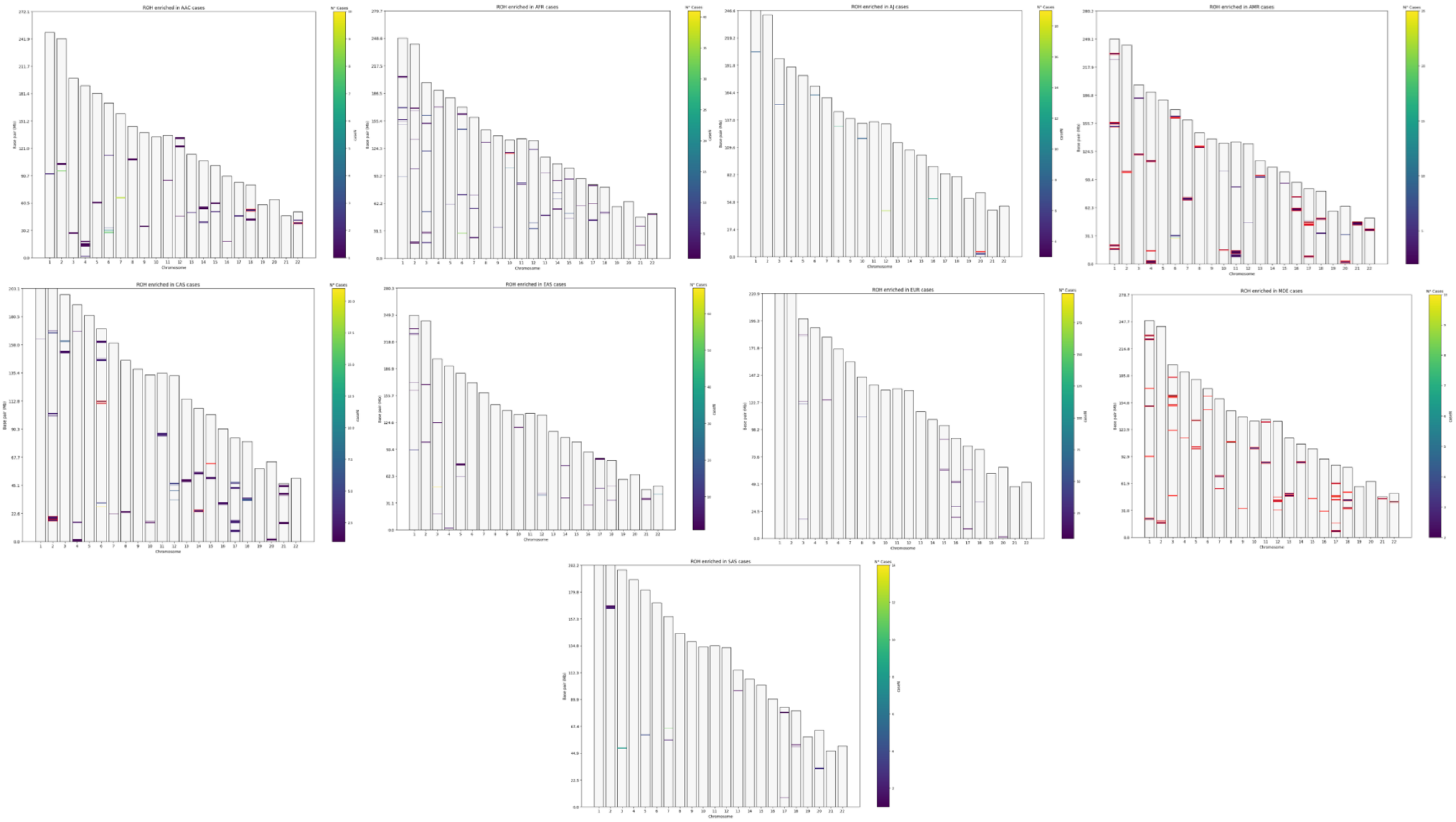

Supplementary Figure 10*: Homozygosity mapping plots displaying runs of homozygosity enriched in cases across diverse ancestries. Highlighted in red are the runs of homozygosity present only in cases. The chromosome is along the x-axis and the runs of homozygosity size in base positions in along the y-axis. AAC = African Admixed, AFR = African, AJ = Ashkenazi Jewish, AMR = American Admixed, CAS = Central Asian, EAS = East Asian, EUR = European, MDE = Middle-Eastern, SAS = South Asian.*

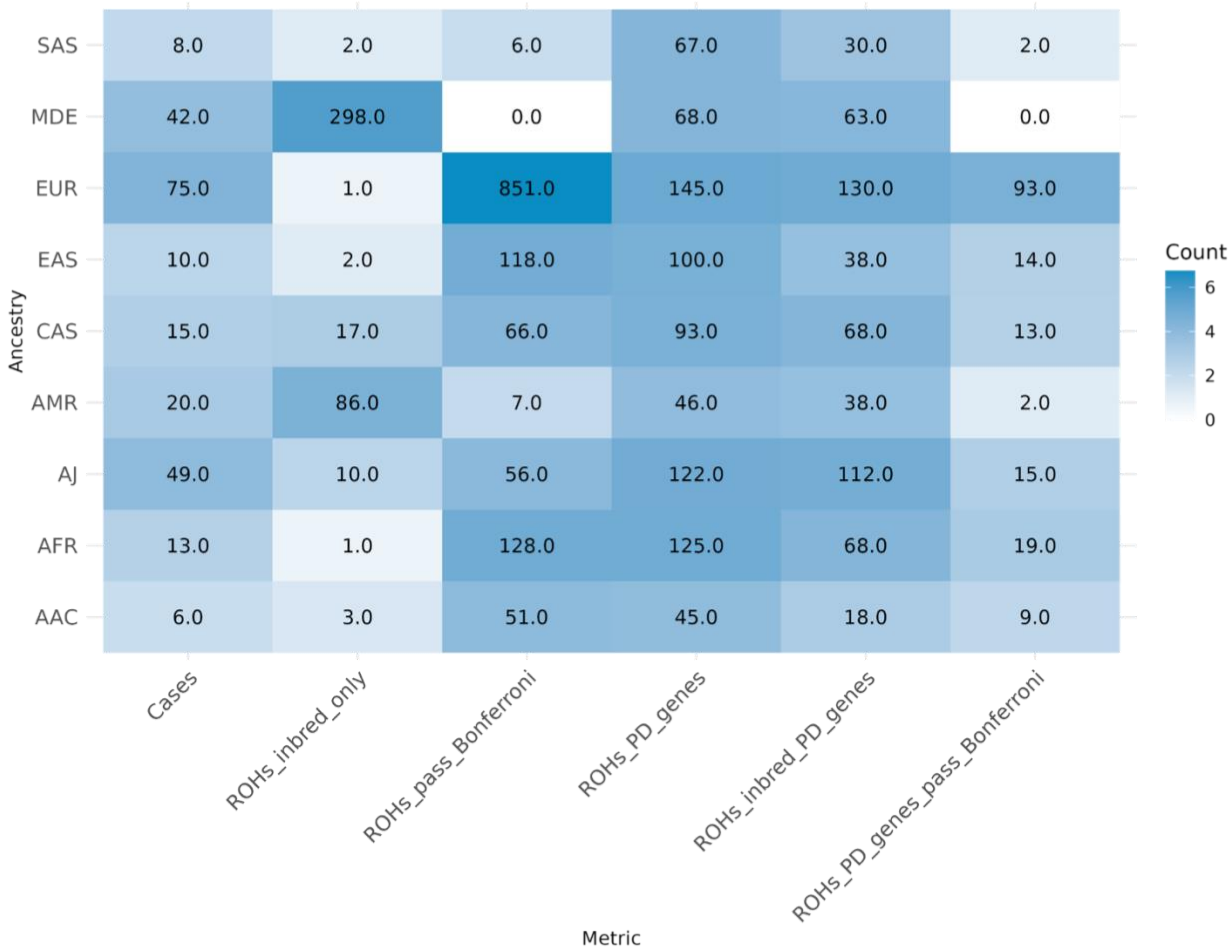

Supplementary Figure 11: *Heatmap illustrating the number of individuals or runs of homozygosity obtained from the homozygosity mapping analysis for individuals with consanguinity. AAC = African Admixed, AFR = African, AJ = Ashkenazi Jewish, AMR = American Admixed, CAS = Central Asian, EAS = East Asian, EUR = European, MDE = Middle-Eastern, SAS = South Asian. The Bonferroni threshold was adjusted by dividing 0.05 by the number of enriched ROHs to account for multiple comparisons.*

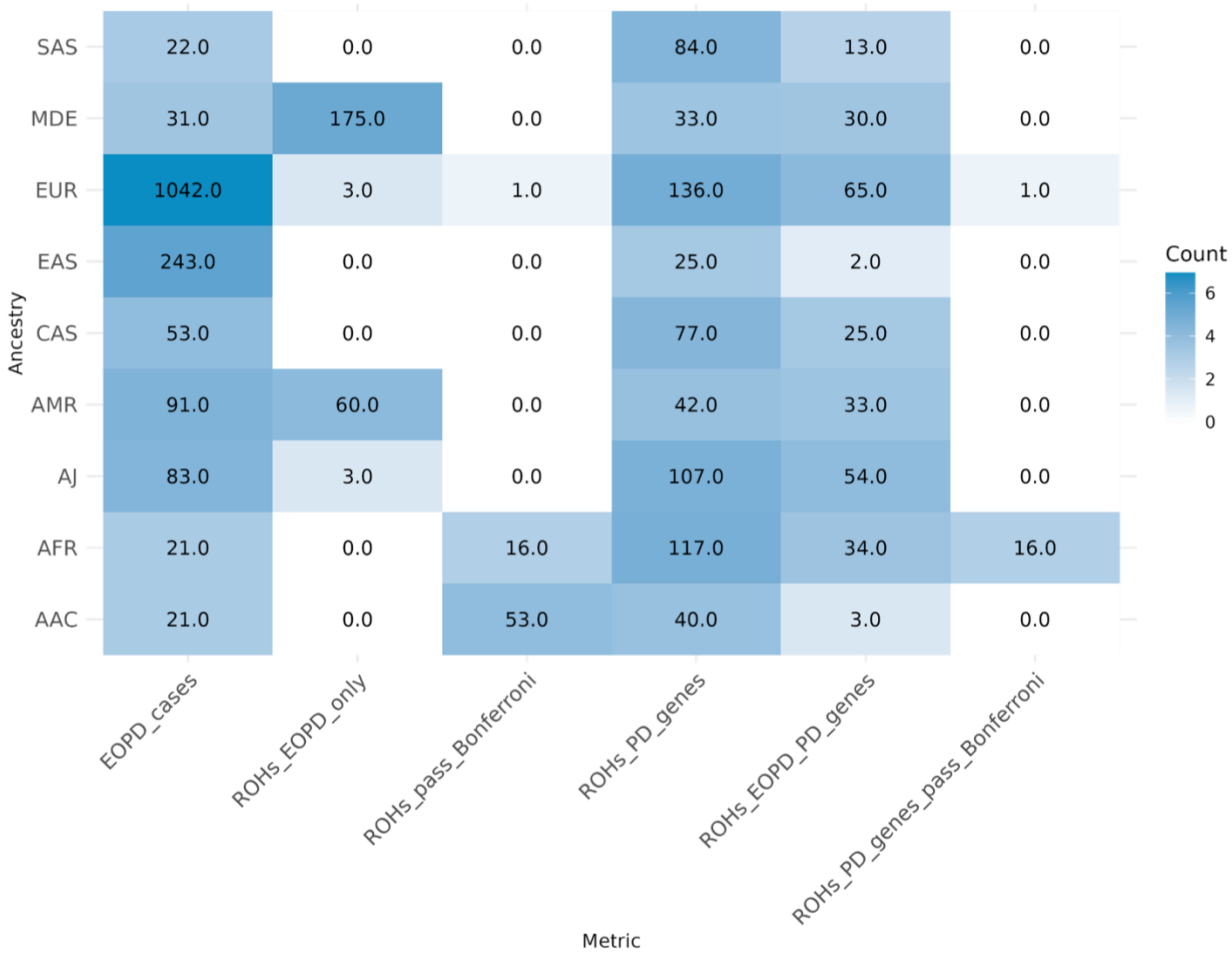

Supplementary Figure 12: *Heatmap illustrating the number of individuals or runs of homozygosity obtained from the homozygosity mapping analysis for early-onset Parkinson's disease patients. AAC = African Admixed, AFR = African, AJ = Ashkenazi Jewish, AMR = American Admixed, CAS = Central Asian, EAS = East Asian, EUR = European, MDE = Middle-Eastern, SAS = South Asian. The Bonferroni threshold was adjusted by dividing 0.05 by the number of enriched ROHs to account for multiple comparisons.*

Supplementary Tables

Supplementary Table 1: *Study participant demographic information for NeuroBooster array genotyping imputed data*

Ancestry	Total participants	Total PD cases	Total controls	Total unrelated participants	Total unrelated cases	Total unrelated controls
**AAC**	1,065	257	808	1,060	256	804
**AFR**	2,603	920	1,683	2,548	917	1,631
**AJ**	1,470	1,011	459	1,459	1,001	458
**AMR**	506	367	139	502	363	139
**CAS**	580	286	294	532	265	267
**EAS**	3,955	1,577	2,378	3,839	1,537	2,302
**EUR**	19,437	11,841	7,596	19,184	11,679	7,505
**MDE**	239	218	21	234	214	20
**SAS**	318	111	207	315	117	198
**Total**	**30,173**	**16,588**	**13,585**	**29,673**	**16,349**	**13,324**
Legend: AAC = African Admixed, AFR = African, AJ = Ashkenazi Jewish, AMR = American Admixed, CAS = Central Asian, EAS = East Asian, EUR = European, MDE = Middle-Eastern, PD = Parkinson's disease, SAS = South Asian						

Supplementary Table 2: *Study participant demographic information for whole-genome sequencing data*

Ancestry	Total participants	Total PD cases	Total controls
**AAC**	41	36	5
**AFR**	208	149	59
**AJ**	165	163	2
**AMR**	155	152	3
**CAS**	71	71	0
**EAS**	1,234	1,219	15
**EUR**	4,005	3,770	235
**MDE**	110	110	0
**FIN**	11	11	0
**SAS**	209	209	0
**Total**	**6,209**	**5,890**	**319**
Legend: AAC = African Admixed, AFR = African, AJ = Ashkenazi Jewish, AMR = American Admixed, CAS = Central Asian, EAS = East Asian, EUR = European, MDE = Middle-Eastern, SAS = South Asian			

Supplementary Table 3: *Parameters used for the genome-wide homozygosity analyses*

**PLINK input flags**	**Flag description**	**Default threshold**	**Threshold used**
**--homozyg**	Performs ROH analysis and generates .hom file	N/A	N/A
**--homozyg-density**	Minimum SNP density (kb/SNP) per ROH segment	50	50
**--homozyg-gap**	Maximum gap between two SNPs before splitting ROH segment	1,000	1,000
**--homozyg-group**	Generates .hom.overlap file	N/A	N/A
**--homozyg-kb**	Minimum length for calling a ROH	1,000	1,500
**--homozyg-snp**	Minimum number of SNPs for calling a ROH	100	100
**--homozyg-window-het**	Maximum number of heterozygous SNPs in a ROH	1	1
**--homozyg-window-missing**	Maximum number of missing SNPs	5	5
**--homozyg-window-snp**	Scanning window size in SNPs	50	50
**--homozyg-window-threshold**	Window threshold for calling a ROH	0.05	0.05
Legend: kb = kilobase, ROH = runs of homozygosity, SNPs = single nucleotide polymorphisms, N/A = not applicable			

Supplementary Table 4: *Missing age information by Ancestry and the corresponding mean age for imputation*

Ancestry	Number of individuals	Individuals with missing age	Age (mean ± SD)	Case age (mean ± SD)	Control age (mean ± SD)
**AAC**	1,060	154	66 ± 10	66 ± 11	66 ± 10
**AFR**	2,548	1,493	63 ± 15	63 ± 12	64 ± 15
**AJ**	1,459	267	69 ± 10	69 ± 10	68 ± 10
**AMR**	502	105	60 ± 12	60 ± 12	63 ± 9
**CAS**	532	152	57 ± 10	59 ± 12	55 ± 6
**EAS**	3,839	1,132	64 ± 11	66 ± 10	62 ± 11
**EUR**	19,184	5,438	66 ± 11	67 ± 11	65 ± 11
**MDE**	234	18	65 ± 13	65 ± 13	67 ± 14
**SAS**	315	109	57 ± 16	62 ± 13	54 ± 17

Legend: AAC = African Admixed, AFR = African, AJ = Ashkenazi Jewish, AMR = American Admixed, CAS = Central Asian, EAS = East Asian, EUR = European, MDE = Middle-Eastern, SAS = South Asian, SD = standard deviation

Supplementary Table 5: *List of the known Parkinson's disease, pallido-pyramidal syndrome, and atypical parkinsonism gene regions and risk loci*

Marker name	Near gene	Source
chr5:75803383-76803383	*SV2C*	(Foo et al., 2020)
chr7:70785507-71785507	*GALNT17*	(Foo et al., 2020)
chr22:49869588-50869588	*PPP6R2*	(Kim et al., 2024)
chr13:109310821-110310821	*RN7SKP10*	(Kim et al., 2024)
chr1:92586630-93586630	*MTF2*	(Kim et al., 2024)
chr17:15830946-16830946	*PIGL*	(Kim et al., 2024)
chr4:2347441-3347441	*ADD1*	(Kim et al., 2024)
chr21:14940233-15940233	*AJ009632.2*	(Kim et al., 2024)
chr15:49995212-50995212	*USP8*	(Kim et al., 2024)
chr17:81587210-82587210	*FASN*	(Kim et al., 2024)
chr20:31333121-32333121	*MYLK2*	(Kim et al., 2024)
chr8:109132545-110132545	*SYBU*	(Kim et al., 2024)

Marker name	Near gene	Source
chr3:178643629-179643629	*AC076966.1*	(Kim et al., 2024)
chr22:40538154-41538154	*RBX1*	(Kim et al., 2024)
chr16:52435514-53435514	*PHBP2*	(Nalls et al., 2019)
chr3:182542285-183542285	*MCCC1*	(Nalls et al., 2019)
chr10:101755522-102755522	*GBF1*	(Nalls et al. 2019)
chr9:17227067-18227067	*SH3GL2*	(Nalls et al. 2019)
chr1:232028865-233028865	*SIPA1L2*	(Nalls et al. 2019)
chr12:122342051-123342051	*HIP1R*	(Nalls et al. 2019)
chr16:30466478-31466478	*SETD1A*	(Nalls et al. 2019)
chr14:54382151-55382151	*GCH1*	(Nalls et al. 2019)
chr6:32110995-33110995	*AHR*	(Nalls et al. 2019)
chr1:154425709-155425709	*PMVK*	(Nalls et al. 2019)
chr1:205268611-206268611	*RAB29*	(Nalls et al. 2019)
chr1:171250629-172250629	*VAMP4*	(Nalls et al. 2019)
chr12:131987182-132987182	*FBRSL1*	(Nalls et al. 2019)
chr17:46289439-47289439	*WNT3*	(Nalls et al. 2019)
chr2:101280501-102280501	*MAP4K4*	(Nalls et al. 2019)
chr3:150891177-151891177	*MED12L*	(Nalls et al. 2019)
chr17:45220942-46220942	*MAPT*	(Nalls et al. 2019)
chr10:119276815-120276815	*INPP5F*	(Nalls et al. 2019)
chr5:134363415-135363415	*AC006077.1*	(Nalls et al. 2019)
chr14:37020065-38020065	*MIPOL1*	(Nalls et al. 2019)
chr11:83276234-84276234	*DLG2*	(Nalls et al. 2019)
chr18:42593415-43593415	*RIT2*	(Nalls et al. 2019)
chr3:48211556-49211556	*IP6K2*	(Nalls et al. 2019)
chr6:71278059-72278059	*RIMS1*	(Nalls et al. 2019)
chr17:6952302-7952302	*MAPT*	(Nalls et al. 2019)
chr8:11354934-12354934	*CTSB*	(Nalls et al. 2019)
chr17:42088995-43088995	*RETREG3*	(Nalls et al. 2019)

Marker name	Near gene	Source
chr4:112947909-113947909	*CAMK2D*	(Nalls et al. 2019)
chr9:17079692-18079692	*SH3GL2*	(Nalls et al. 2019)
chr3:160859842-161859842	*SPTSSB*	(Nalls et al. 2019)
chr2:167753884-168753884	*STK39*	(Nalls et al. 2019)
chr5:60342132-61342132	*ELOVL7*	(Nalls et al. 2019)
chr18:33224354-34224354	*ASXL3*	(Nalls et al. 2019)
chr7:22760430-23760430	*GPNMB*	(Nalls et al. 2019)
chr2:94835195-95835195	*KCNIP3*	(Nalls et al. 2019)
chr8:129389663-130389663	*FAM49B*	(Nalls et al. 2019)
chr21:36980059-37980059	*DYRK1A*	(Nalls et al. 2019)
chr15:61205186-62205186	*lincRNA*	(Nalls et al. 2019)
chr17:43716969-44716969	*UBTF*	(Nalls et al. 2019)
chr8:22168467-23168467	*BIN3*	(Nalls et al. 2019)
chr5:102530090-103530090	*PAM*	(Nalls et al. 2019)
chr16:28433075-29433075	*CD19*	(Nalls et al. 2019)
chr16:52102330-53102330	*CASC16*	(Nalls et al. 2019)
chr4:17467188-18467188	*LCORL*	(Nalls et al. 2019)
chr4:458159-1458159	*TMEM175*	(Nalls et al. 2019)
chr12:39840400-40840400	*LRRK2*	(Nalls et al. 2019)
chr4:89204960-90204960	*AC097478.1*	(Nalls et al. 2019)
chr1:154662560-155662560	*KRTCAP2*	(Nalls et al. 2019)
chr14:74406331-75406331	*RPS6KL1*	(Nalls et al. 2019)
chr11:133417106-134417106	*IGSF9B*	(Nalls et al. 2019)
chr4:75726816-76726816	*FAM47E*	(Nalls et al. 2019)
chr6:27271022-28271022	*RSL24D1P1*	(Nalls et al. 2019)
chr1:226228377-227228377	*ITPKB*	(Nalls et al. 2019)
chr4:15235725-16235725	*BST1*	(Nalls et al. 2019)
chr13:96712767-97712767	*MBNL2*	(Nalls et al. 2019)
chr4:89215479-90215479	*AC097478.1*	(Nalls et al. 2019)

Marker name	Near gene	Source
chr19:1841049-2841049	*SPPL2B*	(Nalls et al. 2019)
chr3:121978045-122978045	*KPNA1*	(Nalls et al. 2019)
chr2:134207046-135207046	*TMEM163*	(Nalls et al. 2019)
chr17:61340005-62340005	*BRIP1*	(Nalls et al. 2019)
chr8:16340084-17340084	*AC011586.2*	(Nalls et al. 2019)
chr17:45166837-46166837	*MAPT*	(Nalls et al. 2019)
chr4:169162006-170162006	*CLCN3*	(Nalls et al. 2019)
chr9:33546393-34546393	*UBAP2*	(Nalls et al. 2019)
chr16:18766171-19766171	*SYT17*	(Nalls et al. 2019)
chr16:50202745-51202745	*NOD2*	(Nalls et al. 2019)
chr1:160999264-161999264	*FCGR2A*	(Nalls et al. 2019)
chr17:77929399-78929399	*DNAH17*	(Nalls et al. 2019)
chr3:28164199-29164199	*RBMS3*	(Nalls et al. 2019)
chr4:75689212-76689212	*SCARB2*	(Nalls et al. 2019)
chr4:75776901-76776901	*FAM47E*	(Nalls et al. 2019)
chr12:45525303-46525303	*SLC38A1*	(Nalls et al. 2019)
chr10:119156173-120156173	*BAG3*	(Nalls et al. 2019)
chr3:17820267-18820267	*TBC1D5*	(Nalls et al. 2019)
chr6:132389222-133389222	*HMGB1P13*	(Nalls et al. 2019)
chr2:17466582-18466582	*KCNS3*	(Nalls et al. 2019)
chr1:154735843-155735843	*GBA*	(Nalls et al. 2019)
chr12:39720632-40720632	*LRRK2*	(Nalls et al. 2019)
chr7:66044864-67044864	*AC006001.3*	(Nalls et al. 2019)
chr20:5525395-6525395	*CRLS1*	(Nalls et al. 2019)
chr11:10037230-11037230	*RNF141*	(Nalls et al. 2019)
chr18:50657219-51657219	*SMAD4*	(Nalls et al., 2019)
chr1:205254444-206254444	*RAB29*	(Nalls et al., 2019)
chr17:43857262-44857262	*FAM171A2*	(Nalls et al., 2019)
chr4:431588-1431588	*GAK*	(Nalls et al., 2019)

Marker name	Near gene	Source
chr10:15015407-16015407	*ITGA8*	(Nalls et al., 2019)
chr6:29640906-30640906	*TRIM40*	(Nalls et al., 2019)
chr13:48853596-49853596	*CAB39L*	(Nalls et al., 2019)
chr14:87497920-88497920	*GPR65*	(Nalls et al., 2019)
chr6:111422088-112422088	*WISP3*	(Nalls et al., 2019)
chr1:154735878-155735878	*GBA1*	(Rizig et al., 2023)
chr14:24713480-25147976	*STXBP6*	(Loesch et al., 2021)
chr4:89695550-89866846	*SNCA*	Known PD genes
chr6:161002496-163072863	*PARK2*	Known PD genes
chr4:41253993-41271316	*UCHL1*	Known PD genes
chr1:7955746-7991189	*DJ-1*	Known PD genes
chr1:155264661-155274879	*GBA*	Known PD genes
chr12:40188943-40405353	*LRRK2*	Known PD genes
chr1:20628940-20656025	*PINK1*	Known PD genes
chr1:16979465-17018421	*ATP13A2*	Known PD genes
chr22:38093930-38199319	*PLA2G6*	Known PD genes
chr22:32468692-32504859	*FBXO7*	Known PD genes
chr3:184309279-184340574	*EIF4G1*	Known PD genes
chr16:46652288-46696621	*VPS35*	Known PD genes
chr1:226481196-226488644	*ITPKB*	Known PD genes
chr2:101375382-101412082	*IL1R2*	Known PD genes
chr2:164231020-164347533	*SCN3A*	Known PD genes
chr3:18306448-18397281	*SATB1*	Known PD genes
chr3:48636411-48648494	*NCKIPSD*	Known PD genes
chr3:52164078-52180311	*ALAS1*	Known PD genes
chr3:52187064-52192147	*TLR9*	Known PD genes
chr3:52282303-52366475	*DNAH1*	Known PD genes
chr3:52366997-52376334	*BAP1*	Known PD genes
chr3:52376641-52389625	*PHF7*	Known PD genes

Marker name	Near gene	Source
chr3:52421102-52459052	*NISCH*	Known PD genes
chr3:52461322-52490479	*STAB1*	Known PD genes
chr3:52760782-52774993	*ITIH3*	Known PD genes
chr3:52789919-52791298	*ITIH4*	Known PD genes
chr4:111896953-112432557	*ANK2*	Known PD genes
chr4:112529876-112840771	*CAMK2D*	Known PD genes
chr5:61455964-61548562	*ELOVL7*	Known PD genes
chr6:27482964-27505339	*ZNF184*	Known PD genes
chr8:11985015-12010720	*CTSB*	Known PD genes
chr8:22694182-22718275	*SORBS3*	Known PD genes
chr8:22721228-22736785	*PDLIM2*	Known PD genes
chr8:22742134-22746637	*C8orf58*	Known PD genes
chr8:22762905-22811607	*BIN3*	Known PD genes
chr9:17579125-17797131	*SH3GL2*	Known PD genes
chr10:15169644-15329060	*FAM171A1*	Known PD genes
chr14:87466670-87526927	*GALC*	Known PD genes
chr16:19056277-19068773	*COQ7*	Known PD genes
chr16:52404096-52513096	*TOX3*	Known PD genes
chr17:44381512-44445214	*ATP6V0A1*	Known PD genes
chr17:44494947-44500427	*PSMC3IP*	Known PD genes
chr17:44581937-44589638	*TUBG2*	Known PD genes
chr3:87178135-87206349	*CHMP2B*	Known PD genes
chr3:183298467-183300020	*MCCC1*	Known PD genes
chr4:75326511-75385910	*FAM47E*	Known PD genes
chr4:15680106-15688816	*FAM200B*	Known PD genes
chr8:17169729-17265135	*MICU3*	Known PD genes
chr10:117891859-117918308	*BAG3*	Known PD genes
chr11:85179700-85179917	*DLG2*	Known PD genes
chr11:134028609-134028686	*MIR4697*	Known PD genes

Marker name	Near gene	Source
chr12:39831272-39975479	*LRRK2*	Known PD genes
chr12:122490673-122495494	*OGFOD2*	Known PD genes
chr14:54375298-54436134	*GCH1*	Known PD genes
chr14:67003549-67048754	*TMEM229B*	Known PD genes
chr15:61560190-61768274	*VPS13C*	Known PD genes
chr16:31063101-31072875	*ZNF646*	Known PD genes
chr18:45687913-45697685	*SYT4*	Known PD genes
chr19:2321518-2328621	*LSM7*	Known PD genes
chr20:3209704-3224039	*DDRGK1*	Known PD genes
chr17:47952550-48146446	*KANSL1*	Known PD genes
chr17:47921884-47922328	*STH*	Known PD genes
chr17:47767469-47769706	*SPPL2C*	Known PD genes
chr17:47706954-47758460	*CRHR1*	Known PD genes
chr17:47351987-47355550	*ARHGAP27*	Known PD genes
chr16:31104433-31120072	*KAT8*	Known PD genes
chr7:23206944-23235489	*GPNMB*	Known PD genes
chr4:15776655-15851607	*CD38*	Known PD genes
chr2:167097510-167391631	*STK39*	Known PD genes
chr7:23142208-23161390	*NUPL2*	Known PD genes
chr7:23066165-23138295	*KLHL7*	Known PD genes
chr6:32669654-32679285	*HLA-DQA1*	Known PD genes
chr6:32584936-32592225	*HLA-DRB6*	Known PD genes
chr4:965099-979768	*DGKQ*	Known PD genes
chr2:134190255-134195627	*CCNT2*	Known PD genes
chr4:938599-964868	*TMEM175*	Known PD genes
chr2:133698188-133961429	*TMEM163*	Known PD genes
chr1:232262219-232425812	*SIPA1L2*	Known PD genes
chr1:205819965-205844048	*SLC41A1*	Known PD genes
chr7:56101569-56106576	*CHCHD2*	Known PD genes

Marker name	Near gene	Source
chr20:3888839-3926958	*PANK2*	Known PD genes
chr2:74361154-74392087	*DCTN1*	Known PD genes
chr15:89316303-89334847	*POLG1*	Known PD genes
chr1:151220451-153220451	*HRNR*	Known PD genes
chr1:153925709-155928394	*PMVK*	Known PD genes
chr1:154162560-156165245	*KRTCAP2*	Known PD genes
chr1:154233158-156235843	*GBAP1*	Known PD genes
chr1:154404265-156406949	*ASH1L*	Known PD genes
chr1:155182384-157185070	*SEMA4A*	Known PD genes
chr1:160499264-162499264	*FCGR2A*	Known PD genes
chr1:170750628-172750629	*VAMP4*	Known PD genes
chr1:206009146-206268644	*NUCKS1*	Known PD genes
chr1:206009146-206268644	*RAB29*	Known PD genes
chr1:225728376-227728377	*ITPKB*	Known PD genes
chr1:231528865-233528865	*SIPA1L2*	Known PD genes
chr2:16966581-18966570	*KCNS3*	Known PD genes
chr9:40365644-40365693	*KCNIP3*	Known PD genes
chr2:100780501-102780504	*MAP4K4*	Known PD genes
chr2:133681218-135681219	*TMEM163*	Known PD genes
chr2:167253884-169253884	*STK39*	Known PD genes
chr3:17320267-19320267	*SATB1*	Known PD genes
chr3:27664199-29664199	*LINC00693*	Known PD genes
chr3:47707499-49711556	*IP6K2*	Known PD genes
chr3:121478045-123478045	*KPNA1*	Known PD genes
chr3:150391178-152391176	*MED12L*	Known PD genes
chr3:160359842-162359842	*SPTSSB*	Known PD genes
chr3:182042285-184042285	*MCCC1*	Known PD genes
chr4:849276-932373	*GAK*	Known PD genes
chr4:932387-958656	*TMEM175*	Known PD genes

Marker name	Near gene	Source
chr4:14735724-16735725	*BST1*	Known PD genes
chr4:16967188-18967188	*LCORL*	Known PD genes
chr4:75185155-77189212	*SCARB2*	Known PD genes
chr4:75222759-77226816	*FAM47E*	Known PD genes
chr4:75272844-77276901	*FAM47E-STBD1*	Known PD genes
chr4:88744890-90685975	*SNCA*	Known PD genes
chr4:112447909-114447909	*CAMK2D*	Known PD genes
chr4:168662006-170662006	*CLCN3*	Known PD genes
chr5:59842133-61842132	*ELOVL7*	Known PD genes
chr5:102030090-104030093	*PAM*	Known PD genes
chr5:123774579-125774580	*ZNF608*	Known PD genes
chr5:133863414-135863416	*C5orf24*	Known PD genes
chr6:26737023-28771024	*LOC100131289*	Known PD genes
chr6:29140906-31140906	*TRIM40*	Known PD genes
chr6:30878457-32878457	*SLC44A4*	Known PD genes
chr6:31610995-33610995	*HLA-DRB5*	Known PD genes
chr6:70778059-72778039	*RIMS1*	Known PD genes
chr6:110922088-112922089	*FYN*	Known PD genes
chr6:131889221-133889223	*RPS12*	Known PD genes
chr7:22260430-24260430	*GPNMB*	Known PD genes
chr7:65544938-67544864	*GS1-124K5.11*	Known PD genes
chr8:10854933-12854934	*CTSB*	Known PD genes
chr8:15840084-17840084	*FGF20*	Known PD genes
chr8:21668468-23668467	*BIN3*	Known PD genes
chr8:128889663-130889663	*FAM49B*	Known PD genes
chr9:16727067-18579692	*SH3GL2*	Known PD genes
chr9:33046393-35046394	*UBAP2*	Known PD genes
chr10:14515407-16515407	*ITGA8*	Known PD genes
chr10:101255522-103255522	*GBF1*	Known PD genes

Marker name	Near gene	Source
chr10:118656173-120656173	*BAG3*	Known PD genes
chr10:118776815-120776815	*INPP5F*	Known PD genes
chr11:9537230-11537230	*RNF141*	Known PD genes
chr11:82776235-84776234	*DLG2*	Known PD genes
chr11:132917106-134917107	*IGSF9B*	Known PD genes
chr12:31657825-33657824	*FGD4*	Known PD genes
chr12:31826971-33826970	*PKP2*	Known PD genes
chr12:33792942-35252126	*ALG10*	Known PD genes
chr12:39528770-41220632	*LRRK2*	Known PD genes
chr12:40053867-41542837	*CNTN1*	Known PD genes
chr12:40477002-42477002	*PDZRN4*	Known PD genes
chr12:41141601-42901976	*GXYLT1*	Known PD genes
chr12:45025303-47025303	*SCAF11*	Known PD genes
chr12:121888692-123842051	*HIP1R*	Known PD genes
chr12:132241105-132241213	*FBRSL1*	Known PD genes
chr13:48353596-50353596	*CAB39L*	Known PD genes
chr13:96212767-98212767	*MBNL2*	Known PD genes
chr14:36520065-38520066	*MIPOL1*	Known PD genes
chr14:53882151-55882151	*GCH1*	Known PD genes
chr14:73906331-75906691	*RPS6KL1*	Known PD genes
chr14:86997920-88997920	*GALC*	Known PD genes
chr15:60705186-62705186	*VPS13C*	Known PD genes
chr16:18183636-20266171	*SYT17*	Known PD genes
chr16:27933075-29933075	*CD19*	Known PD genes
chr16:29966478-31966478	*SETD1A*	Known PD genes
chr16:49702745-51702745	*NOD2*	Known PD genes
chr16:51602331-53602330	*CASC16*	Known PD genes
chr16:51935514-53935514	*CHD9*	Known PD genes
chr16:57553760-59553768	*CNOT1*	Known PD genes

Marker name	Near gene	Source
chr17:6452301-8452303	*CHRNB1*	Known PD genes
chr17:41584761-43663645	*RETREG3*	Known PD genes
chr17:43142320-45216970	*UBTF*	Known PD genes
chr17:43357262-45357264	*FAM171A2*	Known PD genes
chr17:44339905-46339907	*MAP3K14*	Known PD genes
chr17:44720940-46666837	*CRHR1*	Known PD genes
chr17:44855939-46855941	*MAPT-AS1*	Known PD genes
chr17:45111699-47111701	*KANSL1*	Known PD genes
chr17:45723580-47723580	*NSF*	Known PD genes
chr17:45789439-47789439	*WNT3*	Known PD genes
chr17:60840005-62840005	*BRIP1*	Known PD genes
chr17:77429398-79429398	*DNAH17*	Known PD genes
chr18:32724355-34724354	*ASXL3*	Known PD genes
chr18:42093416-44093415	*RIT2*	Known PD genes
chr18:50157219-52157219	*MEX3C*	Known PD genes
chr19:1341048-3341049	*SPPL2B*	Known PD genes
chr20:2184040-4184039	*DDRGK1*	Known PD genes
chr20:5025395-7025394	*CRLS1*	Known PD genes
chr21:36480063-38480438	*DYRK1A*	Known PD genes
chr16:74212955-75274831	*FA2H*	Pallido-pyramidal syndrome (Simón-Sánchez et al., 2012)
chr21:43219094-44262307	*PDXK*	Pallido-pyramidal syndrome (Simón-Sánchez et al., 2012)
chr15:44054818-45163688	*SPG11*	Pallido-pyramidal syndrome (Simón-Sánchez et al., 2012)
chr6:146043833-147054953	*RAB32*	Known PD gene (Hop et al., 2024)
chr20:613240-1689415	*PSMF1*	Known PD gene (Magrinelli et al., 2024)
Legend: chr = chromosome, PD = Parkinson's disease. Positions on GRCh38.		

Supplementary Table 6: *Putative family structures identified in the Middle-Eastern group for runs of homozygosity segregating in families*

FID	Phenotype	Family structure/relationship	Sex
KING1	Case		M
	Case		M
KING2	Case		F
	Control		F
	Control		M
KING3	Case		M
	Control		M
KING4	Case	Sibship	M
	Case		M
KING5	Case		F
	Case		F
	Case		F
Legend: FID = family ID, M = male, F = female			

Supplementary Table 7: *T-test results for measures of genome-wide homozygosity in Parkinson's disease*

**Ancestry**	**SROH**			**AVROH**			**NROH**			**FROH**		
	**Cases (mean ± SE)**	**Controls (mean ± SE)**	**p-value**	**Cases (mean ± SE)**	**Controls (mean ± SE)**	**p-value**	**Cases (mean ± SE)**	**Controls (mean ± SE)**	**p-value**	**Cases (mean ± SE)**	**Controls (mean ± SE)**	**p-value**
**AAC**	6330.5 ± 989.3	4373.8 ± 378.8	0.066	1815.6 ± 84.6	1580.9 ± 40.8	**0.013***	2.1 ± 0.2	1.7 ± 0.1	0.094	0.0022 ± 3E-04	0.0015 ± 1E-04	0.066
**AFR**	9820.3 ± 456.3	8158.4 ± 297.7	**0.00232**	2252.6 ± 28.4	2118.8 ± 23.4	**2.8E-04*****	4 ± 0.1	3.3 ± 0.1	**4.92E-05*****	0.0034 ± 2E-04	0.0028 ± 1E-04	**0.002****
**AJ**	22595 ± 816.2	21501.1 ± 820	0.345	2879.5 ± 27.5	2826.3 ± 39.7	0.270	7.3 ± 0.2	7.2 ± 0.2	0.770	0.0078 ± 3E-04	0.0074 ± 3E-04	0.345
**AMR**	15162.8 ± 1491.6	13186.3 ± 1351	0.327	2302.8 ± 64.8	2483.4 ± 128.7	0.212	5.1 ± 0.3	4.6 ± 0.3	0.207	0.0053 ± 5E-04	0.0046 ± 5E-04	0.327
**CAS**	13658.5 ± 2093.5	19719.4 ± 2506.5	0.064	1885 ± 51.6	2087.3 ± 52.6	**0.006****	5.2 ± 0.6	6.8 ± 0.7	0.073	0.0047 ± 7E-04	0.0068 ± 9E-04	0.064
**EAS**	5188.6 ± 201.3	7134.8 ± 197	**5.72E-12**	1735.7 ± 20.4	1854 ± 15	**2.93E-06*****	2.5 ± 0.1	3.4 ± 0.1	**8.47E-14*****	0.0018 ± 7E-04	0.0025 ± 7E-04	**5.68E-12*****
**EUR**	6391.1 ± 74.5	7053.2 ± 126.2	**6.29E-06**	1907.7 ± 6.8	1936.8 ± 8.9	**0.009****	3 ± 0	3.2 ± 0	**3.1E-04*****	0.0022 ± 3E-04	0.0024 ± 4E-04	**6.3E-06*****
**MDE**	26001.8 ± 2808	11143.3 ± 3046	**6.67E-04**	2048.5 ± 50.3	1814.9 ± 206.5	0.284	10.4 ± 1	4.6 ± 1	**6.8E-05*****	0.009 ± 0.001	0.0039 ± 0.001	**6.67E-04*****
**SAS**	12647.7 ± 2021.5	34198.7 ± 4113.8	**4.08E-06**	1995.5 ± 62.9	2155 ± 66	0.081	5.3 ± 0.7	11.8 ± 1.3	**1.34E-05*****	0.0044 ± 7E-04	0.0118 ± 0.001	**4.08E-06*****

Legend: AAC = African Admixed, AFR = African, AJ = Ashkenazi Jewish, AMR = American Admixed, AV_{ROH} = average length of runs of homozygosity, CAS = Central Asian, EAS = East Asian, EUR = European, F_{ROH} = runs of homozygosity-based estimates for inbreeding coefficient, MDE = Middle-Eastern, N_{ROH} = number of runs of homozygosity, SAS = South Asian, SE = standard error, S_{ROH} = total length of runs of homozygosity. *p*-values indicate the statistical significance as following $p < 0.001$:***, $p < 0.01$:**, $p < 0.05$:*, $p \geq 0.05$: not significant

Supplementary Table 8: *Measures of genome-wide homozygosity in Parkinson's disease excluding Parkinson's disease recessive genes (PRKN, PINK1, and DJ-1)*

**Ancestry**	**SROH**		**AVROH**		**NROH**		**FROH**	
	**Beta (mean ± SE)**	**p-value**	**Beta (mean ± SE)**	**p-value**	**Beta (mean ± SE)**	**p-value**	**Beta (mean ± SE)**	**p-value**
**AAC**	1.31E-05 ± 6.16E-06	**0.034**	1.76E-04 ± 6.23E-05	**0.005**	0.048 ± 0.027	0.077	37.8 ± 17.8	**0.034**
**AFR**	7.13E-06 ± 3.45E-06	**0.039**	1.19E-04 ± 5.1E-05	**0.019**	0.022 ± 0.012	0.056	20.6 ± 9.97	**0.039**
**AJ**	2.02E-06 ± 2.66E-06	0.448	6.51E-05 ± 6.77E-05	0.336	0.003 ± 0.013	0.828	5.82 ± 7.67	0.448
**AMR**	4.34E-06 ± 4.61E-06	0.347	-8.66E-05 ± 7.64E-05	0.257	0.0231 ± 0.022	0.300	12.5 ± 13.3	0.347
**CAS**	-2.69E-07 ± 2.87E-06	0.925	-1.56E-04 ± 1.21E-04	0.196	-9.25E-04 ± 0.010	0.927	-0.775 ± 8.27	0.925
**EAS**	-9.94E-06 ± 5.41E-06	0.066	-4.94E-05 ± 6.26E-05	0.430	-0.026 ± 0.014	0.059	-28.7 ± 15.6	0.066
**EUR**	-8.48E-06 ± 1.68E-06	**4.51E-07**	-6.01E-05 ± 2E-05	**0.003**	-0.023 ± 0.005	**1.89E-05**	-24.5 ± 4.85	**4.52E-07**
**MDE**	4.04E-05 ± 2.1E-05	0.054	4.54E-04 ± 3.02E-04	0.133	0.115 ± 0.056	**0.042**	117 ± 60.6	0.054
**SAS**	-1.67E-05 ± 4.83E-06	**5.44E-04**	-2.61E-04 ± 1.52E-04	0.086	-0.045 ± 0.013	**7.81E-04**	-48.2 ± 13.9	**5.44E-04**

Legend: AAC = African Admixed, AFR = African, AJ = Ashkenazi Jewish, AMR = American Admixed, AV_{ROH} = average length of runs of homozygosity, CAS = Central Asian, EAS = East Asian, EUR = European, F_{ROH} = runs of homozygosity-based estimates for inbreeding coefficient, MDE = Middle-Eastern, N_{ROH} = number of runs of homozygosity, SAS = South Asian, SE = standard error, S_{ROH} = total length of runs of homozygosity. *p*-values indicate the statistical significance as following $p < 0.001$:***, $p < 0.01$:**, $p < 0.05$:*, $p \geq 0.05$: not significant

Supplementary Table 9*: Linear regression for runs of homozygosity associated with Parkinson's disease at age of onset*

**Ancestry**	**S_{ROH}**			**AV_{ROH}**			**N_{ROH}**			**F_{ROH}**		
	**Cases (mean ± SE)**	**beta**	**p-value**	**Cases (mean ± SE)**	**beta**	**p-value**	**Cases (mean ± SE)**	**beta**	**p-value**	**Cases (mean ± SE)**	**beta**	**p-value**
**AAC**	6330.5 ± 989.3	7.86E-06	0.793	1815.6 ± 84.6	-7.91E-04	**0.035***	2.1 ± 0.2	-0.0131	0.939	0.0022 ± 0.00034	22.7	0.792
**AFR**	9820.3 ± 456.3	1.55E-05	0.426	2252.6 ± 28.4	-2.55E-04	0.642	4 ± 0.1	0.0266	0.778	0.0034 ± 0.00016	44.8	0.425
**AJ**	22595 ± 816.2	-4.37E-06	0.631	2879.5 ± 27.5	4.79E-04	0.082	7.3 ± 0.2	-0.0549	0.281	0.0078 ± 0.00028	-12.6	0.631
**AMR**	15162.8 ± 1491.6	-5.51E-05	**3.87E-4*** **	2302.8 ± 64.8	-8.78E-04	**0.019***	5.1 ± 0.3	-0.235	**0.003***	0.0053 ± 0.00052	-159.0	**3.73E-4*** **
**CAS**	13658.5 ± 2093.5	-2.80E-05	0.554	1885 ± 51.6	2.30E-04	0.767	5.2 ± 0.6	-0.0698	0.656	0.0047 ± 0.00073	-80.9	0.554
**EAS**	5188.6 ± 201.3	-2.14E-05	0.607	1735.7 ± 20.4	3.03E-04	0.406	2.5 ± 0.1	-0.0382	0.716	0.0018 ± 7e-05	-61.7	0.608
**EUR**	6391.1 ± 74.5	-2.04E-05	**0.027***	1907.7 ± 6.8	-6.23E-05	0.569	3 ± 0	-0.0499	0.094	0.0022 ± 2.6e-05	-58.9	**0.027***
**MDE**	26001.8 ± 2808	-6.00E-06	0.609	2048.5 ± 50.3	7.68E-04	0.217	10.4 ± 1	-0.0290	0.390	0.009 ± 0.00097	-17.3	0.609
**SAS**	12647.7 ± 2021.5	-7.17E-05	0.282	1995.5 ± 62.9	4.47E-04	0.801	5.3 ± 0.7	-0.251	0.174	0.0044 ± 7e-04	-207.0	0.282

Legend: AAC = African Admixed, AFR = African, AJ = Ashkenazi Jewish, AMR = American Admixed, AV_{ROH} = average length of runs of homozygosity, CAS = Central Asian, EAS = East Asian, EUR = European, F_{ROH} = runs of homozygosity-based estimates for inbreeding coefficient, MDE = Middle-Eastern, N_{ROH} = number of runs of homozygosity, SAS = South Asian, SE = standard error, S_{ROH} = total length of runs of homozygosity. p-values indicate the statistical significance as following p < 0.001:***, p < 0.01:**, p < 0.05:*, p ≥ 0.05: not significant

Supplementary Table 10: *Runs of homozygosity associated with Parkinson's disease at age of onset excluding Parkinson's disease recessive genes (PRKN, PINK1, and DJ-1)*

Ancestry	S_{ROH}			AV_{ROH}			N_{ROH}			F_{ROH}		
	**Cases (mean ± SE)**	**beta**	**p-value**	**Cases (mean ± SE)**	**beta**	**p-value**	**Cases (mean ± SE)**	**beta**	**p-value**	**Cases (mean ± SE)**	**beta**	**p-value**
**AAC**	6330.5 ± 989.3	7.86E-06	0.793	1815.6 ± 84.6	-7.91E-04	**0.036***	2.1 ± 0.2	-0.0131	0.939	0.0022 ± 0.00034	22.7	0.792
**AFR**	9814.6 ± 455.3	1.54E-05	0.430	2252.7 ± 28.4	-2.54E-04	0.644	4 ± 0.1	0.0252	0.789	0.0034 ± 0.00016	44.6	0.429
**AJ**	22579.8 ± 815.6	-4.41E-06	0.628	2878.5 ± 27.5	4.76E-04	0.084	7.3 ± 0.2	-0.0549	0.280	0.0078 ± 0.00028	-12.8	0.628
**AMR**	15143.2 ± 1486.5	-5.54E-05	**3.68E-4*****	2302.8 ± 64.9	-8.80E-04	**0.019***	5.1 ± 0.3	-0.237	**0.003****	0.0052 ± 0.00051	-160	**3.69E-4*****
**CAS**	13651.4 ± 2090.8	-2.80E-05	0.554	1885.2 ± 51.6	2.30E-04	0.767	5.2 ± 0.6	-0.0698	0.656	0.0047 ± 0.00072	-80.9	0.554
**EAS**	5187.5 ± 201	-2.08E-05	0.620	1735.7 ± 20.4	3.01E-04	0.409	2.5 ± 0.1	-0.0354	0.737	0.0018 ± 7E-05	-59.8	0.620
**EUR**	6389.6 ± 74.5	-2.02E-05	**0.029***	1907.8 ± 6.8	-6.24E-05	0.568	3 ± 0	-0.0490	0.101	0.0022 ± 2.6E-05	-58.3	**0.029***
**MDE**	25964.3 ± 2807.1	-5.94E-06	0.612	2049.4 ± 50.4	7.72E-04	0.214	10.4 ± 1	-0.0288	0.394	0.009 ± 0.00097	-17.1	0.612
**SAS**	12647.6 ± 2021.5	-7.16E-05	0.282	1995.5 ± 62.9	4.48E-04	0.801	5.3 ± 0.7	-0.251	0.174	0.0044 ± 7E-04	-207	0.252

Legend: AAC = African Admixed, AFR = African, AJ = Ashkenazi Jewish, AMR = American Admixed, AV_{ROH} = average length of runs of homozygosity, CAS = Central Asian, EAS = East Asian, EUR = European, F_{ROH} = runs of homozygosity-based estimates for inbreeding coefficient, MDE = Middle-Eastern, N_{ROH} = number of runs of homozygosity, SAS = South Asian, SE = standard error, S_{ROH} = total length of runs of homozygosity. p-values indicate the statistical significance as following $p < 0.001$:***, $p < 0.01$:**, $p < 0.05$:*, $p \geq 0.05$: not significant

Supplementary Table 11*: Runs of homozygosity in cases subsetted for different age of onset of Parkinson's disease versus controls*

**Ancestry**	**AAO**	**Number of cases (controls)**	**S_{ROH}**		**AV_{ROH}**		**N_{ROH}**		**F_{ROH}**	
			**Beta (mean ± SE)**	**p-value**	**Beta (mean ± SE)**	**p-value**	**Beta (mean ± SE)**	**p-value**	**Beta (mean ± SE)**	**p-value**
**AAC**	<35	3 (804)	4.73E-06 ± 7.15E-05	0.947	9.94E-04 ± 3.82E-04	**0.009****	-0.12 ± 0.374	0.749	13.7 ± 206	0.947
	35-44	18 (804)	2.89E-05 ± 1.71E-05	0.092	6.34E-04 ± 2.37E-04	**0.007****	0.093 ± 0.077	0.231	83.3 ± 49.4	0.092
	45-54	34 (804)	-1.85E-06 ± 2.57E-05	0.943	2.66E-04 ± 1.47E-04	0.070	-0.026 ± 0.096	0.790	-5.4 ± 74.1	0.942
	55-65	36 (804)	-1.77E-06 ± 2.61E-05	0.946	-1.3E-04 ± 1.74E-04	0.453	-0.037 ± 0.099	0.709	-5.21 ± 75.4	0.945
	>65	58 (804)	1.68E-05 ± 9.21E-06	0.069	2.97E-04 ± 1.32E-04	**0.024***	0.082 ± 0.0491	0.095	48.4 ± 26.6	0.069
**AFR**	<35	8 (1631)	8.25E-06 ± 1.33E-05	0.534	2.05E-04 ± 4.22E-04	0.627	0.039 ± 0.055	0.479	23.8 ± 38.3	0.534
	35-44	13 (1631)	3.86E-06 ± 2.91E-05	0.895	2.02E-04 ± 4.22E-04	0.632	-0.023 ± 0.102	0.821	11.2 ± 83.9	0.894
	45-54	43 (1631)	-2.24E-06 ± 1.67E-05	0.893	-3.63E-05 ± 2.08E-04	0.862	0.012 ± 0.042	0.780	-6.43 ± 48.2	0.894
	55-65	50 (1631)	-1.65E-05 ± 2.02E-05	0.416	8.73E-05 ± 1.8E-04	0.627	-0.037 ± 0.053	0.491	-47.4 ± 58.3	0.416
	>65	44 (1631)	-3.62E-05 ± 2.69E-05	0.179	-1.14E-04 ± 1.93E-04	0.554	-0.082 ± 0.069	0.234	-104 ± 77.7	0.179
**AJ**	<35	10 (458)	7.87E-06 ± 1.45E-05	0.588	4.32E-04 ± 3.1E-04	0.163	0.019 ± 0.089	0.835	22.7 ± 41.9	0.587

	35-44	73 (458)	6.99E-06 ± 8.72E-06	0.423	3.17E-05 ± 2.09E-04	0.880	0.030 ± 0.041	0.461	20.2 ± 25.2	0.423
	45-54	151 (458)	3.94E-06 ± 3.68E-06	0.284	-3.13E-05 ± 1.38E-04	0.821	0.017 ± 0.022	0.421	11.4 ± 10.6	0.284
	55-65	258 (458)	-6.55E-06 ± 5.84E-06	0.263	4.63E-05 ± 9.36E-05	0.620	-0.028 ± 0.023	0.237	-18.9 ± 16.9	0.263
	>65	310 (458)	4.62E-06 ± 4E-06	0.248	1.26E-04 ± 9.95E-05	0.206	0.013 ± 0.020	0.527	13.3 ± 11.5	0.248
**AMR**	<35	29 (139)	2.74E-05 ± 1.65E-05	0.096	1.59E-04 ± 1.75E-04	0.363	0.177 ± 0.081	**0.028***	79 ± 47.5	0.096
	35-44	62 (139)	9.2E-06 ± 7.65E-06	0.229	3.34E-05 ± 1.45E-04	0.818	0.044 ± 0.037	0.230	26.5 ± 22.1	0.229
	45-54	69 (139)	8.22E-06 ± 9.12E-06	0.367	-3.2E-05 ± 1.23E-04	0.794	0.035 ± 0.042	0.400	23.7 ± 26.3	0.367
	55-65	53 (139)	3.84E-06 ± 8.6E-06	0.655	-1.25E-04 ± 1.66E-04	0.452	0.03 ± 0.04	0.453	11.1 ± 24.8	0.655
	>65	41 (139)	1.23E-05 ± 1.38E-05	0.371	2.17E-04 ± 1.6E-04	0.176	0.065 ± 0.058	0.269	35.6 ± 39.7	0.371
**CAS**	<35	12 (267)	-2.12E-05 ± 4.26E-05	0.619	2.53E-04 ± 5.49E-04	0.645	-0.070 ± 0.131	0.591	-61.1 ± 123	0.619
	35-44	41 (267)	-3.09E-06 ± 7.69E-06	0.688	-3.72E-04 ± 2.26E-04	0.099	-0.010 ± 0.027	0.719	-8.9 ± 22.2	0.688
	45-54	23 (267)	-1.38E-05 ± 2.06E-05	0.504	2.45E-04 ± 3.28E-04	0.456	-0.046 ± 0.063	0.467	-39.8 ± 59.6	0.504
	55-65	24 (267)	-1.34E-04 ± 6.25E-05	**0.032***	-3.93E-04 ± 3.58E-04	0.273	-0.287 ± 0.128	**0.024***	-387 ± 180	**0.032***
	>65	10 (267)	-0.00446 ± 0.726	0.995	-1.55E-04 ± 1.69E-04	0.361	-11.1 ± 1760	0.995	-12900 ± 2100000	0.995

	<35	59 (2302)	2.74E-05 ± 1.84E-05	0.136	1.48E-05 ± 2.55E-04	0.954	0.067 ± 0.044	0.123	79.1 ± 53.1	0.136
	35-44	184 (2302)	-4.58E-06 ± 1.45E-05	0.753	5.47E-05 ± 1.75E-04	0.754	-0.009 ± 0.037	0.807	-13.2 ± 42	0.754
	45-54	237 (2302)	-4.18E-05 ± 2.51E-05	0.096	-9.98E-05 ± 1.47E-04	0.497	-0.086 ± 0.054	0.115	-121 ± 72.6	0.096
	55-65	112 (2302)	-2.59E-05 ± 2.38E-05	0.276	-2.73E-04 ± 1.66E-04	0.100	-0.043 ± 0.050	0.383	-74.9 ± 68.8	0.276
**EAS**	>65	106 (2302)	-3.47E-06 ± 1.58E-05	0.826	-5.62E-06 ± 2.15E-04	0.979	-0.021 ± 0.041	0.613	-9.98 ± 45.6	0.827
	<35	244 (7505)	8.4E-06 ± 4.3E-06	0.051	-4.9E-05 ± 9.77E-05	0.616	0.027 ± 0.016	0.091	24.2 ± 12.4	0.051
	35-44	798 (7505)	-1.15E-05 ± 5.74E-06	**0.044***	-1.26E-04 ± 5.59E-05	**0.025***	-0.039 ± 0.018	**0.027***	-33.3 ± 16.6	**0.045***
	45-54	1645 (7505)	-1.18E-05 ± 4.12E-06	**0.004****	-8.88E-05 ± 3.79E-05	**0.019***	-0.025 ± 0.011	**0.024***	-33.9 ± 11.9	**0.004****
	55-65	2360 (7505)	-1.07E-05 ± 3.18E-06	**7.49E-04*****	-5.89E-05 ± 3.21E-05	0.066	-0.023 ± 0.009	**0.011***	-30.9 ± 9.18	**7.5E-04*****
**EUR**	>65	2395 (7505)	-5.64E-06 ± 2.95E-06	0.056	-1.57E-05 ± 3.74E-05	0.675	-0.022 ± 0.010	**0.031***	-16.3 ± 8.52	0.056
	<35	11 (20)	6.51E-04 ± 2.08	1	0.01 ± 328	1	1.59 ± 5160	1	1880 ± 6010000	1
	35-44	20 (20)	3.53E-05 ± 2.75E-05	0.2	-3.42E-04 ± 7.88E-04	0.664	0.098 ± 0.077	0.202	102 ± 79.4	0.200
	45-54	40 (20)	3.66E-05 ± 2.06E-05	0.076	1.84E-05 ± 4.12E-04	0.964	0.104 ± 0.060	0.082	106 ± 59.6	0.076
**MDE**	55-65	46 (20)	2.88E-05 ± 2.2E-05	0.191	2.06E-04 ± 5.15E-04	0.689	0.088 ± 0.064	0.169	83.1 ± 63.5	0.191

	>65	42 (20)	1.44E-05 ± 3.02E-05	0.632	6.13E-04 ± 4.62E-04	0.184	0.065 ± 0.092	0.484	41.7 ± 87.1	0.632
	<35	4 (198)	-6.77E-06 ± 1.99E-05	0.734	-5.83E-04 ± 7.13E-04	0.413	-0.007 ± 0.048	0.892	-19.5 ± 57.4	0.734
	35-44	18 (198)	-2.79E-05 ± 1.61E-05	0.084	-1.15E-04 ± 3.28E-04	0.726	-0.064 ± 0.038	0.090	-80.4 ± 46.6	0.084
	45-54	11 (198)	-6.37E-06 ± 8.12E-06	0.433	-3.03E-04 ± 3.73E-04	0.416	-0.016 ± 0.024	0.499	-18.4 ± 23.4	0.433
	55-65	12 (198)	-2.15E-05 ± 1.67E-05	0.199	2.89E-04 ± 4.04E-04	0.474	-0.074 ± 0.055	0.182	-62.1 ± 48.3	0.199
**SAS**	>65	17 (198)	-3.23E-05 ± 2.29E-05	0.158	-2.27E-04 ± 4.99E-04	0.649	-0.093 ± 0.066	0.158	-93.3 ± 66.1	0.158

Legend: AAC = African Admixed, AFR = African, AJ = Ashkenazi Jewish, AMR = American Admixed, AV_{ROH} = average length of runs of homozygosity, CAS = Central Asian, EAS = East Asian, EUR = European, F_{ROH} = runs of homozygosity-based estimates for inbreeding coefficient, MDE = Middle-Eastern, N_{ROH} = number of runs of homozygosity, SAS = South Asian, SE = standard error, S_{ROH} = total length of runs of homozygosity. p-values indicate the statistical significance as following p < 0.001:***, p < 0.01:**, p < 0.05:*, p ≥ 0.05: not significant

Supplementary Table 12: *T-test results for runs of homozygosity in cases subsetted for early-onset Parkinson's disease and late-onset Parkinson's disease versus controls*

Ancestry	AAO	Number of cases (controls)	S_{ROH}			AV_{ROH}			N_{ROH}			F_{ROH}		
			**Cases (mean ± SE)**	**Controls (mean ± SE)**	**p-value**	**Cases (mean ± SE)**	**Controls (mean ± SE)**	**p-value**	**Cases (mean ± SE)**	**Controls (mean ± SE)**	**p-value**	**Cases (mean ± SE)**	**Controls (mean ± SE)**	**p-value**
**AAC**	EOPD	21 (804)	8245.1 ± 3440.3	4373.8 ± 378.8	0.276	2240.1 ± 305.9	1580.9 ± 40.8	**0.045***	2.6 ± 0.9	1.7 ± 0.1	0.332	0.003 ± 0.0012	0.002 ± 0.00013	0.276
	LOPD	128 (804)	5091.8 ± 1457.4	4373.8 ± 378.8	0.634	1707.4 ± 115.3	1580.9 ± 40.8	0.302	1.8 ± 0.2	1.7 ± 0.1	0.933	0.002 ± 5E-04	0.002 ± 0.00013	0.634
**AFR**	EOPD	21 (1,631)	25590.9 ± 12908.9	8158.4 ± 297.7	0.192	2510.2 ± 176	2118.8 ± 23.4	**0.039***	7.3 ± 2.4	3.3 ± 0.1	0.109	0.009 ± 0.0045	0.003 ± 1E-04	0.192
	LOPD	137 (1,631)	11624 ± 738.3	8158.4 ± 297.7	**2.22E-05**	2311.9 ± 70.1	2118.8 ± 23.4	**9.82E-03****	4.7 ± 0.3	3.3 ± 0.1	**1.82E-06*****	0.004 ± 0.00026	0.003 ± 1E-04	**2.21E-05*****
**AJ**	EOPD	83 (458)	20847.5 ± 1953.7	21501.1 ± 820	0.758	2854.1 ± 83.2	2826.3 ± 39.7	0.763	7 ± 0.5	7.2 ± 0.2	0.678	0.007 ± 0.00068	0.007 ± 0.00028	0.759
	LOPD	719 (458)	22976.1 ± 1020.6	21501.1 ± 820	0.260	2901.1 ± 32.9	2826.3 ± 39.7	0.147	7.4 ± 0.2	7.2 ± 0.2	0.611	0.008 ± 0.00035	0.007 ± 0.00028	0.260
**AMR**	EOPD	91 (139)	19577 ± 4274.6	13186.3 ± 1351	0.157	2388.5 ± 156.1	2483.4 ± 128.7	0.640	6 ± 0.7	4.6 ± 0.3	0.083	0.007 ± 0.0015	0.005 ± 0.00047	0.157
	LOPD	163 (139)	13160.7 ± 1646.1	13186.3 ± 1351	0.990	2308.9 ± 91.3	2483.4 ± 128.7	0.270	4.8 ± 0.4	4.6 ± 0.3	0.743	0.005 ± 0.00057	0.005 ± 0.00047	0.991
**CAS**	EOPD	53 (267)	9192.6 ± 2856.3	19719.4 ± 2506.5	**6.33E-03**	1567 ± 131.4	2087.3 ± 52.6	**4.62E-04*****	3.7 ± 0.8	6.8 ± 0.7	**0.004****	0.003 ± 0.00099	0.007 ± 0.00087	**0.006****
	LOPD	57 (267)	5803.5 ± 898.6	19719.4 ± 2506.5	**3.16E-07**	1834.3 ± 92	2087.3 ± 52.6	0.019*	2.8 ± 0.4	6.8 ± 0.7	**4.49E-07*****	0.002 ± 0.00031	0.007 ± 0.00087	**3.16E-07*****
**EAS**	EOPD	243 (2,302)	5377.1 ± 518.9	7134.8 ± 197	**1.69E-03**	1777.1 ± 40.8	1854 ± 15	0.078	2.6 ± 0.2	3.4 ± 0.1	**9.38E-04*****	0.002 ± 0.00018	0.003 ± 6.8E-05	**0.002****

	LOPD	455 (2,302)	4759.6 ± 284.7	7134.8 ± 197	**1.24E-11**	1699 ± 37.4	1854 ± 15	**1.31E-04*****	2.3 ± 0.1	3.4 ± 0.1	**4.62E-12*****	0.002 ± 9.9E-05	0.003 ± 6.8E-05	**1.23E-11*****
**EUR**	EOPD	1,042 (7,505)	6727.8 ± 371.5	7053.2 ± 126.2	4.07E-01	1893.3 ± 23.3	1936.8 ± 8.9	0.081	3 ± 0.1	3.2 ± 0	0.202	0.002 ± 0.00013	0.002 ± 4.4E-05	0.407
	LOPD	6,400 (7,505)	6440.6 ± 101.7	7053.2 ± 126.2	**1.58E-04**	1914.9 ± 9.3	1936.8 ± 8.9	0.088	3 ± 0	3.2 ± 0	**0.002****	0.002 ± 3.5E-05	0.002 ± 4.4E-05	**1.58E-04*****
**MDE**	EOPD	31 (20)	54008 ± 11918.8	11143.3 ± 3046	**1.39E-03**	2251.8 ± 106.6	1814.9 ± 206.5	0.070	20.8 ± 4	4.6 ± 1	**3.63E-04*****	0.019 ± 0.0041	0.004 ± 0.0011	**0.001****
	LOPD	128 (20)	23094.8 ± 3162	11143.3 ± 3046	**8.18E-03**	2007.9 ± 72.2	1814.9 ± 206.5	0.386	9.2 ± 1.1	4.6 ± 1	**0.002****	0.008 ± 0.0011	0.004 ± 0.0011	**0.008****
**SAS**	EOPD	22 (198)	11189.9 ± 12058.8	34198.7 ± 4113.8	**4.70E-06**	1996.3 ± 57.8	2155 ± 66	0.074	5.3 ± 1.1	11.8 ± 1.3	**2.2E-04*****	0.004 ± 0.00089	0.0118 ± 0.0014	**4.71E-06*****
	LOPD	40 (198)	15590.6 ± 29727.7	34198.7 ± 4113.8	**3.57E-03**	2160.3 ± 96.1	2155 ± 66	0.964	6.1 ± 1.5	11.8 ± 1.3	**0.006****	0.0054 ± 0.0016	0.0118 ± 0.0014	**0.004****

Legend: AAC = African Admixed, AFR = African, AJ = Ashkenazi Jewish, AMR = American Admixed, AV_{ROH} = average length of runs of homozygosity, CAS = Central Asian, EAS = East Asian, EOPD = early-onset Parkinson's disease (< 45 years), EUR = European, F_{ROH} = runs of homozygosity based estimates for inbreeding coefficient, LOPD = late-onset Parkinson's disease (≥ 45 years), MDE = Middle-Eastern, N_{ROH} = number of runs of homozygosity, SAS = South Asian, SE = standard error, S_{ROH} = total length of runs of homozygosity. p-values indicate the statistical significance as following p < 0.001:***, p < 0.01:**, p < 0.05:*, p ≥ 0.05: not significant

Supplementary Table 13: *Runs of homozygosity in cases subsetted for early-onset and late-onset Parkinson's disease versus controls excluding Parkinson's disease recessive genes (PRKN, PINK1, and DJ-1)*

**Ancestry**	**AAO**	**Number of cases (controls)**	**S_{ROH}**		**AV_{ROH}**		**N_{ROH}**		**F_{ROH}**	
			**Beta (mean ± SE)**	**p-value**	**Beta (mean ± SE)**	**p-value**	**Beta (mean ± SE)**	**p-value**	**Beta (mean ± SE)**	**p-value**

**AAC**	EOPD	21 (804)	2.66E-05 ± 1.63E-05	0.103	6.27E-04 ± 2.05E-04	**0.005****	0.081 ± 0.073	0.077	76.7 ± 47	0.103
	LOPD	128 (804)	1.26E-05 ± 8.02E-06	0.115	1.69E-04 ± 9.06E-05	**0.005****	0.036 ± 0.043	0.077	36.5 ± 23.2	0.115
**AFR**	EOPD	21 (1631)	1.54E-05 ± 7.46E-06	**0.039***	3.45E-04 ± 2.71E-04	**0.019***	0.060 ± 0.028	0.056	44.5 ± 21.5	**0.039***
	LOPD	137 (1631)	-1.35E-05 ± 1.26E-05	0.287	1.23E-06 ± 1.14E-04	**0.019***	-0.024 ± 0.033	0.056	-38.8 ± 36.4	0.287
**AJ**	EOPD	83 (458)	6.71E-06 ± 8E-06	0.401	9.6E-05 ± 1.88E-04	0.336	0.026 ± 0.039	0.828	19.4 ± 23.1	0.401
	LOPD	719 (458)	2.2E-06 ± 2.73E-06	0.420	8.03E-05 ± 7.22E-05	0.336	0.004 ± 0.014	0.828	6.36 ± 7.88	0.420
**AMR**	EOPD	91 (139)	1.19E-05 ± 8.01E-06	0.138	5.73E-05 ± 1.21E-04	0.257	0.067 ± 0.041	0.257	34.3 ± 23.1	0.138
	LOPD	163 (139)	3.29E-06 ± 6.45E-06	0.610	-4.71E-05 ± 9.28E-05	0.257	0.022 ± 0.029	0.257	9.51 ± 18.6	0.610
**CAS**	EOPD	53 (267)	-4.73E-06 ± 7.69E-06	0.539	-3.3E-04 ± 2.11E-04	0.196	-0.016 ± 0.027	0.927	-13.6 ± 22.2	0.539
	LOPD	57 (267)	-3.34E-05 ± 2.43E-05	0.170	-2.9E-05 ± 2.45E-04	0.196	-0.097 ± 0.062	0.927	-96.3 ± 70.1	0.170
**EAS**	EOPD	243 (2302)	4.32E-06 ± 1.3E-05	0.739	3E-05 ± 1.56E-04	0.430	0.015 ± 0.031	0.059	12.5 ± 37.4	0.738
	LOPD	455 (2302)	-2.16E-05 ± 1.21E-05	0.074	-1.96E-04 ± 1.06E-04	0.430	-0.050 ± 0.028	0.059	-62.3 ± 34.8	0.074
**EUR**	EOPD	1042 (7505)	-1.71E-06 ± 3.56E-06	0.631	-9.28E-05 ± 5.12E-05	**0.003****	-0.009 ± 0.013	**1.89E-05*****	-4.93 ± 10.3	0.631
	LOPD	6400 (7505)	-7.96E-06 ± 2.01E-06	**7.57E-05*****	-4.43E-05 ± 2.31E-05	**0.003****	-0.021 ± 0.006	**1.89E-05*****	-23 ± 5.81	**7.58E-05*****

**MDE**	EOPD	31 (20)	3.46E-05 ± 2.6E-05	0.184	-2.72E-04 ± 7.44E-04	0.133	0.096 ± 0.074	**0.042***	99.7 ± 75.1	0.184
	LOPD	128 (20)	3.33E-05 ± 1.69E-05	**0.049***	2.86E-04 ± 3.03E-04	0.133	0.096 ± 0.047	**0.042***	96.1 ± 48.8	**0.049***
**SAS**	EOPD	22 (198)	-2.14E-05 ± 1.25E-05	0.087	-1.84E-04 ± 3.06E-04	0.086	-0.048 ± 0.030	**7.81E-04*****	-61.7 ± 36	0.087
	LOPD	40 (198)	-1.44E-05 ± 7.55E-06	0.057	-4.44E-05 ± 2.5E-04	0.086	-0.041 ± 0.023	**7.81E-04*****	-41.5 ± 21.8	0.057

Legend: AAC = African Admixed, AFR = African, AJ = Ashkenazi Jewish, AMR = American Admixed, AV_{ROH} = average length of runs of homozygosity, CAS = Central Asian, EAS = East Asian, EOPD = early-onset Parkinson's disease (< 45 years), EUR = European, F_{ROH} = runs of homozygosity based-estimates for inbreeding coefficient, LOPD = late-onset Parkinson's disease (≥ 45 years), MDE = Middle-Eastern, N_{ROH} = number of runs of homozygosity, SAS = South Asian, SE = standard error, S_{ROH} = total length of runs of homozygosity. p-values indicate the statistical significance as following p < 0.001:***, p < 0.01:**, p < 0.05:*, p ≥ 0.05: not significant

Supplementary Table 14: *T-test results for runs of homozygosity in cases early-onset Parkinson's disease versus late-onset Parkinson's disease*

Ancestry	Number of EOPD (LOPD)	S_{ROH}			AV_{ROH}			N_{ROH}			F_{ROH}		
		**EOPD (mean ± SE)**	**LOPD (mean ± SE)**	**p-value**	**EOPD (mean ± SE)**	**LOPD (mean ± SE)**	**p-value**	**EOPD (mean ± SE)**	**LOPD (mean ± SE)**	**p-value**	**EOPD (mean ± SE)**	**LOPD (mean ± SE)**	**p-value**
**AAC**	21 (128)	8245.1 ± 3440.3	5091.8 ± 1457.4	0.406	2240.1 ± 305.9	1707.4 ± 115.3	0.115	2.6 ± 0.9	1.8 ± 0.2	0.355	0.0029 ± 0.0012	0.002 ± 5E-04	0.406
**AFR**	21 (137)	25590.9 ± 12908.9	11624 ± 738.3	0.293	2510.2 ± 176	2311.9 ± 70.1	0.304	7.3 ± 2.4	4.7 ± 0.3	0.300	0.0089 ± 0.0045	0.004 ± 3E-04	0.293
**AJ**	83 (719)	20847.5 ± 1953.7	22976.1 ± 1020.6	0.336	2854.1 ± 83.2	2901.1 ± 32.9	0.600	7 ± 0.5	7.4 ± 0.2	0.495	0.0072 ± 7E-04	0.008 ± 4E-04	0.336
**AMR**	91 (163)	19577 ± 4274.6	13160.7 ± 1646.1	0.164	2388.5 ± 156.1	2308.9 ± 91.3	0.661	6 ± 0.7	4.8 ± 0.4	0.144	0.0068 ± 0.0015	0.005 ± 6E-04	0.164
**CAS**	53 (57)	9192.6 ± 2856.3	5803.5 ± 898.6	0.262	1567 ± 131.4	1834.3 ± 92	0.099	3.7 ± 0.8	2.8 ± 0.4	0.320	0.0032 ± 0.001	0.002 ± 3E-04	0.262
**EAS**	243 (455)	5377.1 ± 518.9	4759.6 ± 284.7	0.297	1777.1 ± 40.8	1699 ± 37.4	0.159	2.6 ± 0.2	2.3 ± 0.1	0.205	0.0019 ± 2E-04	0.002 ± 1E-04	0.297
**EUR**	1,042 (6,400)	6727.8 ± 371.5	6440.6 ± 101.7	0.456	1893.3 ± 23.3	1914.9 ± 9.3	0.389	3 ± 0.1	3 ± 0	0.925	0.0023 ± 1E-04	0.002 ± 0	0.456
**MDE**	31 (128)	54008 ± 11918.8	23094.8 ± 3162	**0.017***	2251.8 ± 106.6	2007.9 ± 72.2	0.063	20.8 ± 4	9.2 ± 1.1	**0.008****	0.0187 ± 0.0041	0.008 ± 0.0011	**0.017***
**SAS**	22 (40)	11189.9 ± 2570.9	15590.6 ± 4700.4	0.415	1996.3 ± 57.8	2160.3 ± 96.1	0.149	5.3 ± 1.1	6.1 ± 1.5	0.672	0.0039 ± 9E-04	0.0054 ± 0.0016	0.415

Legend: AAC = African Admixed, AFR = African, AJ = Ashkenazi Jewish, AMR = American Admixed, AV_{ROH} = average length of runs of homozygosity, CAS = Central Asian, EAS = East Asian, EOPD = early-onset Parkinson's disease (< 45 years), EUR = European, F_{ROH} = runs of homozygosity-based estimates for inbreeding coefficient, LOPD = late-onset Parkinson's disease (≥ 45 years), MDE = Middle-Eastern, N_{ROH} = number of runs of homozygosity, SAS = South Asian, SE = standard error, S_{ROH} = total length of runs of homozygosity. *p*-values indicate the statistical significance as following $p < 0.001$:***, $p < 0.01$:**, $p < 0.05$:*, $p \geq 0.05$: not significant

Supplementary Table 15: *Runs of homozygosity only in cases early-onset versus late-onset Parkinson's disease excluding Parkinson's disease recessive genes (PRKN, PINK1, and DJ-1)*

**Ancestry**	**Number of EOPD (LOPD)**	S_{ROH}		AV_{ROH}		N_{ROH}		F_{ROH}	
		**Beta (mean ± SE)**	**p-value**	**Beta (mean ± SE)**	**p-value**	**Beta (mean ± SE)**	**p-value**	**Beta (mean ± SE)**	**p-value**
**AAC**	21 (128)	-3.88E-05 ± 1.9E-05	**0.041***	-8.61E-04 ± 3.6E-04	**0.017***	-0.296 ± 0.138	**0.032***	-112 ± 54.7	**0.041***
**AFR**	21 (137)	-4.31E-04 ± 2.42E-04	0.075	1.72E-03 ± 1.06E-03	0.105	-0.557 ± 0.364	0.127	-1240 ± 697	0.075
**AJ**	83 (719)	5.95E-07 ± 4.97E-06	0.905	9.73E-06 ± 1.85E-04	0.958	-0.003 ± 0.028	0.909	1.71 ± 14.3	0.905
**AMR**	91 (163)	-9.41E-06 ± 5.55E-06	0.090	-2.22E-05 ± 1.42E-04	0.875	-0.051 ± 0.028	0.073	-27.1 ± 16	0.090
**CAS**	53 (57)	-2.25E-05 ± 2.54E-05	0.376	5.28E-04 ± 4.47E-04	0.237	-0.075 ± 0.079	0.346	-64.9 ± 73.2	0.376
**EAS**	243 (455)	-2.52E-05 ± 1.76E-05	0.151	-1.58E-04 ± 1.39E-04	0.257	-0.068 ± 0.045	0.129	-72.8 ± 50.8	0.151
**EUR**	1042 (6400)	-8.32E-06 ± 4.37E-06	0.057	-7.86E-06 ± 5.92E-05	0.894	-0.016 ± 0.018	0.266	-24 ± 12.6	0.057
**MDE**	31 (128)	1.47E-06 ± 6.7E-06	0.826	3.19E-04 ± 5.1E-04	0.532	0.006 ± 0.021	0.764	4.25 ± 19.3	0.826
**SAS**	22 (40)	-7.97E-06 ± 4.15E-05	0.848	-2E-04 ± 9.99E-04	0.841	-0.052 ± 0.105	0.621	-23 ± 120	0.848

Legend: AAC = African Admixed, AFR = African, AJ = Ashkenazi Jewish, AMR = American Admixed, AV_{ROH} = average length of runs of homozygosity, CAS = Central Asian, EAS = East Asian, EOPD = early-onset Parkinson's disease (< 45 years), EUR = European, F_{ROH} = runs of homozygosity-based estimates for inbreeding coefficient, LOPD = late-onset Parkinson's disease (≥ 45 years), MDE = Middle-Eastern, N_{ROH} = number of runs of homozygosity, SAS = South Asian, SE= standard error, S_{ROH} = total length of runs of homozygosity. p-values indicate the statistical significance as following $p < 0.001$:***, $p < 0.01$:**, $p < 0.05$:*, $p \geq 0.05$: not significant

Supplementary Table 16: *Average runs of homozygosity (2Mb to 10Mb) across ancestry groups*

		>2Mb	>3Mb	>4Mb	>5Mb	>6Mb	>7Mb	>8Mb	>9Mb	>10Mb
**AAC**	**Cases (mean ± SE)**	1392.9 ± 114.6	1045.8 ± 141.9	882.9 ± 151.5	772.3 ± 174.7	663.7 ± 176.6	592.7 ± 181	553.1 ± 181.1	579.0 ± 188.5	548.2 ± 187.6
	**Controls (mean ± SE)**	1090.2 ± 155.4	634.9 ± 1797.3	515.4 ± 67.2	427.7 ±66.4	396.2 ± 68.8	350.2 ± 67.6	279.9 ± 65.3	244.5 ± 64	220.2 ± 64.3
	**p-value**	**0.016 ***	**0.005 ***	**0.009 ***	**0.017 ***	0.078	0.136	0.120	0.070	0.059
**AFR**	**Cases (mean ± SE)**	2052.5 ± 50.2	1494.8 ± 71	988.9 ± 72.4	672.1 ± 68.5	447 ± 61.3	272.3 ± 52.8	193 ± 47.1	168.7 ± 45.4	106.4 ± 37.8
	**Controls (mean ± SE)**	1676.2 ± 40	1197.8 ± 50.3	814.5 ± 50.5	560.2 ± 47.5	380.5 ± 44.2	286.8 ± 42.2	215.2 ± 39	172.9 ± 36.7	146.6 ± 35.2
	**p-value**	0.004	0.105	0.345	0.405	0.638	0.859	0.950	0.712	0.787
**AJ**	**Cases (mean ± SE)**	3610.5 ± 44.9	4262.4 ± 76	4239.9 ± 104.7	3766.3 ± 125.1	3085.9 ± 135.3	2445.4 ± 137.4	1924.5 ± 134	1541.7 ± 130.2	1167.9 ± 121.9
	**Controls (mean ± SE)**	3486.7 ± 64.4	4110.4 ± 105.4	3916.2 ± 148.5	3435.8 ± 177	2841 ± 196.2	2198.3 ± 197.2	1702.5 ± 191	1434.1 ± 183.4	1090.3 ± 169.2
	**p-value**	0.170	0.328	0.115	0.178	0.444	0.476	0.504	0.853	0.871
**AMR**	**Cases (mean ± SE)**	2480.7 ± 116.7	1990.1 ± 156.1	1762.2 ± 174.4	1600.8 ± 184.9	1443.9 ± 193.1	1421.9 ± 207.4	1314.6 ± 211.1	1287.3 ± 218.9	1214.8 ± 219.3
	**Controls (mean ± SE)**	2827.3 ± 304	2584.1 ± 355.4	2260.6 ± 370.4	2240.3 ± 383.5	2108.6 ± 401.4	1892.9 ± 405.6	1705.9 ± 401.9	1509.9 ± 401	1349 ± 398.8
	**p-value**	0.294	0.117	0.204	0.130	0.142	0.358	0.509	0.819	0.977
**CAS**	**Cases (mean ± SE)**	1720.6 ± 98.8	1251.4 ± 131.3	1060 ± 149.2	862.2 ± 152.7	819.8 ± 156.2	829 ± 172.1	644.5 ± 161.4	500.8 ± 151.9	446.7 ± 149.1
	**Controls (mean ± SE)**	1992.7 ± 95.6	1487.7 ± 140.3	1350.5 ± 158.1	1273.3 ± 170	1168 ± 182.4	1104.7 ± 186.5	974.6 ± 188.8	845.7 ± 184.2	801.6 ± 184.2
	**p-value**	0.811	0.360	0.157	0.301	0.287	0.127	0.331	0.585	0.831

EAS	Cases (mean ± SE)	1313.9 ± 36.3	583.2 ± 37.2	292.8 ± 31.2	152.6 ± 25.3	88 ± 20.8	56.5 ± 18	37.1 ± 15.2	20.3 ± 11.7	14.5 ± 10.3
	Controls (mean ± SE)	1601.4 ± 29.5	853.6 ± 34.4	451.3 ± 30.6	206 ± 23.8	109.6 ± 19.1	59.1 ± 15	33.3 ± 12	14.5 ± 8.5	6.2 ± 6.2
	**p-value**	0.084	0.228	0.185	0.228	0.409	0.638	0.720	0.525	0.938
EUR	Cases (mean ± SE)	1529.8 ± 14.5	788.2 ± 16.6	447.2 ± 15.3	303 ± 14.2	228.7 ± 13.5	181.7 ± 12.8	153 ± 12.3	127.4 ± 11.6	107 ± 11.1
	Controls (mean ± SE)	1610.2 ± 18.7	856.2 ± 21.8	553.1 ± 21.4	420.2 ± 20.9	342.3 ± 20.6	292.9 ± 20.6	250.4 ± 20.1	223.8 ± 19.7	191.6 ± 19.2
	**p-value**	**8.5E-5 *** **	**0.0046** **	**2.5E-5 *** **	**4.00E-6 *** **	**4.00E-6 *** **	**6.00E-6 *** **	**5.6E-5 *** **	**3.8E-5 *** **	**1.57E-4 *** **
MDE	Cases (mean ± SE)	2045.1 ± 95.2	1836.8 ± 144.5	1685.2 ± 176.8	1346.6 ± 185	1060.5 ± 182.4	655.5 ± 159.5	530.1 ± 150.5	258.1 ± 115.1	211.7 ± 105.8
	Controls (mean ± SE)	1637.4 ± 360.9	1670.3 ± 672.3	2059.4 ± 1219.3	1845.7 ± 1217.6	1570.8 ± 1205.2	1570.8 ± 1205.2	1570.8 ± 1205.2	1152.5 ± 1152.5	1152.5 ± 1152.5
	**p-value**	0.165	0.219	0.825	0.875	0.730	0.348	0.252	0.124	0.114
SAS	Cases (mean ± SE)	1865.7 ± 136	1336.9 ± 180.1	939.2 ± 189.7	642.7 ± 179.2	475.2 ± 180.8	380.8 ± 171.7	254.6 ± 148.8	191.3 ± 136	108.9 ± 108.9
	Controls (mean ± SE)	2148.9 ± 115.4	1969 ± 177.5	2018.5 ± 209.1	2023.4 ± 240.2	1788.3 ± 250.1	1778.5 ± 264	1657 ± 272.3	1499.6 ± 272.5	1073.8 ± 252.4
	**p-value**	0.059	**0.013 ** **	**0.00047 *** **	**8.0E-5 *** **	**3.82E-4 *** **	**5.24E-4 *** **	**9.81E-4 *** **	**0.003 ** **	**0.021 * **

Legend: AAC = African Admixed, AFR = African, AJ = Ashkenazi Jewish, AMR = American Admixed, CAS = Central Asian, EAS = East Asian, EUR = European, Mb = megabases, MDE = Middle-Eastern, SAS = South Asian, SE = standard error. *p*-values indicate the statistical significance as following $p < 0.001$:***, $p < 0.01$:**, $p < 0.05$:*, $p \geq 0.05$: not significant. The Bonferroni threshold was adjusted by dividing 0.05 by the number of ranges to account for multiple comparisons.

Supplementary Table 17: *Average runs of homozygosity (2Mb to 10Mb) for early-onset and late-onset Parkinson's disease across ancestry groups*

Ancestry	AAO	>2Mb		>3Mb		>4Mb		>5Mb		>6Mb		>7Mb		>8Mb		>9Mb		>10Mb	
		EOPD	LOPD	EOPD	LOPD	EOPD	LOPD	EOPD	LOPD	EOPD	LOPD	EOPD	LOPD	EOPD	LOPD	EOPD	LOPD	EOPD	LOPD

**AAC**	**Number of cases (controls)**	21 (804)	128 (804)	21 (804)	128 (804)	21 (804)	128 (804)	21 (804)	128 (804)	21 (804)	128 (804)	21 (804)	128 (804)	21 (804)	128 (804)	21 (804)	128 (804)	21 (804)	128 (804)
	**Cases (mean ± SE)**	2000.4 ± 400.3	1349.6 ± 160	1561.9 ± 523.3	916 ± 184.3	1295.2 ± 531.2	659.7 ± 179.8	960.6 ± 533.6	474.5 ± 173.3	764 ± 530.7	382.9 ± 176	776.5 ± 538.2	275.7 ± 160.5	463.9 ± 463.9	275.7 ± 160.5	546.1 ± 546.1	281.5 ± 164.6	546.1 ± 546.1	219.9 ± 159.6
	**Controls (mean ± SE)**	1090.2 ± 55.4	1090.2 ± 55.4	634.9 ± 63.3	634.9 ± 63.3	515.4 ± 67.2	515.4 ± 67.2	427.7 ± 66.4	427.7 ± 66.4	396.2 ± 68.8	396.2 ± 68.8	350.2 ± 67.6	350.2 ± 67.6	279.9 ± 65.3	279.9 ± 65.3	244.5 ± 64	244.5 ± 64	220.2 ± 64.3	220.2 ± 64.3
	**p-value**	**0.005 ** **	0.052	**0.003 ** **	**0.039 * **	**0.017 * **	**0.040 * **	0.063	0.082	0.115	0.191	0.094	0.385	0.175	0.296	0.109	0.235	0.116	0.356
**AFR**	**Number of cases (controls)**	21 (1631)	137 (1631)	21 (1631)	137 (1631)	21 (1631)	137 (1631)	21 (1631)	137 (1631)	21 (1631)	137 (1631)	21 (1631)	137 (1631)	21 (1631)	137 (1631)	21 (1631)	137 (1631)	21 (1631)	137 (1631)
	**Cases (mean ± SE)**	2455.3 ± 345.5	2251.8 ± 119.4	2776.9 ± 548.2	2046.7 ± 182.9	2181.5 ± 650.9	1288.2 ± 196	1594.2 ± 664.7	824.7 ± 178.2	1137.1 ± 645	399.1 ± 137.8	559.6 ± 559.6	163.2 ± 93.5	620.9 ± 620.9	0 ± 0	713.3 ± 713.3	0 ± 0	713.3 ± 713.3	0 ± 0
	**Controls (mean ± SE)**	1676.2 ± 40	1676.2 ± 40	1197.8 ± 50.3	1197.8 ± 50.3	814.5 ± 50.5	814.5 ± 50.5	560.2 ± 47.5	560.2 ± 47.	380.5 ± 44.2	380.5 ± 44.2	286.8 ± 42.2	286.8 ± 42.2	215.2 ± 39	215.2 ± 39	172.9 ± 36.7	172.9 ± 36.7	146.6 ± 35.2	146.6 ± 35.2
	**p-value**	0.387	0.905	0.079	0.515	0.149	0.115	0.273	0.052	0.282	0.080	0.656	0.051	0.177	0.978	0.111	0.981	0.101	0.976
**AJ**	**Number of cases (controls)**	83 (458)	719 (458)	83 (458)	719 (458)	83 (458)	719 (458)	83 (458)	719 (458)	83 (458)	719 (458)	83 (458)	719 (458)	83 (458)	719 (458)	83 (458)	719 (458)	83 (458)	719 (458)

	**Cases (mean ± SE)**	3591.6 ± 137.1	3645.2 ± 52.8	4204.2 ± 250.9	4344.7 ± 88.8	4171.8 ± 373.4	4289.7 ± 122	3567.1 ± 446.1	3816.5 ± 146.8	2913.4 ± 462.5	3082.3 ± 161.1	1900.1 ± 440.6	2463.7 ± 164.2	1451.1 ± 414.7	1907 ± 159.8	1365.6 ± 410.4	1526.7 ± 155.7	1268.8 ± 403.7	1187.6 ± 147.7
	**Controls (mean ± SE)**	3486.7 ± 64.4	3486.7 ± 64.4	4110.4 ± 105.4	4110.4 ± 105.4	3916.2 ± 148.5	3916.2 ± 148.5	3435.8 ± 177	3435.8 ± 177	2841 ± 196.2	2841 ± 196.2	2198.3 ± 197.2	2198.3 ± 197.2	1702.5 ± 191	1702.5 ± 191	1434.1 ± 183.4	1434.1 ± 183.4	1090.3 ± 169.2	1090.3 ± 169.2
	**p-value**	0.757	0.132	0.748	0.162	0.512	0.112	0.547	0.175	0.786	0.651	0.895	0.637	0.792	0.778	0.343	0.795	0.321	0.843
**AMR**	**Number of cases (controls)**	91 (139)	163 (139)	91 (139)	163 (139)	91 (139)	163 (139)	91 (139)	163 (139)	91 (139)	163 (139)	91 (139)	163 (139)	91 (139)	163 (139)	91 (139)	163 (139)	91 (139)	163 (139)
	**Cases (mean ± SE)**	2640.3 ± 259	2566.6 ± 176.7	2222.5 ± 360.5	1887.7 ± 226.9	2177.7 ± 386.3	1553.6 ± 255.9	1906.8 ± 399.3	1451.2 ± 275	1930.5 ± 417	1285.7 ± 287.6	1611.7 ± 411.4	1379.8 ± 330	1390.3 ± 429	1371 ± 335.5	1462.2 ± 478.1	1263.3 ± 338.3	1538.3 ± 500.6	1207.3 ± 334.8
	**Controls (mean ± SE)**	2827.3 ± 304	2827.3 ± 304	2584.1 ± 355.4	2584.1 ± 355.4	2260.6 ± 370.4	2260.6 ± 370.4	2240.3 ± 383.5	2240.3 ± 383.5	2108.6 ± 401.4	2108.6 ± 401.4	1892.9 ± 405.6	1892.9 ± 405.6	1705.9 ± 401.9	1705.9 ± 401.9	1509.9 ± 401	1509.9 ± 401	1349 ± 398.8	1349 ± 398.8
	**p-value**	0.626	0.829	0.764	0.341	0.437	0.318	0.524	0.240	0.266	0.216	0.528	0.517	0.537	0.758	0.377	0.904	0.324	0.955
**CAS**	**Number of cases (controls)**	53 (267)	57 (267)	53 (267)	57 (267)	53 (267)	57 (267)	53 (267)	57 (267)	53 (267)	57 (267)	53 (267)	57 (267)	53 (267)	57 (267)	53 (267)	57 (267)	53 (267)	57 (267)
	**Cases (mean ± SE)**	1433.7 ± 237.9	1356.4 ±	749.1 ± 264.6	704.8 ± 193.9	624.3 ± 273.5	240.6 ± 137.2	596.2 ± 290.5	92 ± 92	621.4 ± 301.9	0 ± 0	688.5 ± 338.7	0 ± 0	556.1 ± 317.7	0 ± 0	412.9 ± 289.5	0 ± 0	435.4 ± 304.9	0 ± 0

			186.3																
	**Controls (mean ± SE)**	1992.7 ± 95.6	1992.7 ± 95.6	1487.7 ± 140.3	1487.7 ± 140.3	1350.5 ± 158.1	1350.5 ± 158.1	1273.3 ± 170	1273.3 ± 170	1168 ± 182.4	1168 ± 182.4	1104.7 ± 186.5	1104.7 ± 186.5	974.6 ± 188.8	974.6 ± 188.8	845.7 ± 184.2	845.7 ± 184.2	801.6 ± 184.2	801.6 ± 184.2
	**p-value**	0.396	0.335	0.214	0.733	0.522	0.261	0.648	0.304	0.854	0.990	0.833	0.986	0.610	0.988	0.800	0.990	0.693	0.986
**EAS**	**Number of cases (controls)**	243 (2302)	455 (2302)	243 (2302)	455 (2302)	243 (2302)	455 (2302)	243 (2302)	455 (2302)	243 (2302)	455 (2302)	243 (2302)	455 (2302)	243 (2302)	455 (2302)	243 (2302)	455 (2302)	243 (2302)	455 (2302)
	**Cases (mean ± SE)**	1212.8 ± 85.2	1249.1 ± 67.9	421.7 ± 79.8	581.4 ± 68.9	239.9 ± 68.9	283.9 ± 57.6	77.3 ± 45.7	172 ± 50.1	38 ± 38	113.4 ± 43.1	43.1 ± 43.1	70.7 ± 35.5	43.1 ± 43.1	21.5 ± 21.5	43.1 ± 43.1	21.5 ± 21.5	47 ± 47	0 ± 0
	**Controls (mean ± SE)**	1601.4 ± 29.5	1601.4 ± 29.5	853.6 ± 34.4	853.6 ± 34.4	451.3 ± 30.6	451.3 ± 30.6	206 ± 23.8	206 ± 23.8	109.6 ± 19.1	109.6 ± 19.1	59.1 ± 15	59.1 ± 15	33.3 ± 12	33.3 ± 12	14.5 ± 8.5	14.5 ± 8.5	6.2 ± 6.2	6.2 ± 6.2
	**p-value**	0.155	**0.033 ***	0.581	0.373	0.721	0.545	0.648	0.838	0.525	0.427	0.848	0.464	0.861	0.646	0.862	0.657	0.569	0.990
**EUR**	**Number of cases (controls)**	1042 (7505)	6400 (7505)	1042 (7505)	6400 (7505)	1042 (7505)	6400 (7505)	1042 (7505)	6400 (7505)	1042 (7505)	6400 (7505)	1042 (7505)	6400 (7505)	1042 (7505)	6400 (7505)	1042 (7505)	6400 (7505)	1042 (7505)	6400 (7505)
	**Cases (mean ± SE)**	1497 ± 50.1	1542.6 ± 19.9	815.1 ± 58.9	789.6 ± 22.6	500.7 ± 56.8	458.9 ± 21.1	359.2 ± 56.1	315.4 ± 19.6	296 ± 55	245.8 ± 18.8	266.5 ± 54.6	200.2 ± 18	266.1 ± 56.3	166.9 ± 17.5	254.3 ± 56.4	136.2 ± 16.4	223.5 ± 54.8	116.7 ± 15.8
	**Controls (mean ± SE)**	1610.2 ± 18.7	1610.2 ± 18.7	856.2 ± 21.8	856.2 ± 21.8	553.1 ± 21.4	553.1 ± 21.4	420.2 ± 20.9	420.2 ± 20.9	342.3 ± 20.6	342.3 ± 20.6	292.9 ± 20.6	292.9 ± 20.6	250.4 ± 20.1	250.4 ± 20.1	223.8 ± 19.7	223.8 ± 19.7	191.6 ± 19.2	191.6 ± 19.2

	**p-value**	**0.04 ***	**0.010 ***	0.064	**0.042 ***	0.336	**0.001 ****	0.329	**4.08E-4 *****	0.807	**3.73E-4 *****	0.879	**4.35E-4*****	0.380	**0.001 ****	0.156	**3.68E-4 *****	0.122	**0.002 ****
**MDE**	**Number of cases (controls)**	214 (20)	214 (20)	214 (20)	214 (20)	214 (20)	214 (20)	214 (20)	214 (20)	214 (20)	214 (20)	214 (20)	214 (20)	214 (20)	214 (20)	214 (20)	214(20)	214(20)	214 (20)
	**Cases (mean ± SE)**	2498.4 ± 218	2003.7 ± 126.9	2762.6 ± 381.4	1732 ± 187.1	3057.4 ± 551.5	1618.9 ± 221.7	2582.3 ± 618	1273.5 ± 233	2052.3 ± 654.2	999.2 ± 228.4	1274.4 ± 610.4	664.7 ± 203.8	1305.7 ± 628.2	506.6 ± 187.9	790.8 ± 553	240 ± 137.5	790.8 ± 553	162.3 ± 114.4
	**Controls (mean ± SE)**	1637.4 ± 360.9	1637.4 ± 360.9	1670.3 ± 672.3	1670.3 ± 672.3	2059.4 ± 1219.3	2059.4 ± 1219.3	1845.7 ± 1217.6	1845.7 ± 1217.6	1570.8 ± 1205.2	1570.8 ± 1205.2	1570.8 ± 1205.2	1570.8 ± 1205.2	1570.8 ± 1205.2	1570.8 ± 1205.2	1152.5 ± 1152.5	1152.5 ± 1152.5	1152.5 ± 1152.5	1152.5 ± 1152.5
	**p-value**	0.893	0.212	0.705	0.420	0.726	0.944	0.566	0.986	0.687	0.840	0.722	0.406	0.722	0.322	0.804	0.213	0.804	0.203
**SAS**	**Number of cases (controls)**	22 (198)	40 (198)	22 (198)	40 (198)	22 (198)	40 (198)	22 (198)	40 (198)	22 (198)	40 (198)	22 (198)	40 (198)	22 (198)	40 (198)	22 (198)	40 (198)	22 (198)	40 (198)
	**Cases (mean ± SE)**	2063.5 ± 251.5	2168.8 ± 221.9	981.5 ± 353	1680.1 ± 319.6	424.5 ± 293.2	1101.5 ± 360.4	0 ± 0	838.2 ± 361.8	0 ± 0	858.5 ± 430.9	0 ± 0	736.6 ± 424.7	0 ± 0	543.9 ± 385.5	0 ± 0	559.7 ± 394.6	0 ± 0	318.7 ± 318.7
	**Controls (mean ± SE)**	2148.9 ± 115.4	2148.9 ± 115.4	1969 ± 177.5	1969 ± 177.5	2018.5 ± 209.1	2018.5 ± 209.1	2023.4 ± 240.2	2023.4 ± 240.2	1788.3 ± 250.1	1788.3 ± 250.1	1778.5 ± 264	1778.5 ± 264	1657 ± 272.3	1657 ± 272.3	1499.6 ± 272.5	1499.6 ± 272.5	1073.8 ± 252.4	1073.8 ± 252.4
	**p-value**	0.737	0.605	0.117	0.741	**0.030 ***	0.081	0.986	**0.024 ***	0.980	0.083	0.980	0.075	0.980	0.053	0.982	0.083	0.991	0.217

Legend: AAC = African Admixed, AFR = African, AJ = Ashkenazi Jewish, AMR = American Admixed, CAS = Central Asian, EAS = East Asian, EOPD = early-onset PD, EUR = European, LOPD = late-onset PD, Mb = megabases, MDE = Middle-Eastern, SAS = South Asian, SE = standard error. p-values indicate the statistical significance as following p < 0.001: ***, p < 0.01:**, p < 0.05:*, p ≥ 0.05: not significant. The Bonferroni threshold was adjusted by dividing 0.05 by the number of ranges to account for multiple comparisons.

Supplementary Table 18: *Homozygosity mapping results for runs of homozygosity enriched in individuals with consanguinity and early-onset Parkinson's disease.*

ROH pools enriched in individuals with consanguinity										
**Ancestry**	**Cases with consanguinity (Percentage)**	**Age at onset (mean ± SD)**	**Has family history (Percentage)**	**ROHs in cases only for individuals with consanguinity**	**Total ROH overlaps**	**Number of ROH enriched in cases**	**Number of ROH enriched in cases that pass Bonferroni**	**Total number of ROH overlapping PD genes**	**Number of ROH overlapping PD genes enriched in individuals with consanguinity**	**Number of ROHs overlapping PD genes that pass Bonferroni**
**AAC**	6 (2.3)	55 ± 24	16.7	3	405	122	51	45	18	9
**AFR**	13 (1.4)	29 ± NA	0	1	1,162	536	128	125	68	19
**AJ**	49 (4.9)	64.5 ± 10	26.5	10	1,217	1,059	56	122	112	15
**AMR**	20 (5.5)	42.9 ± 17	20	86	406	350	7	46	38	2
**CAS**	15 (5.7)	38 ± 4.2	33.3	17	861	575	66	93	68	13
**EAS**	10 (0.7)	42.6 ± 20	10	2	1047	412	118	100	38	14
**EUR**	75 (0.6)	57.1 ± 16	33.3	1	1,648	1,490	851	145	130	93

**MDE**	42 (19.6)	46.4 ± 13	40.5	298	483	454	0	68	63	0
**SAS**	5 (4.3)	51.3 ± 8.4	20	2	587	276	6	67	30	2
**ROH pools enriched in EOPD cases**										
**Ancestry**	**EOPD Cases (Percentage)**	**Age at onset (mean ± SD)**	**Has family history (Percentage)**	**ROHs in cases only for EOPD cases**	**Total ROH overlaps**	**Number of ROH enriched in EOPD cases**	**Number of ROH that pass Bonferroni**	**Total number of ROH overlapping PD genes**	**Number of ROH overlapping PD genes enriched in EOPD cases**	**Number of ROHs overlapping PD genes that pass Bonferroni**
**AAC**	21 (8.2)	37.6 ± 4.9	0.485	0	374	53	0	40	3	0
**AFR**	21 (2.3)	35.7 ± 8.3	0.242	0	1,117	286	16	117	34	0
**AJ**	83 (8.3)	39.1 ± 6.1	7.02	3	1,091	486	0	107	54	0
**AMR**	91 (25.1)	35.7 ± 7.6	7.39	60	345	228	0	42	33	0
**CAS**	53 (20)	35.9 ± 6.3	1.25	0	685	159	0	77	25	0
**EAS**	243 (15.8)	37.2 ± 6.9	1.85	0	1034	276	0	25	2	0
**EUR**	1042 (8.9)	37.5 ± 6.5	4.47	3	1,547	669	1	136	65	0
**MDE**	31 (14.5)	35.2 ± 7.9	17.6	175	251	205	0	33	30	0
**SAS**	22 (18.8)	37 ± 4.4	1.82	0	713	104	0	84	13	0

Legend: AAC = African Admixed, AFR = African, AJ = Ashkenazi Jewish, AMR = American Admixed, CAS = Central Asian, EAS = East Asian, EOPD = early-onset Parkinson's disease (< 45 years), EUR = European, LOPD = late-onset Parkinson's disease (≥ 45 years), MDE = Middle-Eastern, PD = Parkinson's disease, ROHs = runs of homozygosity, SAS = South Asian. The Bonferroni threshold was adjusted by dividing 0.05 by the number of enriched ROHs to account for multiple comparisons.

Supplementary Table 19: *Annotated variants only present in Parkinson's disease cases prioritized from homozygosity mapping with additional annotation information*

Ancestry	ROH Pool	Number of cases with ROH pool	Present in multiple cases	CHR:BP:REF:ALT	rsID	Gene	Variant type	Variant class	Variant Impact	Clinical significance	REVEL score	AlphaMissense class	Eve class
AMR	S129	4	Yes	4:130964119:G:T	rs78589926	*TIGD1*	Splice donor variant & Non-coding transcript variant	SNV	High	Not reported	Not reported	Not reported	Not reported
MDE	S164 and S186	11 and 10	Yes	1:20649109:C:T	rs45539432&CM052340	*PINK1*	Stop gained	SNV	High	Pathogenic	Not reported	Not reported	Not reported

MDE	S419	8	Yes	14:66924264:A:G	rs41285470	*GPHN*	Missense variant	SNV	Moderate	Not reported	Not reported	Not reported	Not reported
MDE	S419	8	Yes	14:60118341:C:T	rs773266496&COSV58303323	*PCNX4*	Missense variant	SNV	Moderate	Not reported	0.130	Likely benign	Not reported
MDE	S419	8	Yes	14:67577308:C:T	rs369145807	*PLEKHH1*	Missense variant	SNV	Moderate	Not reported	0.068	Not reported	Not reported
MDE	S419	8	Yes	14:69352130:G:A	rs144512254	*GALNT16*	Missense variant	SNV	Moderate	Not reported	0.062	Likely benign	Not reported
MDE	S419	8	Yes	14:72940346:G:A	rs145062084&COSV105912372	*DCAF4*	Missense variant	SNV	Moderate	Likely benign	0.017	Likely benign	Not reported
MDE	S419	8	Yes	14:105863242:T:C	rs2338627	*IGHJ6*	Missense variant	SNV	Moderate	Not reported	Not reported	Not reported	Not reported
MDE	S419	8	Yes	14:105863243:A:C	rs2338626	*IGHJ6*	Missense variant	SNV	Moderate	Not reported	Not reported	Not reported	Not reported
AAC	S397	2	No	6:42188686:C:T	rs137853903&CM045052&COSV57835221	*GUCA1B*	Missense	SNV	Moderate	Benign & likely benign	0.239	Not reported	Ambiguous

							variant						
AAC	S397	2	No	6:42745604:C:T	rs7742995	*TBCC*	Missense variant	SNV	Moderate	Not reported	0.042	Not reported	Likely benign
AAC	S397	2	No	6:42978919:G:A	rs1420377361	*PEX6*	Missense variant	SNV	Moderate	Not reported	0.388	Not reported	Not reported
AAC	S396	2	No	6:42188686:C:T	rs137853903&CM045052&COSV57835221	*GUCA1B*	Missense variant	SNV	Moderate	Benign & likely benign	0.239	Not reported	ambiguous
AJ	S1665	3	No	13:23340893:C:A	rs142967124	*SACS*	Missense variant	SNV	Moderate	Benign & likely benign	0.122	Benign	Likely benign
AJ	S1651	1	No	11:8985795:G:A	rs61756060&COSV58469013	*NRIP3*	Missense variant	SNV	Moderate	Not reported	0.219	Not reported	Likely benign
AJ	S1650	3	No	11:551741:C:T	rs139348192	*LRRC56*	Missense variant	SNV	Moderate	Benign	0.101	Pathogenic	Likely benign
AJ	S1623	3	No	9:2823786:C:T	rs775393848	*PUM3*	Missense variant	SNV	Moderate	Not reported	0.517	Not reported	Likely benign

AJ	S1443	4	No	8:120255342:T:C	rs141272095	*COL14A1*	Missense variant	SNV	Moderate	Not reported	0.246	Not reported	Likely benign
AMR	S82	5	No	9:84723600:C:A	rs139913267&COSV52853311&COSV52860967	*NTRK2*	Missense variant	SNV	Moderate	Benign/likely benign	Not reported	Not reported	Not reported
MDE	S606	1	No	15:78766976:C:T	rs142824118&COSV66308197	*ADAMTS7*	Missense variant	SNV	Moderate	Not reported	0.035	Not reported	Likely benign
SAS	S892	2	No	8:128916918:T:A	rs369143754	*CCDC26*	Splice acceptor variant & Non-coding transcript variant	SNV	High	Not reported	Not reported	Not reported	Not reported

Legend: AAC = African Admixed, AJ = Ashkenazi Jewish, ALT = alternative allele, AMR = American Admixed, CADD = Combined Annotation Dependent Depletion, Eve class = Evolutionary model of Variant Effect, GnomAD = Genome Aggregation Database, MDE = Middle Eastern, miRNA = micro non-coding RNA, REF = reference allele, REVEL = Rare Exome Variant Ensemble Learner, ROH = runs of homozygosity, SIFT = Sorting Intolerant From Tolerant, SAS = South Asian, SNV = single nucleotide variant

Supplementary Table 20: *Runs of homozygosity pools enriched in individuals with consanguinity that pass Bonferroni correction*

Ancestry	POOL	Total participants	Total cases	Total controls	CHR	Base position 1	Base position 2	ROH KB	NSNP	p-value	Proportion cases	Proportion controls	Bonferroni correction
AAC	S51	10	2	8	16	47412386	47986415	574.03	452	1.21E-07	3.33E-01	9.95E-03	9.95E-10
	S60	9	2	7	4	33806951	34461178	654.228	852	2.10E-08	3.33E-01	8.71E-03	1.73E-10
	S109	6	2	4	15	50538139	51386390	848.252	2004	3.50E-12	3.33E-01	4.98E-03	2.87E-14
	S110	6	2	4	15	51387273	52073368	686.096	1125	3.50E-12	3.33E-01	4.98E-03	2.87E-14
	S112	5	2	3	1	196205159	197607410	1402.252	1883	1.95E-14	3.33E-01	3.73E-03	1.60E-16
	S121	5	2	3	4	95890460	96650758	760.299	1379	1.95E-14	3.33E-01	3.73E-03	1.60E-16
	S122	5	2	3	4	96671601	97127636	456.036	1056	1.95E-14	3.33E-01	3.73E-03	1.60E-16
	S175	4	2	2	7	26445327	27398076	952.75	2004	8.32E-18	3.33E-01	2.49E-03	6.82E-20
	S206	4	2	2	16	49010331	49131107	120.777	345	8.32E-18	3.33E-01	2.49E-03	6.82E-20
	S223	3	2	1	1	197960367	199306182	1345.816	2558	2.09E-23	3.33E-01	1.24E-03	1.71E-25
	S279	3	2	1	8	15150815	15503777	352.963	1427	2.09E-23	3.33E-01	1.24E-03	1.71E-25
	S288	3	2	1	9	79594520	81396355	1801.836	4538	2.09E-23	3.33E-01	1.24E-03	1.71E-25
	S293	3	2	1	11	6685032	7879993	1194.962	3614	2.09E-23	3.33E-01	1.24E-03	1.71E-25

	S295	3	2	1	11	30628734	32256825	1628.092	2981	2.09E-23	3.33E-01	1.24E-03	1.71E-25
	S312	3	2	1	15	52714993	52954752	239.76	719	2.09E-23	3.33E-01	1.24E-03	1.71E-25
	S313	3	2	1	15	53561105	53700150	139.046	351	2.09E-23	3.33E-01	1.24E-03	1.71E-25
	S329	2	1	1	1	13621474	15419749	1798.276	4443	6.18E-05	1.67E-01	1.24E-03	5.06E-07
	S339	2	1	1	1	218218586	219625675	1407.09	2829	6.18E-05	1.67E-01	1.24E-03	5.06E-07
	S342	2	1	1	1	237088316	237669270	580.955	1465	6.18E-05	1.67E-01	1.24E-03	5.06E-07
	S344	2	1	1	1	244196043	244910463	714.421	1634	6.18E-05	1.67E-01	1.24E-03	5.06E-07
	S359	2	1	1	2	190557547	190695863	138.317	247	6.18E-05	1.67E-01	1.24E-03	5.06E-07
	S366	2	1	1	3	43153774	44854369	1700.596	2783	6.18E-05	1.67E-01	1.24E-03	5.06E-07
	S367	2	1	1	3	45134667	45475606	340.94	901	6.18E-05	1.67E-01	1.24E-03	5.06E-07
	S369	2	1	1	3	131751329	132925541	1174.213	2814	6.18E-05	1.67E-01	1.24E-03	5.06E-07
	S373	2	1	1	4	47409708	49019082	1609.375	2903	6.18E-05	1.67E-01	1.24E-03	5.06E-07
	S378	2	1	1	4	86568541	88302712	1734.172	3821	6.18E-05	1.67E-01	1.24E-03	5.06E-07
	S379	2	1	1	4	93835243	94715990	880.748	1974	6.18E-05	1.67E-01	1.24E-03	5.06E-07

	S391	2	1	1	5	158105137	159510174	1405.038	2629	6.18E-05	1.67E-01	1.24E-03	5.06E-07
	S393	2	1	1	6	32836793	35038899	2202.107	4929	6.18E-05	1.67E-01	1.24E-03	5.06E-07
	S394	2	1	1	6	37203403	37594772	391.37	839	6.18E-05	1.67E-01	1.24E-03	5.06E-07
	S395	2	1	1	6	38628081	38730063	101.983	196	6.18E-05	1.67E-01	1.24E-03	5.06E-07
	S396	2	2	0	6	39932281	40995345	1063.065	2334	1.44E-34	3.33E-01	0.00E+00	1.18E-36
	S397	2	2	0	6	42101237	42301027	199.791	468	1.44E-34	3.33E-01	0.00E+00	1.18E-36
	S404	2	1	1	7	22118464	24040285	1921.822	4492	6.18E-05	1.67E-01	1.24E-03	5.06E-07
	S408	2	1	1	7	134332988	134763420	430.433	1166	6.18E-05	1.67E-01	1.24E-03	5.06E-07
	S409	2	2	0	7	136440705	137832195	1391.491	2691	1.44E-34	3.33E-01	0.00E+00	1.18E-36
	S410	2	1	1	7	138410394	141012028	2601.635	5349	6.18E-05	1.67E-01	1.24E-03	5.06E-07
	S411	2	1	1	7	141059113	146879533	5820.421	11327	6.18E-05	1.67E-01	1.24E-03	5.06E-07
	S413	2	1	1	7	147802662	149669205	1866.544	4506	6.18E-05	1.67E-01	1.24E-03	5.06E-07
	S414	2	1	1	7	156160369	157966851	1806.483	4507	6.18E-05	1.67E-01	1.24E-03	5.06E-07
	S420	2	1	1	8	107635268	109528850	1893.583	4533	6.18E-05	1.67E-01	1.24E-03	5.06E-07

	S445	2	1	1	12	11899749	12307869	408.121	898	6.18E-05	1.67E-01	1.24E-03	5.06E-07
	S447	2	1	1	12	24786817	25522117	735.301	1988	6.18E-05	1.67E-01	1.24E-03	5.06E-07
	S457	2	1	1	12	131246290	133220283	1973.994	4851	6.18E-05	1.67E-01	1.24E-03	5.06E-07
	S465	2	1	1	14	53431888	56631839	3199.952	7120	6.18E-05	1.67E-01	1.24E-03	5.06E-07
	S466	2	1	1	14	56967328	58646712	1679.385	3254	6.18E-05	1.67E-01	1.24E-03	5.06E-07
	S469	2	1	1	15	73638536	75161712	1523.177	2644	6.18E-05	1.67E-01	1.24E-03	5.06E-07
	S470	2	1	1	16	55154208	57060732	1906.525	4194	6.18E-05	1.67E-01	1.24E-03	5.06E-07
	S475	2	1	1	17	56980770	58066902	1086.133	2315	6.18E-05	1.67E-01	1.24E-03	5.06E-07
	S481	2	1	1	18	54886830	57310636	2423.807	4444	6.18E-05	1.67E-01	1.24E-03	5.06E-07
	S488	2	1	1	21	18694867	19035647	340.781	1056	6.18E-05	1.67E-01	1.24E-03	5.06E-07
**AFR**	S10	70	4	66	3	48728819	48913826	185.008	105	4.83E-05	3.08E-01	4.05E-02	9.02E-08
	S41	50	4	46	6	28180332	28285755	105.424	192	4.80E-07	3.08E-01	2.82E-02	8.95E-10
	S44	49	5	44	6	61660867	61817292	156.426	394	1.65E-11	3.85E-01	2.70E-02	3.08E-14
	S64	36	4	32	6	29403395	29516457	113.063	270	9.50E-10	3.08E-01	1.96E-02	1.77E-12

	S67	35	5	30	6	29636570	29764813	128.244	510	3.74E-16	3.85E-01	1.84E-02	6.97E-19
	S70	34	3	31	3	84664823	84780882	116.06	286	1.27E-05	2.31E-01	1.90E-02	2.37E-08
	S109	25	3	22	3	83530934	83675403	144.47	330	1.62E-07	2.31E-01	1.35E-02	3.02E-10
	S139	22	4	18	6	31415915	31521867	105.953	129	7.62E-16	3.08E-01	1.10E-02	1.42E-18
	S150	21	3	18	6	63185096	63285572	100.477	224	7.15E-09	2.31E-01	1.10E-02	1.33E-11
	S179	19	3	16	6	68913394	69240716	327.323	639	9.26E-10	2.31E-01	9.81E-03	1.73E-12
	S192	18	3	15	6	35588032	35711345	123.314	177	2.81E-10	2.31E-01	9.20E-03	5.25E-13
	S212	17	3	14	3	43856298	44304154	447.857	697	7.45E-11	2.31E-01	8.58E-03	1.39E-13
	S213	17	3	14	3	52502292	52986528	484.237	999	7.45E-11	2.31E-01	8.58E-03	1.39E-13
	S266	15	4	11	6	48827282	49150144	322.863	802	4.06E-23	3.08E-01	6.74E-03	7.58E-26
	S268	15	2	13	7	85243049	85497596	254.548	609	5.22E-05	1.54E-01	7.97E-03	9.74E-08
	S280	14	2	12	2	57240622	57473421	232.8	688	2.55E-05	1.54E-01	7.36E-03	4.76E-08
	S281	14	2	12	2	60731152	61071377	340.226	551	2.55E-05	1.54E-01	7.36E-03	4.76E-08
	S296	14	2	12	6	74853271	75035474	182.204	280	2.55E-05	1.54E-01	7.36E-03	4.76E-08

	S314	13	2	11	2	57715106	58316108	601.003	1307	1.12E-05	1.54E-01	6.74E-03	2.09E-08
	S323	13	2	11	4	33645388	33917601	272.214	563	1.12E-05	1.54E-01	6.74E-03	2.09E-08
	S324	13	2	11	4	34022455	34483644	461.19	794	1.12E-05	1.54E-01	6.74E-03	2.09E-08
	S336	13	2	11	6	77675019	78007178	332.16	846	1.12E-05	1.54E-01	6.74E-03	2.09E-08
	S337	13	2	11	7	86310360	86552968	242.609	570	1.12E-05	1.54E-01	6.74E-03	2.09E-08
	S347	13	2	11	8	103876393	104330371	453.979	942	1.12E-05	1.54E-01	6.74E-03	2.09E-08
	S349	13	2	11	11	30494135	30609819	115.685	221	1.12E-05	1.54E-01	6.74E-03	2.09E-08
	S379	12	2	10	6	70033950	70185820	151.871	349	4.30E-06	1.54E-01	6.13E-03	8.02E-09
	S381	12	2	10	6	73782476	73933339	150.864	347	4.30E-06	1.54E-01	6.13E-03	8.02E-09
	S390	12	2	10	11	37510967	37618092	107.126	296	4.30E-06	1.54E-01	6.13E-03	8.02E-09
	S433	11	2	9	6	33310149	33514091	203.943	296	1.39E-06	1.54E-01	5.52E-03	2.60E-09
	S434	11	3	8	6	50663077	50992603	329.527	545	1.70E-16	2.31E-01	4.90E-03	3.16E-19
	S435	11	3	8	6	51272039	51682129	410.091	822	1.70E-16	2.31E-01	4.90E-03	3.16E-19
	S437	11	2	9	6	64268326	64533352	265.027	796	1.39E-06	1.54E-01	5.52E-03	2.60E-09

	S439	11	3	8	6	67018676	67198121	179.446	629	1.70E-16	2.31E-01	4.90E-03	3.16E-19
	S441	11	2	9	6	70720341	70955770	235.43	328	1.39E-06	1.54E-01	5.52E-03	2.60E-09
	S442	11	2	9	6	71029738	71360658	330.921	824	1.39E-06	1.54E-01	5.52E-03	2.60E-09
	S443	11	2	9	6	71437857	71584215	146.359	333	1.39E-06	1.54E-01	5.52E-03	2.60E-09
	S444	11	2	9	6	71739873	72255955	516.083	927	1.39E-06	1.54E-01	5.52E-03	2.60E-09
	S445	11	2	9	6	82145707	82257458	111.752	172	1.39E-06	1.54E-01	5.52E-03	2.60E-09
	S448	11	2	9	6	145205964	146492402	1286.439	2362	1.39E-06	1.54E-01	5.52E-03	2.60E-09
	S457	11	2	9	8	104471752	104867718	395.967	887	1.39E-06	1.54E-01	5.52E-03	2.60E-09
	S458	11	2	9	8	108860914	109306803	445.89	1263	1.39E-06	1.54E-01	5.52E-03	2.60E-09
	S459	11	2	9	8	118422426	118958393	535.968	1528	1.39E-06	1.54E-01	5.52E-03	2.60E-09
	S460	11	2	9	8	119391387	119569200	177.814	453	1.39E-06	1.54E-01	5.52E-03	2.60E-09
	S461	11	2	9	9	34421281	34540917	119.637	152	1.39E-06	1.54E-01	5.52E-03	2.60E-09
	S475	11	2	9	15	50284963	50761937	476.975	1168	1.39E-06	1.54E-01	5.52E-03	2.60E-09
	S476	11	2	9	15	50763691	52095388	1331.698	2725	1.39E-06	1.54E-01	5.52E-03	2.60E-09

	S492	10	2	8	2	50664513	51076430	411.918	678	3.61E-07	1.54E-01	4.90E-03	6.73E-10
	S493	10	2	8	2	51258955	51806510	547.556	1650	3.61E-07	1.54E-01	4.90E-03	6.73E-10
	S500	10	3	7	3	34205539	35096242	890.704	1793	4.33E-18	2.31E-01	4.29E-03	8.08E-21
	S502	10	3	7	3	35245121	35519479	274.359	554	4.33E-18	2.31E-01	4.29E-03	8.08E-21
	S504	10	2	8	3	39501530	40162687	661.158	1720	3.61E-07	1.54E-01	4.90E-03	6.73E-10
	S505	10	2	8	3	40476399	40644230	167.832	308	3.61E-07	1.54E-01	4.90E-03	6.73E-10
	S506	10	3	7	3	45397767	45525262	127.496	246	4.33E-18	2.31E-01	4.29E-03	8.08E-21
	S524	10	3	7	6	49560470	49682868	122.399	278	4.33E-18	2.31E-01	4.29E-03	8.08E-21
	S525	10	3	7	6	51822479	51946991	124.513	253	4.33E-18	2.31E-01	4.29E-03	8.08E-21
	S526	10	3	7	6	66378735	66621187	242.453	711	4.33E-18	2.31E-01	4.29E-03	8.08E-21
	S527	10	3	7	6	66692713	66847728	155.016	413	4.33E-18	2.31E-01	4.29E-03	8.08E-21
	S530	10	2	8	6	79811557	79982290	170.734	460	3.61E-07	1.54E-01	4.90E-03	6.73E-10
	S531	10	2	8	6	80223177	80479678	256.502	728	3.61E-07	1.54E-01	4.90E-03	6.73E-10
	S532	10	2	8	6	81168417	81390898	222.482	527	3.61E-07	1.54E-01	4.90E-03	6.73E-10

	S543	10	2	8	8	117783209	118268462	485.254	1110	3.61E-07	1.54E-01	4.90E-03	6.73E-10
	S573	9	2	7	1	176442466	176780654	338.189	464	6.97E-08	1.54E-01	4.29E-03	1.30E-10
	S574	9	2	7	1	178525293	178959438	434.146	958	6.97E-08	1.54E-01	4.29E-03	1.30E-10
	S586	9	3	6	3	57018060	58070339	1052.28	2016	4.92E-20	2.31E-01	3.68E-03	9.17E-23
	S610	9	2	7	6	76973954	77110554	136.601	330	6.97E-08	1.54E-01	4.29E-03	1.30E-10
	S613	9	2	7	6	82537954	82724948	186.995	411	6.97E-08	1.54E-01	4.29E-03	1.30E-10
	S616	9	2	7	6	98490958	98745037	254.08	288	6.97E-08	1.54E-01	4.29E-03	1.30E-10
	S632	9	2	7	8	105052808	105493352	440.545	791	6.97E-08	1.54E-01	4.29E-03	1.30E-10
	S634	9	2	7	9	34768208	34889377	121.17	275	6.97E-08	1.54E-01	4.29E-03	1.30E-10
	S646	9	2	7	11	105048665	105927769	879.105	1773	6.97E-08	1.54E-01	4.29E-03	1.30E-10
	S647	9	2	7	11	106059457	106368624	309.168	580	6.97E-08	1.54E-01	4.29E-03	1.30E-10
	S669	8	2	6	1	177287748	177464141	176.394	323	8.98E-09	1.54E-01	3.68E-03	1.67E-11
	S683	8	3	5	3	22888766	24045642	1156.877	2574	1.84E-22	2.31E-01	3.07E-03	3.42E-25
	S685	8	3	5	3	56812041	56955359	143.319	296	1.84E-22	2.31E-01	3.07E-03	3.42E-25

	S740	8	2	6	8	115750076	115939770	189.695	319	8.98E-09	1.54E-01	3.68E-03	1.67E-11
	S741	8	2	6	8	120534340	120829101	294.762	692	8.98E-09	1.54E-01	3.68E-03	1.67E-11
	S746	8	2	6	10	55234687	55377027	142.341	289	8.98E-09	1.54E-01	3.68E-03	1.67E-11
	S765	8	2	6	13	64055722	64494100	438.379	1104	8.98E-09	1.54E-01	3.68E-03	1.67E-11
	S817	7	2	5	3	35998558	36224210	225.653	637	6.50E-10	1.54E-01	3.07E-03	1.21E-12
	S818	7	3	4	3	56397760	56731411	333.652	737	1.40E-25	2.31E-01	2.45E-03	2.61E-28
	S827	7	2	5	4	59934135	60574908	640.774	1766	6.50E-10	1.54E-01	3.07E-03	1.21E-12
	S828	7	2	5	4	60784754	61001838	217.085	519	6.50E-10	1.54E-01	3.07E-03	1.21E-12
	S857	7	2	5	6	38809086	39102251	293.166	685	6.50E-10	1.54E-01	3.07E-03	1.21E-12
	S858	7	2	5	6	39883665	40116932	233.268	571	6.50E-10	1.54E-01	3.07E-03	1.21E-12
	S859	7	2	5	6	40157348	41326358	1169.011	2675	6.50E-10	1.54E-01	3.07E-03	1.21E-12
	S874	7	2	5	7	82744461	83107490	363.03	865	6.50E-10	1.54E-01	3.07E-03	1.21E-12
	S875	7	2	5	7	83393186	83632522	239.337	716	6.50E-10	1.54E-01	3.07E-03	1.21E-12
	S883	7	2	5	8	116588023	117399354	811.332	1861	6.50E-10	1.54E-01	3.07E-03	1.21E-12

	S904	7	2	5	11	108629089	109973023	1343.935	2537	6.50E-10	1.54E-01	3.07E-03	1.21E-12
	S905	7	2	5	11	110515513	110640876	125.364	301	6.50E-10	1.54E-01	3.07E-03	1.21E-12
	S906	7	2	5	11	111296046	112587400	1291.355	2222	6.50E-10	1.54E-01	3.07E-03	1.21E-12
	S935	6	2	4	1	168418335	168719795	301.461	852	1.98E-11	1.54E-01	2.45E-03	3.70E-14
	S936	6	2	4	1	168930219	169202299	272.081	705	1.98E-11	1.54E-01	2.45E-03	3.70E-14
	S937	6	2	4	1	169225917	169329424	103.508	244	1.98E-11	1.54E-01	2.45E-03	3.70E-14
	S968	6	2	4	3	37228187	37466256	238.07	462	1.98E-11	1.54E-01	2.45E-03	3.70E-14
	S969	6	2	4	3	38012187	38353299	341.113	537	1.98E-11	1.54E-01	2.45E-03	3.70E-14
	S1011	6	2	4	6	36751302	36964044	212.743	534	1.98E-11	1.54E-01	2.45E-03	3.70E-14
	S1040	6	2	4	8	105780711	106128237	347.527	850	1.98E-11	1.54E-01	2.45E-03	3.70E-14
	S1041	6	2	4	8	107345781	107747646	401.866	1020	1.98E-11	1.54E-01	2.45E-03	3.70E-14
	S1045	6	2	4	9	36138335	36282451	144.117	322	1.98E-11	1.54E-01	2.45E-03	3.70E-14
	S1073	6	2	4	11	107126130	107965328	839.199	2034	1.98E-11	1.54E-01	2.45E-03	3.70E-14
	S1128	5	2	3	1	58060710	58589588	528.879	1285	1.52E-13	1.54E-01	1.84E-03	2.84E-16

	S1143	5	2	3	2	47646473	48759335	1112.863	2080	1.52E-13	1.54E-01	1.84E-03	2.84E-16
	S1144	5	2	3	2	49056215	49415607	359.393	946	1.52E-13	1.54E-01	1.84E-03	2.84E-16
	S1147	5	2	3	2	55118173	55435262	317.09	760	1.52E-13	1.54E-01	1.84E-03	2.84E-16
	S1159	5	2	3	3	54998321	55586021	587.701	1521	1.52E-13	1.54E-01	1.84E-03	2.84E-16
	S1199	5	2	3	6	37144230	37261415	117.186	226	1.52E-13	1.54E-01	1.84E-03	2.84E-16
	S1200	5	2	3	6	37356336	38349910	993.575	2186	1.52E-13	1.54E-01	1.84E-03	2.84E-16
	S1202	5	2	3	6	86802921	87341282	538.362	1289	1.52E-13	1.54E-01	1.84E-03	2.84E-16
	S1236	5	2	3	8	96157210	97227476	1070.267	2080	1.52E-13	1.54E-01	1.84E-03	2.84E-16
	S1257	5	2	3	11	40341946	40743942	401.997	1040	1.52E-13	1.54E-01	1.84E-03	2.84E-16
	S1297	5	2	3	17	63596386	64036726	440.341	978	1.52E-13	1.54E-01	1.84E-03	2.84E-16
	S1322	4	2	2	1	112485167	112789878	304.712	604	1.05E-16	1.54E-01	1.23E-03	1.96E-19
	S1393	4	2	2	6	22038571	22214312	175.742	399	1.05E-16	1.54E-01	1.23E-03	1.96E-19
	S1395	4	2	2	6	87947837	88082697	134.861	262	1.05E-16	1.54E-01	1.23E-03	1.96E-19
	S1404	4	2	2	6	147236947	147857732	620.786	1324	1.05E-16	1.54E-01	1.23E-03	1.96E-19

	S1434	4	2	2	8	101488186	101689246	201.061	412	1.05E-16	1.54E-01	1.23E-03	1.96E-19
	S1446	4	2	2	9	79979163	80353847	374.685	846	1.05E-16	1.54E-01	1.23E-03	1.96E-19
	S1447	4	2	2	9	84384006	84513082	129.077	340	1.05E-16	1.54E-01	1.23E-03	1.96E-19
	S1466	4	2	2	11	25505018	26000335	495.318	1393	1.05E-16	1.54E-01	1.23E-03	1.96E-19
	S1467	4	2	2	11	26543019	26749689	206.671	609	1.05E-16	1.54E-01	1.23E-03	1.96E-19
	S1606	3	2	1	6	44569600	46097197	1527.598	3136	5.87E-22	1.54E-01	6.13E-04	1.10E-24
	S1634	3	2	1	9	81631892	82533369	901.478	1237	5.87E-22	1.54E-01	6.13E-04	1.10E-24
	S1635	3	2	1	9	82808145	83095464	287.32	554	5.87E-22	1.54E-01	6.13E-04	1.10E-24
	S1657	3	2	1	11	24352804	24732818	380.015	1110	5.87E-22	1.54E-01	6.13E-04	1.10E-24
	S1658	3	2	1	11	24749917	24895125	145.209	484	5.87E-22	1.54E-01	6.13E-04	1.10E-24
	S1693	3	2	1	15	53478028	54002900	524.873	1648	5.87E-22	1.54E-01	6.13E-04	1.10E-24
	S1756	2	2	0	4	37930085	39539792	1609.708	3733	2.00E-32	1.54E-01	0.00E+00	3.73E-35
**AJ**	S2	51	15	36	6	61517308	61819485	302.178	809	1.73E-06	3.06E-01	7.86E-02	1.63E-09
	S34	22	9	13	12	38519595	38765639	246.045	329	2.57E-06	1.84E-01	2.84E-02	2.43E-09

	S62	17	8	9	13	88118485	88486560	368.076	639	1.01E-06	1.63E-01	1.97E-02	9.51E-10
	S74	16	8	8	13	87912381	88019280	106.9	136	3.08E-07	1.63E-01	1.75E-02	2.90E-10
	S88	15	8	7	13	89818363	90125024	306.662	848	8.01E-08	1.63E-01	1.53E-02	7.56E-11
	S102	14	8	6	13	87051055	87541111	490.057	808	1.72E-08	1.63E-01	1.31E-02	1.62E-11
	S124	13	7	6	13	86174511	86441080	266.57	416	6.16E-07	1.43E-01	1.31E-02	5.81E-10
	S131	12	6	6	2	62598168	64070030	1471.863	1777	1.78E-05	1.22E-01	1.31E-02	1.68E-08
	S151	12	7	5	12	40624560	40770009	145.45	277	1.29E-07	1.43E-01	1.09E-02	1.22E-10
	S152	12	8	4	12	40788769	41954393	1165.625	2219	3.64E-10	1.63E-01	8.73E-03	3.43E-13
	S153	12	8	4	12	42529792	42653120	123.329	241	3.64E-10	1.63E-01	8.73E-03	3.43E-13
	S170	11	6	5	3	57039765	57148468	108.704	184	4.71E-06	1.22E-01	1.09E-02	4.44E-09
	S210	11	7	4	20	24683099	25045238	362.14	652	2.03E-08	1.43E-01	8.73E-03	1.92E-11
	S268	10	6	4	12	30971682	31113379	141.698	292	9.56E-07	1.22E-01	8.73E-03	9.03E-10
	S269	10	7	3	12	43524729	43854597	329.869	511	2.21E-09	1.43E-01	6.55E-03	2.09E-12
	S270	10	7	3	12	43922682	44327091	404.41	389	2.21E-09	1.43E-01	6.55E-03	2.09E-12

	S271	10	7	3	12	44649310	45574415	925.106	963	2.21E-09	1.43E-01	6.55E-03	2.09E-12
	S272	10	6	4	12	45610378	45913265	302.888	231	9.56E-07	1.22E-01	8.73E-03	9.03E-10
	S297	10	7	3	20	23291185	23613694	322.51	537	2.21E-09	1.43E-01	6.55E-03	2.09E-12
	S340	9	5	4	5	60276522	61263213	986.692	1263	3.59E-05	1.02E-01	8.73E-03	3.39E-08
	S372	9	5	4	8	77682014	78472684	790.671	1224	3.59E-05	1.02E-01	8.73E-03	3.39E-08
	S404	9	5	4	13	38618291	38770772	152.482	190	3.59E-05	1.02E-01	8.73E-03	3.39E-08
	S410	9	5	4	16	58499171	58934725	435.555	942	3.59E-05	1.02E-01	8.73E-03	3.39E-08
	S411	9	5	4	16	59418341	59854704	436.364	748	3.59E-05	1.02E-01	8.73E-03	3.39E-08
	S418	9	7	2	20	20915880	22336148	1420.269	1464	1.47E-10	1.43E-01	4.37E-03	1.39E-13
	S434	8	6	2	2	20934251	22009115	1074.865	1125	1.19E-08	1.22E-01	4.37E-03	1.12E-11
	S435	8	6	2	2	26666708	28199122	1532.415	1444	1.19E-08	1.22E-01	4.37E-03	1.12E-11
	S436	8	5	3	2	28465602	28567825	102.224	210	6.96E-06	1.02E-01	6.55E-03	6.57E-09
	S502	8	5	3	7	85938095	87231016	1292.922	1935	6.96E-06	1.02E-01	6.55E-03	6.57E-09
	S523	8	5	3	8	73072293	73226862	154.57	281	6.96E-06	1.02E-01	6.55E-03	6.57E-09

	S555	8	5	3	12	30221394	30789240	567.847	1299	6.96E-06	1.02E-01	6.55E-03	6.57E-09
	S570	8	5	3	13	77460429	78399696	939.268	1580	6.96E-06	1.02E-01	6.55E-03	6.57E-09
	S589	8	5	3	16	55710216	57282328	1572.113	2370	6.96E-06	1.02E-01	6.55E-03	6.57E-09
	S597	8	5	3	20	17272348	17556261	283.914	627	6.96E-06	1.02E-01	6.55E-03	6.57E-09
	S598	8	6	2	20	17908406	18918138	1009.733	2049	1.19E-08	1.22E-01	4.37E-03	1.12E-11
	S601	8	5	3	21	44532043	46078324	1546.282	3176	6.96E-06	1.02E-01	6.55E-03	6.57E-09
	S627	7	5	2	2	22461063	23007989	546.927	668	8.44E-07	1.02E-01	4.37E-03	7.97E-10
	S784	7	5	2	11	107182466	107691854	509.389	896	8.44E-07	1.02E-01	4.37E-03	7.97E-10
	S785	7	5	2	11	107994281	108606766	612.486	691	8.44E-07	1.02E-01	4.37E-03	7.97E-10
	S823	7	5	2	20	19128902	19634799	505.898	713	8.44E-07	1.02E-01	4.37E-03	7.97E-10
	S825	7	5	2	20	38320055	39059954	739.9	953	8.44E-07	1.02E-01	4.37E-03	7.97E-10
	S832	7	5	2	21	43194143	44092986	898.844	1041	8.44E-07	1.02E-01	4.37E-03	7.97E-10
	S1080	6	5	1	20	12312034	12754957	442.924	812	5.08E-08	1.02E-01	2.18E-03	4.79E-11
	S1081	6	5	1	20	14529989	15582464	1052.476	1783	5.08E-08	1.02E-01	2.18E-03	4.79E-11

	S1106	5	4	1	1	204388918	205845567	1456.65	2230	4.46E-06	8.16E-02	2.18E-03	4.21E-09
	S1107	5	4	1	1	212431624	212590188	158.565	340	4.46E-06	8.16E-02	2.18E-03	4.21E-09
	S1116	5	4	1	2	64961311	65397338	436.028	785	4.46E-06	8.16E-02	2.18E-03	4.21E-09
	S1296	5	4	1	16	73656643	74025872	369.23	432	4.46E-06	8.16E-02	2.18E-03	4.21E-09
	S1325	5	4	1	21	39889466	39994119	104.654	264	4.46E-06	8.16E-02	2.18E-03	4.21E-09
	S1341	4	4	0	1	159797454	159943263	145.81	263	1.23E-07	8.16E-02	0.00E+00	1.16E-10
	S1443	4	4	0	8	125749215	127191815	1442.601	2246	1.23E-07	8.16E-02	0.00E+00	1.16E-10
	S1623	3	3	0	9	1940954	3629808	1688.855	3403	1.48E-05	6.12E-02	0.00E+00	1.40E-08
	S1650	3	3	0	11	2896280	3419246	522.967	955	1.48E-05	6.12E-02	0.00E+00	1.40E-08
	S1651	3	3	0	11	4879183	5223561	344.379	857	1.48E-05	6.12E-02	0.00E+00	1.40E-08
	S1665	3	3	0	13	22346187	23414536	1068.35	2389	1.48E-05	6.12E-02	0.00E+00	1.40E-08
	S1666	3	3	0	13	73852645	74103309	250.665	391	1.48E-05	6.12E-02	0.00E+00	1.40E-08
**AMR**	S77	5	4	1	8	42230547	43554958	1324.412	1012	8.34E-05	2.00E-01	7.19E-03	2.38E-07
	S81	5	4	1	9	79266337	80842614	1576.278	2893	8.34E-05	2.00E-01	7.19E-03	2.38E-07

	S82	5	5	0	9	82135547	84169832	2034.286	2754	1.13E-07	2.50E-01	0.00E+00	3.22E-10
	S88	5	4	1	17	20558851	20936047	377.197	353	8.34E-05	2.00E-01	7.19E-03	2.38E-07
	S121	4	4	0	10	24128513	25123571	995.059	1380	4.73E-06	2.00E-01	0.00E+00	1.35E-08
	S122	4	4	0	10	25129805	25475212	345.408	485	4.73E-06	2.00E-01	0.00E+00	1.35E-08
	S136	4	4	0	17	9973671	10259230	285.56	658	4.73E-06	2.00E-01	0.00E+00	1.35E-08
**CAS**	S42	11	4	7	2	97020004	97717987	697.984	133	6.47E-05	2.67E-01	2.62E-02	1.13E-07
	S48	11	4	7	8	98529140	99858154	1329.015	696	6.47E-05	2.67E-01	2.62E-02	1.13E-07
	S68	9	4	5	7	118714624	118870761	156.138	156	5.09E-06	2.67E-01	1.87E-02	8.85E-09
	S138	6	3	3	2	140629282	141846442	1217.161	2202	6.07E-05	2.00E-01	1.12E-02	1.05E-07
	S139	6	3	3	2	141862174	141991767	129.594	186	6.07E-05	2.00E-01	1.12E-02	1.05E-07
	S141	6	3	3	2	147415548	148475487	1059.94	1237	6.07E-05	2.00E-01	1.12E-02	1.05E-07
	S176	6	3	3	7	43944655	44218344	273.69	375	6.07E-05	2.00E-01	1.12E-02	1.05E-07
	S187	6	3	3	8	113104457	113490131	385.675	488	6.07E-05	2.00E-01	1.12E-02	1.05E-07
	S199	6	3	3	11	97257693	97804305	546.613	649	6.07E-05	2.00E-01	1.12E-02	1.05E-07

	S201	6	4	2	12	40662517	42677591	2015.075	3661	4.94E-09	2.67E-01	7.49E-03	8.60E-12
	S207	6	3	3	17	18258430	18872587	614.158	523	6.07E-05	2.00E-01	1.12E-02	1.05E-07
	S232	5	3	2	2	103942665	104621405	678.741	837	7.06E-06	2.00E-01	7.49E-03	1.23E-08
	S234	5	3	2	2	112340863	113461230	1120.368	1486	7.06E-06	2.00E-01	7.49E-03	1.23E-08
	S235	5	3	2	2	143505975	143954195	448.221	606	7.06E-06	2.00E-01	7.49E-03	1.23E-08
	S236	5	3	2	2	143969076	144543747	574.672	387	7.06E-06	2.00E-01	7.49E-03	1.23E-08
	S237	5	3	2	2	148806575	149304921	498.347	580	7.06E-06	2.00E-01	7.49E-03	1.23E-08
	S239	5	3	2	2	151144438	152252685	1108.248	1397	7.06E-06	2.00E-01	7.49E-03	1.23E-08
	S240	5	3	2	2	152328192	153142511	814.32	1480	7.06E-06	2.00E-01	7.49E-03	1.23E-08
	S241	5	4	1	2	158883384	160573098	1689.715	2874	7.89E-11	2.67E-01	3.75E-03	1.37E-13
	S276	5	3	2	4	132016378	132804596	788.219	1458	7.06E-06	2.00E-01	7.49E-03	1.23E-08
	S277	5	3	2	4	141052916	141188498	135.583	165	7.06E-06	2.00E-01	7.49E-03	1.23E-08
	S278	5	3	2	4	141239910	141557767	317.858	423	7.06E-06	2.00E-01	7.49E-03	1.23E-08
	S308	5	3	2	7	47666099	48697848	1031.75	1762	7.06E-06	2.00E-01	7.49E-03	1.23E-08

	S311	5	3	2	7	132498641	134063722	1565.082	1708	7.06E-06	2.00E-01	7.49E-03	1.23E-08
	S341	5	3	2	12	44863086	47069014	2205.929	2919	7.06E-06	2.00E-01	7.49E-03	1.23E-08
	S353	5	3	2	17	14867684	17192330	2324.647	2715	7.06E-06	2.00E-01	7.49E-03	1.23E-08
	S354	5	3	2	17	18960666	19329720	369.055	178	7.06E-06	2.00E-01	7.49E-03	1.23E-08
	S382	4	3	1	2	23540706	24466643	925.938	1132	2.86E-07	2.00E-01	3.75E-03	4.97E-10
	S384	4	3	1	2	29386339	30267999	881.661	1290	2.86E-07	2.00E-01	3.75E-03	4.97E-10
	S385	4	3	1	2	49772441	49972265	199.825	297	2.86E-07	2.00E-01	3.75E-03	4.97E-10
	S386	4	3	1	2	51866901	52025961	159.061	234	2.86E-07	2.00E-01	3.75E-03	4.97E-10
	S390	4	3	1	2	99497959	100604995	1107.037	1328	2.86E-07	2.00E-01	3.75E-03	4.97E-10
	S391	4	3	1	2	100722823	101159757	436.935	722	2.86E-07	2.00E-01	3.75E-03	4.97E-10
	S394	4	3	1	2	154281988	154575357	293.37	477	2.86E-07	2.00E-01	3.75E-03	4.97E-10
	S395	4	3	1	2	154595043	155643731	1048.689	1387	2.86E-07	2.00E-01	3.75E-03	4.97E-10
	S396	4	3	1	2	155825013	156877938	1052.926	1015	2.86E-07	2.00E-01	3.75E-03	4.97E-10
	S397	4	3	1	2	162599870	162754693	154.824	202	2.86E-07	2.00E-01	3.75E-03	4.97E-10

	S398	4	3	1	2	165453151	167634399	2181.249	3154	2.86E-07	2.00E-01	3.75E-03	4.97E-10
	S399	4	3	1	2	167644704	167844661	199.958	360	2.86E-07	2.00E-01	3.75E-03	4.97E-10
	S452	4	3	1	6	33994939	35614884	1619.946	1978	2.86E-07	2.00E-01	3.75E-03	4.97E-10
	S457	4	4	0	6	110411111	111140162	729.052	1070	1.62E-13	2.67E-01	0.00E+00	2.83E-16
	S458	4	3	1	6	114136451	114259142	122.692	110	2.86E-07	2.00E-01	3.75E-03	4.97E-10
	S459	4	3	1	6	114278343	116101118	1822.776	2629	2.86E-07	2.00E-01	3.75E-03	4.97E-10
	S460	4	3	1	6	116501286	117285199	783.914	1107	2.86E-07	2.00E-01	3.75E-03	4.97E-10
	S484	4	4	0	8	96251190	97440997	1189.808	1302	1.62E-13	2.67E-01	0.00E+00	2.83E-16
	S486	4	3	1	8	98086716	98247413	160.698	268	2.86E-07	2.00E-01	3.75E-03	4.97E-10
	S511	4	3	1	11	75194690	76847851	1653.162	2032	2.86E-07	2.00E-01	3.75E-03	4.97E-10
	S512	4	3	1	11	84667999	86999400	2331.402	3319	2.86E-07	2.00E-01	3.75E-03	4.97E-10
	S513	4	3	1	11	87008557	87247736	239.18	542	2.86E-07	2.00E-01	3.75E-03	4.97E-10
	S514	4	3	1	11	87248673	87691952	443.28	779	2.86E-07	2.00E-01	3.75E-03	4.97E-10
	S518	4	3	1	11	102339006	104987712	2648.707	4346	2.86E-07	2.00E-01	3.75E-03	4.97E-10

	S651	3	3	0	6	36648823	39157799	2508.977	4342	1.42E-09	2.00E-01	0.00E+00	2.47E-12
	S652	3	3	0	6	39766113	40372145	606.033	888	1.42E-09	2.00E-01	0.00E+00	2.47E-12
	S822	2	2	0	1	210614853	210878283	263.431	521	1.05E-05	1.33E-01	0.00E+00	1.82E-08
	S825	2	2	0	2	10879840	12560515	1680.676	2478	1.05E-05	1.33E-01	0.00E+00	1.82E-08
	S826	2	2	0	2	12951890	15863284	2911.395	4475	1.05E-05	1.33E-01	0.00E+00	1.82E-08
	S827	2	2	0	2	16368968	17996554	1627.587	2063	1.05E-05	1.33E-01	0.00E+00	1.82E-08
	S828	2	2	0	2	18171735	20375251	2203.517	3441	1.05E-05	1.33E-01	0.00E+00	1.82E-08
	S840	2	2	0	2	223312391	225222611	1910.221	2874	1.05E-05	1.33E-01	0.00E+00	1.82E-08
	S841	2	2	0	2	225434130	227933798	2499.669	3670	1.05E-05	1.33E-01	0.00E+00	1.82E-08
	S842	2	2	0	2	227938269	229819388	1881.12	2709	1.05E-05	1.33E-01	0.00E+00	1.82E-08
	S901	2	2	0	6	23750907	23913300	162.394	392	1.05E-05	1.33E-01	0.00E+00	1.82E-08
	S903	2	2	0	6	125419743	127356617	1936.875	1330	1.05E-05	1.33E-01	0.00E+00	1.82E-08
	S983	2	2	0	15	55999329	57329488	1330.16	1791	1.05E-05	1.33E-01	0.00E+00	1.82E-08
	S984	2	2	0	15	58538767	60687613	2148.847	2852	1.05E-05	1.33E-01	0.00E+00	1.82E-08

	S1009	2	2	0	19	3561221	3931179	369.959	586	1.05E-05	1.33E-01	0.00E+00	1.82E-08
**EAS**	S140	71	3	68	10	73002790	73383033	380.244	306	5.62E-05	3.00E-01	2.95E-02	1.36E-07
	S427	22	2	20	6	145191755	146344288	1152.534	1464	4.52E-06	2.00E-01	8.69E-03	1.10E-08
	S438	21	2	19	1	102554496	103131712	577.217	564	2.51E-06	2.00E-01	8.25E-03	6.09E-09
	S459	21	2	19	11	85770648	86122418	351.771	384	2.51E-06	2.00E-01	8.25E-03	6.09E-09
	S461	21	2	19	14	40984076	41132848	148.773	364	2.51E-06	2.00E-01	8.25E-03	6.09E-09
	S466	20	2	18	1	103437627	103799249	361.623	109	1.32E-06	2.00E-01	7.82E-03	3.19E-09
	S488	20	2	18	10	74084639	74226087	141.449	149	1.32E-06	2.00E-01	7.82E-03	3.19E-09
	S509	19	2	17	6	50667413	51009564	342.152	363	6.46E-07	2.00E-01	7.38E-03	1.57E-09
	S510	19	2	17	6	55218009	55320140	102.132	146	6.46E-07	2.00E-01	7.38E-03	1.57E-09
	S511	19	2	17	6	55330098	55541872	211.775	365	6.46E-07	2.00E-01	7.38E-03	1.57E-09
	S515	19	2	17	8	77709107	78018344	309.238	479	6.46E-07	2.00E-01	7.38E-03	1.57E-09
	S540	18	2	16	6	86358359	86643756	285.398	492	2.93E-07	2.00E-01	6.95E-03	7.11E-10
	S579	17	3	14	12	85074606	85243787	169.182	150	2.24E-19	3.00E-01	6.08E-03	5.43E-22

	S590	16	2	14	5	88220002	88640561	420.56	369	4.51E-08	2.00E-01	6.08E-03	1.09E-10
	S612	16	2	14	14	41867372	42243063	375.692	533	4.51E-08	2.00E-01	6.08E-03	1.09E-10
	S622	15	2	13	1	92380023	93057526	677.504	462	1.47E-08	2.00E-01	5.65E-03	3.57E-11
	S648	15	3	12	8	82515228	82813602	298.375	451	7.10E-22	3.00E-01	5.21E-03	1.72E-24
	S654	15	2	13	11	82784557	82985843	201.287	221	1.47E-08	2.00E-01	5.65E-03	3.57E-11
	S656	15	2	13	12	83343594	83722633	379.04	687	1.47E-08	2.00E-01	5.65E-03	3.57E-11
	S657	15	2	13	12	83777529	84602145	824.617	1421	1.47E-08	2.00E-01	5.65E-03	3.57E-11
	S670	14	2	12	1	27697089	28078183	381.095	287	4.10E-09	2.00E-01	5.21E-03	9.95E-12
	S672	14	2	12	2	139938532	140069194	130.663	134	4.10E-09	2.00E-01	5.21E-03	9.95E-12
	S689	14	2	12	6	55850178	55985642	135.465	133	4.10E-09	2.00E-01	5.21E-03	9.95E-12
	S695	14	2	12	8	86333471	86477333	143.863	115	4.10E-09	2.00E-01	5.21E-03	9.95E-12
	S707	14	2	12	17	28002165	28888684	886.52	399	4.10E-09	2.00E-01	5.21E-03	9.95E-12
	S715	13	3	10	2	146610620	147097731	487.112	535	3.88E-25	3.00E-01	4.34E-03	9.41E-28
	S742	13	2	11	8	83886231	84059832	173.602	217	9.41E-10	2.00E-01	4.78E-03	2.28E-12

	S744	13	2	11	8	87259956	87449608	189.653	285	9.41E-10	2.00E-01	4.78E-03	2.28E-12
	S753	13	3	10	12	81497836	81620912	123.077	146	3.88E-25	3.00E-01	4.34E-03	9.41E-28
	S758	13	2	11	14	42863867	43859856	995.99	1748	9.41E-10	2.00E-01	4.78E-03	2.28E-12
	S803	12	2	10	11	104564908	104890662	325.755	326	1.70E-10	2.00E-01	4.34E-03	4.12E-13
	S804	12	3	9	12	80907722	81017645	109.924	182	3.56E-27	3.00E-01	3.91E-03	8.63E-30
	S864	11	2	9	8	86633621	86799281	165.661	225	2.25E-11	2.00E-01	3.91E-03	5.45E-14
	S878	11	2	9	11	105086766	105227380	140.615	128	2.25E-11	2.00E-01	3.91E-03	5.45E-14
	S882	11	2	9	13	66334267	66451173	116.907	149	2.25E-11	2.00E-01	3.91E-03	5.45E-14
	S904	10	2	8	2	24027952	24305626	277.675	293	1.99E-12	2.00E-01	3.48E-03	4.84E-15
	S979	10	2	8	13	66950964	67151966	201.003	174	1.99E-12	2.00E-01	3.48E-03	4.84E-15
	S994	9	2	7	1	34934701	36260026	1325.326	1061	1.04E-13	2.00E-01	3.04E-03	2.52E-16
	S996	9	2	7	1	96138507	96403485	264.979	216	1.04E-13	2.00E-01	3.04E-03	2.52E-16
	S1003	9	2	7	2	24428996	25047564	618.569	702	1.04E-13	2.00E-01	3.04E-03	2.52E-16
	S1058	9	2	7	7	110829559	111176421	346.863	288	1.04E-13	2.00E-01	3.04E-03	2.52E-16

	S1059	9	2	7	7	114634276	114902722	268.447	217	1.04E-13	2.00E-01	3.04E-03	2.52E-16
	S1079	9	2	7	11	84504716	84644243	139.528	110	1.04E-13	2.00E-01	3.04E-03	2.52E-16
	S1092	9	2	7	17	30226621	31418963	1192.343	776	1.04E-13	2.00E-01	3.04E-03	2.52E-16
	S1100	8	2	6	1	96703314	96878909	175.596	197	2.60E-15	2.00E-01	2.61E-03	6.32E-18
	S1104	8	2	6	1	246691322	246868937	177.616	273	2.60E-15	2.00E-01	2.61E-03	6.32E-18
	S1105	8	2	6	2	22737657	22861991	124.335	173	2.60E-15	2.00E-01	2.61E-03	6.32E-18
	S1149	8	2	6	6	142585268	142688704	103.437	132	2.60E-15	2.00E-01	2.61E-03	6.32E-18
	S1156	8	2	6	7	145227251	145448233	220.983	222	2.60E-15	2.00E-01	2.61E-03	6.32E-18
	S1157	8	2	6	7	145683698	146174758	491.061	477	2.60E-15	2.00E-01	2.61E-03	6.32E-18
	S1179	8	2	6	11	106622331	107246772	624.442	922	2.60E-15	2.00E-01	2.61E-03	6.32E-18
	S1215	7	2	5	1	247268027	247387029	119.003	106	2.30E-17	2.00E-01	2.17E-03	5.57E-20
	S1216	7	2	5	2	22891705	23008130	116.426	133	2.30E-17	2.00E-01	2.17E-03	5.57E-20
	S1242	7	2	5	4	60634189	60811323	177.135	322	2.30E-17	2.00E-01	2.17E-03	5.57E-20
	S1288	7	2	5	6	143218743	143325646	106.904	172	2.30E-17	2.00E-01	2.17E-03	5.57E-20

	S1291	7	2	5	7	112627005	112886665	259.661	143	2.30E-17	2.00E-01	2.17E-03	5.57E-20
	S1318	7	2	5	12	78309047	78650119	341.073	477	2.30E-17	2.00E-01	2.17E-03	5.57E-20
	S1319	7	2	5	12	78902944	79293191	390.248	366	2.30E-17	2.00E-01	2.17E-03	5.57E-20
	S1351	6	2	4	1	97658971	98253450	594.48	428	4.23E-20	2.00E-01	1.74E-03	1.03E-22
	S1461	6	2	4	21	22990735	23242777	252.043	458	4.23E-20	2.00E-01	1.74E-03	1.03E-22
	S1462	6	2	4	21	23279959	23728393	448.435	645	4.23E-20	2.00E-01	1.74E-03	1.03E-22
	S1463	6	2	4	21	23945679	24103230	157.552	248	4.23E-20	2.00E-01	1.74E-03	1.03E-22
	S1466	5	2	3	1	33459576	33579832	120.257	147	6.39E-24	2.00E-01	1.30E-03	1.55E-26
	S1533	5	2	3	5	14069612	14594268	524.657	447	6.39E-24	2.00E-01	1.30E-03	1.55E-26
	S1552	5	2	3	7	26981837	27382217	400.381	382	6.39E-24	2.00E-01	1.30E-03	1.55E-26
	S1563	5	2	3	7	111793692	112144480	350.789	322	6.39E-24	2.00E-01	1.30E-03	1.55E-26
	S1583	5	2	3	10	82192350	82870159	677.81	760	6.39E-24	2.00E-01	1.30E-03	1.55E-26
	S1708	4	2	2	5	81803826	82590479	786.654	565	1.22E-29	2.00E-01	8.69E-04	2.95E-32
	S1763	4	2	2	11	80489125	80942904	453.78	635	1.22E-29	2.00E-01	8.69E-04	2.95E-32

	S1812	3	1	2	1	99813047	101218347	1405.301	1252	1.81E-05	1.00E-01	8.69E-04	4.39E-08
	S1813	3	1	2	1	106456826	107053821	596.996	603	1.81E-05	1.00E-01	8.69E-04	4.39E-08
	S1814	3	1	2	1	116879836	117138990	259.155	271	1.81E-05	1.00E-01	8.69E-04	4.39E-08
	S1816	3	1	2	1	159972666	160170862	198.197	224	1.81E-05	1.00E-01	8.69E-04	4.39E-08
	S1818	3	1	2	1	171080339	171247758	167.42	242	1.81E-05	1.00E-01	8.69E-04	4.39E-08
	S1823	3	2	1	1	242657535	244144327	1486.793	1452	3.70E-39	2.00E-01	4.34E-04	8.99E-42
	S1824	3	1	2	2	18373851	20413320	2039.47	2497	1.81E-05	1.00E-01	8.69E-04	4.39E-08
	S1825	3	2	1	2	30337825	30769204	431.38	784	3.70E-39	2.00E-01	4.34E-04	8.99E-42
	S1826	3	1	2	2	34634477	35711093	1076.617	1873	1.81E-05	1.00E-01	8.69E-04	4.39E-08
	S1833	3	1	2	2	118270876	118746725	475.85	470	1.81E-05	1.00E-01	8.69E-04	4.39E-08
	S1844	3	1	2	3	67031572	67365561	333.99	554	1.81E-05	1.00E-01	8.69E-04	4.39E-08
	S1850	3	1	2	3	125266261	126925703	1659.443	2150	1.81E-05	1.00E-01	8.69E-04	4.39E-08
	S1862	3	1	2	5	1857754	2111239	253.486	424	1.81E-05	1.00E-01	8.69E-04	4.39E-08
	S1866	3	1	2	5	34752123	34893413	141.291	161	1.81E-05	1.00E-01	8.69E-04	4.39E-08

	S1869	3	1	2	5	38128221	38310106	181.886	226	1.81E-05	1.00E-01	8.69E-04	4.39E-08
	S1877	3	1	2	6	9218232	9988810	770.579	735	1.81E-05	1.00E-01	8.69E-04	4.39E-08
	S1883	3	1	2	6	46070087	46367432	297.346	256	1.81E-05	1.00E-01	8.69E-04	4.39E-08
	S1890	3	1	2	6	162333574	163245816	912.243	1217	1.81E-05	1.00E-01	8.69E-04	4.39E-08
	S1900	3	1	2	7	156271913	157849514	1577.602	2437	1.81E-05	1.00E-01	8.69E-04	4.39E-08
	S1902	3	1	2	8	19507372	19634163	126.792	171	1.81E-05	1.00E-01	8.69E-04	4.39E-08
	S1908	3	1	2	8	133775165	134958276	1183.112	1395	1.81E-05	1.00E-01	8.69E-04	4.39E-08
	S1909	3	1	2	8	135282086	135729689	447.604	409	1.81E-05	1.00E-01	8.69E-04	4.39E-08
	S1910	3	1	2	8	142435107	143232207	797.101	1087	1.81E-05	1.00E-01	8.69E-04	4.39E-08
	S1911	3	1	2	9	23185204	24689761	1504.558	1391	1.81E-05	1.00E-01	8.69E-04	4.39E-08
	S1918	3	1	2	9	127861807	128395939	534.133	584	1.81E-05	1.00E-01	8.69E-04	4.39E-08
	S1927	3	1	2	11	112348647	112831159	482.513	528	1.81E-05	1.00E-01	8.69E-04	4.39E-08
	S1950	3	1	2	17	19986129	20229581	243.453	275	1.81E-05	1.00E-01	8.69E-04	4.39E-08
	S1952	3	1	2	18	29163891	29419557	255.667	230	1.81E-05	1.00E-01	8.69E-04	4.39E-08

	S1959	2	1	1	1	29373186	29802640	429.455	627	1.18E-07	1.00E-01	4.34E-04	2.86E-10
	S1967	2	1	1	1	229709434	230647630	938.197	1143	1.18E-07	1.00E-01	4.34E-04	2.86E-10
	S1968	2	1	1	1	244488897	244753806	264.91	210	1.18E-07	1.00E-01	4.34E-04	2.86E-10
	S1969	2	1	1	2	322579	1778691	1456.113	2035	1.18E-07	1.00E-01	4.34E-04	2.86E-10
	S1985	2	1	1	3	171456424	171854880	398.457	416	1.18E-07	1.00E-01	4.34E-04	2.86E-10
	S1989	2	1	1	4	94971560	95242732	271.173	261	1.18E-07	1.00E-01	4.34E-04	2.86E-10
	S1995	2	1	1	5	6293401	8055735	1762.335	2205	1.18E-07	1.00E-01	4.34E-04	2.86E-10
	S1998	2	1	1	5	32869565	33719331	849.767	996	1.18E-07	1.00E-01	4.34E-04	2.86E-10
	S2004	2	1	1	6	1846822	3048454	1201.633	1609	1.18E-07	1.00E-01	4.34E-04	2.86E-10
	S2007	2	1	1	6	138110216	138837055	726.84	793	1.18E-07	1.00E-01	4.34E-04	2.86E-10
	S2013	2	1	1	7	68164301	68347681	183.381	292	1.18E-07	1.00E-01	4.34E-04	2.86E-10
	S2021	2	1	1	8	1478333	1749240	270.908	474	1.18E-07	1.00E-01	4.34E-04	2.86E-10
	S2027	2	1	1	9	20527697	21940018	1412.322	1990	1.18E-07	1.00E-01	4.34E-04	2.86E-10
	S2028	2	1	1	9	85747002	86611354	864.353	699	1.18E-07	1.00E-01	4.34E-04	2.86E-10

	S2034	2	1	1	10	54527929	55092722	564.794	779	1.18E-07	1.00E-01	4.34E-04	2.86E-10
	S2036	2	1	1	11	111289904	111998826	708.923	489	1.18E-07	1.00E-01	4.34E-04	2.86E-10
	S2047	2	1	1	14	71134362	71755168	620.807	483	1.18E-07	1.00E-01	4.34E-04	2.86E-10
	S2063	2	1	1	19	33673118	34338451	665.334	647	1.18E-07	1.00E-01	4.34E-04	2.86E-10
	S2065	2	2	0	19	45042304	46610987	1568.684	1941	3.74E-58	2.00E-01	0.00E+00	9.07E-61
	S2066	2	2	0	21	16643809	17950673	1306.865	1830	3.74E-58	2.00E-01	0.00E+00	9.07E-61
	S2068	2	1	1	21	36541167	36645075	103.909	121	1.18E-07	1.00E-01	4.34E-04	2.86E-10
	S568	107	7	100	3	163220382	163432481	212.1	401	8.67E-08	9.33E-02	1.33E-02	5.82E-11
	S598	103	7	96	3	162726490	162941917	215.428	241	3.94E-08	9.33E-02	1.28E-02	2.64E-11
	S717	86	7	79	4	127010622	127332691	322.07	463	6.03E-10	9.33E-02	1.05E-02	4.04E-13
**EUR**	S718	86	7	79	4	127342147	127470436	128.29	122	6.03E-10	9.33E-02	1.05E-02	4.04E-13
	S732	83	6	77	1	45483005	46104019	621.015	973	1.82E-07	8.00E-02	1.03E-02	1.22E-10
	S735	82	6	76	4	150376254	150861537	485.284	611	1.44E-07	8.00E-02	1.01E-02	9.67E-11
	S788	75	8	67	3	136228302	136957860	729.559	999	2.31E-15	1.07E-01	8.93E-03	1.55E-18

	S796	75	7	68	10	103044198	103193308	149.111	260	1.47E-11	9.33E-02	9.06E-03	9.86E-15
	S841	70	10	60	16	47075338	47946642	871.305	378	1.20E-26	1.33E-01	7.99E-03	8.02E-30
	S843	69	7	62	7	118321110	118510547	189.438	249	1.18E-12	9.33E-02	8.26E-03	7.90E-16
	S881	64	6	58	1	92419450	93018831	599.382	859	6.73E-10	8.00E-02	7.73E-03	4.51E-13
	S900	63	5	58	11	66581032	66906236	325.205	227	7.23E-07	6.67E-02	7.73E-03	4.85E-10
	S933	61	8	53	5	60612700	61170442	557.743	801	3.33E-19	1.07E-01	7.06E-03	2.23E-22
	S953	59	5	54	3	161193918	161461944	268.027	332	2.32E-07	6.67E-02	7.20E-03	1.56E-10
	S960	59	5	54	5	88208666	88354818	146.153	160	2.32E-07	6.67E-02	7.20E-03	1.56E-10
	S970	58	5	53	3	160982708	161090052	107.345	163	1.71E-07	6.67E-02	7.06E-03	1.15E-10
	S1036	55	6	49	5	101986791	102198676	211.886	499	1.23E-11	8.00E-02	6.53E-03	8.28E-15
	S1057	54	6	48	5	102250985	102534752	283.768	580	7.30E-12	8.00E-02	6.40E-03	4.89E-15
	S1077	53	5	48	5	41674753	42265491	590.739	748	3.08E-08	6.67E-02	6.40E-03	2.07E-11
	S1089	53	5	48	11	28030407	28216895	186.489	180	3.08E-08	6.67E-02	6.40E-03	2.07E-11
	S1097	52	5	47	2	99249653	99525207	275.555	417	2.10E-08	6.67E-02	6.26E-03	1.41E-11

	S1105	52	5	47	5	42427878	42569483	141.606	200	2.10E-08	6.67E-02	6.26E-03	1.41E-11
	S1106	52	5	47	5	42670202	42814715	144.514	240	2.10E-08	6.67E-02	6.26E-03	1.41E-11
	S1117	51	5	46	2	98943776	99126956	183.181	227	1.42E-08	6.67E-02	6.13E-03	9.49E-12
	S1135	50	5	45	3	160436998	160601723	164.726	176	9.37E-09	6.67E-02	6.00E-03	6.28E-12
	S1154	49	5	44	5	51763217	51878223	115.007	187	6.10E-09	6.67E-02	5.86E-03	4.09E-12
	S1161	49	4	45	11	57753377	57897864	144.488	150	1.27E-05	5.33E-02	6.00E-03	8.49E-09
	S1178	48	8	40	5	85531640	85871407	339.768	701	8.93E-25	1.07E-01	5.33E-03	5.99E-28
	S1195	47	4	43	2	57393323	57618261	224.939	632	7.24E-06	5.33E-02	5.73E-03	4.85E-09
	S1200	47	5	42	3	158089171	158580216	491.046	1021	2.45E-09	6.67E-02	5.60E-03	1.64E-12
	S1237	46	7	39	15	72670982	72858511	187.53	280	1.68E-19	9.33E-02	5.20E-03	1.13E-22
	S1269	44	5	39	4	115601444	115708695	107.252	145	5.33E-10	6.67E-02	5.20E-03	3.58E-13
	S1283	44	6	38	9	99536384	99873516	337.133	210	1.02E-14	8.00E-02	5.06E-03	6.85E-18
	S1297	43	5	38	5	144360132	144654828	294.697	415	3.06E-10	6.67E-02	5.06E-03	2.05E-13
	S1298	43	5	38	5	144825397	145199225	373.829	732	3.06E-10	6.67E-02	5.06E-03	2.05E-13

	S1327	42	5	37	5	99075682	99206934	131.253	240	1.71E-10	6.67E-02	4.93E-03	1.15E-13
	S1330	42	5	37	6	35138422	35604002	465.581	599	1.71E-10	6.67E-02	4.93E-03	1.15E-13
	S1342	42	7	35	15	82531989	83265360	733.372	1071	1.87E-21	9.33E-02	4.66E-03	1.26E-24
	S1354	41	6	35	5	99997761	100510473	512.713	658	7.63E-16	8.00E-02	4.66E-03	5.12E-19
	S1360	41	5	36	7	86122679	86256197	133.519	248	9.30E-11	6.67E-02	4.80E-03	6.24E-14
	S1391	40	4	36	4	131500825	131711689	210.865	431	6.63E-07	5.33E-02	4.80E-03	4.44E-10
	S1395	40	4	36	5	101043619	101234176	190.558	276	6.63E-07	5.33E-02	4.80E-03	4.44E-10
	S1421	39	5	34	5	100635457	100804169	168.713	239	2.50E-11	6.67E-02	4.53E-03	1.68E-14
	S1422	39	4	35	5	100875291	100980866	105.576	117	4.39E-07	5.33E-02	4.66E-03	2.95E-10
	S1442	39	4	35	16	70449053	70618451	169.399	169	4.39E-07	5.33E-02	4.66E-03	2.95E-10
	S1448	38	4	34	2	57049020	57152377	103.358	229	2.85E-07	5.33E-02	4.53E-03	1.91E-10
	S1455	38	5	33	4	116822583	116933293	110.711	225	1.23E-11	6.67E-02	4.40E-03	8.28E-15
	S1458	38	4	34	4	131025609	131262485	236.877	382	2.85E-07	5.33E-02	4.53E-03	1.91E-10
	S1491	37	6	31	5	98403473	98608014	204.542	405	1.25E-17	8.00E-02	4.13E-03	8.39E-21

	S1506	37	6	31	10	105550827	105729875	179.049	317	1.25E-17	8.00E-02	4.13E-03	8.39E-21
	S1512	37	4	33	13	88983387	89158806	175.42	172	1.81E-07	5.33E-02	4.40E-03	1.21E-10
	S1528	36	4	32	5	40748166	40896378	148.213	299	1.12E-07	5.33E-02	4.26E-03	7.50E-11
	S1535	36	5	31	5	152961976	153068546	106.571	183	2.67E-12	6.67E-02	4.13E-03	1.79E-15
	S1559	36	4	32	14	64147430	64414763	267.334	444	1.12E-07	5.33E-02	4.26E-03	7.50E-11
	S1578	35	4	31	5	40351325	40562747	211.423	424	6.73E-08	5.33E-02	4.13E-03	4.51E-11
	S1599	35	5	30	11	105245498	105399022	153.525	214	1.16E-12	6.67E-02	4.00E-03	7.78E-16
	S1615	34	4	30	5	40107100	40297252	190.153	515	3.93E-08	5.33E-02	4.00E-03	2.64E-11
	S1626	34	6	28	7	126199359	126319192	119.834	218	3.04E-19	8.00E-02	3.73E-03	2.04E-22
	S1639	34	5	29	11	104830020	104930680	100.661	107	4.81E-13	6.67E-02	3.86E-03	3.23E-16
	S1648	34	4	30	14	45875744	46200129	324.386	640	3.93E-08	5.33E-02	4.00E-03	2.64E-11
	S1649	34	5	29	14	64443730	64598978	155.249	224	4.81E-13	6.67E-02	3.86E-03	3.23E-16
	S1650	34	4	30	15	50918936	51207300	288.365	506	3.93E-08	5.33E-02	4.00E-03	2.64E-11
	S1651	34	8	26	15	64144763	64757158	612.396	768	1.58E-35	1.07E-01	3.46E-03	1.06E-38

	S1659	33	4	29	1	75049336	75362848	313.513	512	2.22E-08	5.33E-02	3.86E-03	1.49E-11
	S1660	33	6	27	1	102752294	103347535	595.242	1608	7.60E-20	8.00E-02	3.60E-03	5.10E-23
	S1661	33	6	27	1	103347850	103497405	149.556	182	7.60E-20	8.00E-02	3.60E-03	5.10E-23
	S1693	33	5	28	9	124806523	125988959	1182.437	1276	1.89E-13	6.67E-02	3.73E-03	1.27E-16
	S1752	32	5	27	9	30173292	30422187	248.896	402	7.02E-14	6.67E-02	3.60E-03	4.71E-17
	S1763	32	4	28	14	39253478	39418472	164.995	339	1.21E-08	5.33E-02	3.73E-03	8.15E-12
	S1764	32	4	28	14	39532707	39719604	186.898	400	1.21E-08	5.33E-02	3.73E-03	8.15E-12
	S1786	31	6	25	1	96479319	96599318	120	208	3.62E-21	8.00E-02	3.33E-03	2.43E-24
	S1789	31	4	27	2	54392335	54538546	146.212	292	6.38E-09	5.33E-02	3.60E-03	4.28E-12
	S1798	31	6	25	3	137793741	137902489	108.749	199	3.62E-21	8.00E-02	3.33E-03	2.43E-24
	S1809	31	4	27	5	86884740	87001969	117.23	209	6.38E-09	5.33E-02	3.60E-03	4.28E-12
	S1831	31	4	27	12	40266815	40421822	155.008	387	6.38E-09	5.33E-02	3.60E-03	4.28E-12
	S1848	31	5	26	22	31219664	31428273	208.61	293	2.45E-14	6.67E-02	3.46E-03	1.64E-17
	S1854	30	6	24	1	96628173	96819837	191.665	419	6.79E-22	8.00E-02	3.20E-03	4.55E-25

	S1870	30	4	26	3	34870090	34978458	108.369	265	3.21E-09	5.33E-02	3.46E-03	2.16E-12
	S1872	30	5	25	3	159760961	159879492	118.532	192	7.94E-15	6.67E-02	3.33E-03	5.32E-18
	S1886	30	6	24	5	119032102	119285329	253.228	323	6.79E-22	8.00E-02	3.20E-03	4.55E-25
	S1914	30	7	23	16	29991755	30108956	117.202	121	1.98E-30	9.33E-02	3.06E-03	1.33E-33
	S1937	29	4	25	2	103834512	104099228	264.717	440	1.54E-09	5.33E-02	3.33E-03	1.04E-12
	S1959	29	4	25	4	147148704	147327594	178.891	191	1.54E-09	5.33E-02	3.33E-03	1.04E-12
	S1968	29	3	26	6	68961693	69223908	262.216	558	3.18E-05	4.00E-02	3.46E-03	2.13E-08
	S1971	29	4	25	7	87944332	88048209	103.878	103	1.54E-09	5.33E-02	3.33E-03	1.04E-12
	S1981	29	8	21	10	99630143	99870954	240.812	336	7.04E-42	1.07E-01	2.80E-03	4.72E-45
	S1984	29	4	25	12	41120483	41284161	163.679	295	1.54E-09	5.33E-02	3.33E-03	1.04E-12
	S2016	28	3	25	4	65440570	65654198	213.629	528	2.12E-05	4.00E-02	3.33E-03	1.42E-08
	S2028	28	7	21	5	118190909	118378925	188.017	373	1.13E-32	9.33E-02	2.80E-03	7.59E-36
	S2031	28	7	21	5	127800776	127991179	190.404	259	1.13E-32	9.33E-02	2.80E-03	7.59E-36
	S2043	28	3	25	8	66251491	66547961	296.471	450	2.12E-05	4.00E-02	3.33E-03	1.42E-08

	S2056	28	5	23	13	83363264	83515286	152.023	263	6.58E-16	6.67E-02	3.06E-03	4.42E-19
	S2068	27	3	24	1	152969457	153087421	117.965	209	1.37E-05	4.00E-02	3.20E-03	9.16E-09
	S2070	27	3	24	2	25710129	25841729	131.601	171	1.37E-05	4.00E-02	3.20E-03	9.16E-09
	S2073	27	3	24	2	80726281	80951884	225.604	300	1.37E-05	4.00E-02	3.20E-03	9.16E-09
	S2081	27	4	23	3	35625565	35765955	140.391	222	3.03E-10	5.33E-02	3.06E-03	2.03E-13
	S2083	27	3	24	3	153991255	154128535	137.281	322	1.37E-05	4.00E-02	3.20E-03	9.16E-09
	S2084	27	3	24	3	154707839	154856915	149.077	161	1.37E-05	4.00E-02	3.20E-03	9.16E-09
	S2090	27	3	24	4	120037024	120425543	388.52	687	1.37E-05	4.00E-02	3.20E-03	9.16E-09
	S2102	27	3	24	6	68477133	68580739	103.607	337	1.37E-05	4.00E-02	3.20E-03	9.16E-09
	S2112	27	4	23	9	70159361	70457170	297.81	615	3.03E-10	5.33E-02	3.06E-03	2.03E-13
	S2114	27	7	20	10	99295965	99494770	198.806	310	6.43E-34	9.33E-02	2.66E-03	4.31E-37
	S2140	26	4	22	2	73009827	73130477	120.651	163	1.22E-10	5.33E-02	2.93E-03	8.21E-14
	S2141	26	4	22	2	73136702	73261579	124.878	232	1.22E-10	5.33E-02	2.93E-03	8.21E-14
	S2142	26	3	23	2	73267667	73717963	450.297	529	8.53E-06	4.00E-02	3.06E-03	5.72E-09

	S2151	26	3	23	3	101504877	101748891	244.015	361	8.53E-06	4.00E-02	3.06E-03	5.72E-09
	S2153	26	7	19	3	138301308	138406810	105.503	131	2.93E-35	9.33E-02	2.53E-03	1.97E-38
	S2154	26	7	19	3	138417207	138607871	190.665	202	2.93E-35	9.33E-02	2.53E-03	1.97E-38
	S2156	26	3	23	3	154867643	154995835	128.193	149	8.53E-06	4.00E-02	3.06E-03	5.72E-09
	S2160	26	5	21	4	105103424	105382725	279.302	449	3.73E-17	6.67E-02	2.80E-03	2.50E-20
	S2162	26	5	21	4	105586876	105871223	284.348	445	3.73E-17	6.67E-02	2.80E-03	2.50E-20
	S2165	26	3	23	4	146090945	146242957	152.013	202	8.53E-06	4.00E-02	3.06E-03	5.72E-09
	S2168	26	4	22	4	158052381	158170954	118.574	136	1.22E-10	5.33E-02	2.93E-03	8.21E-14
	S2179	26	5	21	7	89508645	89645391	136.747	146	3.73E-17	6.67E-02	2.80E-03	2.50E-20
	S2181	26	6	20	7	117312687	117559590	246.904	274	2.33E-25	8.00E-02	2.66E-03	1.56E-28
	S2193	26	4	22	12	43309248	43445404	136.157	247	1.22E-10	5.33E-02	2.93E-03	8.21E-14
	S2213	25	3	22	1	77614023	78049412	435.39	719	5.13E-06	4.00E-02	2.93E-03	3.44E-09
	S2224	25	3	22	2	109483602	109664198	180.597	338	5.13E-06	4.00E-02	2.93E-03	3.44E-09
	S2237	25	4	21	3	120945746	121049185	103.44	253	4.60E-11	5.33E-02	2.80E-03	3.09E-14

	S2257	25	4	21	5	91501022	91648736	147.715	186	4.60E-11	5.33E-02	2.80E-03	3.09E-14
	S2273	25	5	20	7	110843621	111219777	376.157	380	7.48E-18	6.67E-02	2.66E-03	5.02E-21
	S2302	25	3	22	14	46742130	46908844	166.715	229	5.13E-06	4.00E-02	2.93E-03	3.44E-09
	S2305	25	7	18	15	84946006	85497641	551.636	973	1.05E-36	9.33E-02	2.40E-03	7.01E-40
	S2308	25	3	22	20	37063522	37263747	200.226	282	5.13E-06	4.00E-02	2.93E-03	3.44E-09
	S2331	24	3	21	4	66844732	67033166	188.435	370	2.96E-06	4.00E-02	2.80E-03	1.99E-09
	S2332	24	3	21	4	67192689	67325161	132.473	298	2.96E-06	4.00E-02	2.80E-03	1.99E-09
	S2337	24	5	19	4	106121378	106377212	255.835	360	1.31E-18	6.67E-02	2.53E-03	8.80E-22
	S2342	24	4	20	4	159954848	160105947	151.1	267	1.59E-11	5.33E-02	2.66E-03	1.07E-14
	S2345	24	4	20	5	64176099	64439914	263.816	341	1.59E-11	5.33E-02	2.66E-03	1.07E-14
	S2361	24	4	20	7	88659195	88881919	222.725	402	1.59E-11	5.33E-02	2.66E-03	1.07E-14
	S2386	24	4	20	11	106098603	106381264	282.662	399	1.59E-11	5.33E-02	2.66E-03	1.07E-14
	S2391	24	3	21	12	123582733	123773596	190.864	255	2.96E-06	4.00E-02	2.80E-03	1.99E-09
	S2411	23	3	20	1	79194804	79428138	233.335	513	1.63E-06	4.00E-02	2.66E-03	1.09E-09

	S2413	23	3	20	1	153240271	153357702	117.432	229	1.63E-06	4.00E-02	2.66E-03	1.09E-09
	S2439	23	5	18	3	152349004	152562959	213.956	343	1.98E-19	6.67E-02	2.40E-03	1.33E-22
	S2444	23	5	18	3	180804790	181161985	357.196	444	1.98E-19	6.67E-02	2.40E-03	1.33E-22
	S2448	23	5	18	4	106595388	106696952	101.565	188	1.98E-19	6.67E-02	2.40E-03	1.33E-22
	S2449	23	4	19	4	107712350	107830649	118.3	159	5.04E-12	5.33E-02	2.53E-03	3.38E-15
	S2459	23	3	20	5	63407384	63589089	181.706	462	1.63E-06	4.00E-02	2.66E-03	1.09E-09
	S2468	23	4	19	6	71358447	71667230	308.784	582	5.04E-12	5.33E-02	2.53E-03	3.38E-15
	S2478	23	5	18	7	90001843	91005210	1003.368	2238	1.98E-19	6.67E-02	2.40E-03	1.33E-22
	S2479	23	3	20	7	102769649	103149134	379.486	426	1.63E-06	4.00E-02	2.66E-03	1.09E-09
	S2487	23	5	18	9	29388763	29511848	123.086	338	1.98E-19	6.67E-02	2.40E-03	1.33E-22
	S2489	23	5	18	9	96519987	96842892	322.906	188	1.98E-19	6.67E-02	2.40E-03	1.33E-22
	S2499	23	3	20	11	59339302	59855905	516.604	577	1.63E-06	4.00E-02	2.66E-03	1.09E-09
	S2501	23	5	18	12	18153827	18355318	201.492	412	1.98E-19	6.67E-02	2.40E-03	1.33E-22
	S2503	23	4	19	12	41615371	41820036	204.666	523	5.04E-12	5.33E-02	2.53E-03	3.38E-15

	S2521	23	4	19	18	22650558	22800302	149.745	228	5.04E-12	5.33E-02	2.53E-03	3.38E-15
	S2522	23	4	19	18	23494486	23725402	230.917	239	5.04E-12	5.33E-02	2.53E-03	3.38E-15
	S2533	22	4	18	1	118756334	119181912	425.579	881	1.44E-12	5.33E-02	2.40E-03	9.63E-16
	S2534	22	4	18	1	155061442	155464151	402.71	432	1.44E-12	5.33E-02	2.40E-03	9.63E-16
	S2551	22	4	18	3	36064241	36313019	248.779	441	1.44E-12	5.33E-02	2.40E-03	9.63E-16
	S2556	22	7	15	3	139405330	139577110	171.781	309	7.71E-42	9.33E-02	2.00E-03	5.17E-45
	S2560	22	4	18	4	53010350	53351440	341.091	570	1.44E-12	5.33E-02	2.40E-03	9.63E-16
	S2578	22	4	18	5	94036860	94209819	172.96	153	1.44E-12	5.33E-02	2.40E-03	9.63E-16
	S2579	22	6	16	5	104033089	104163108	130.02	291	4.44E-30	8.00E-02	2.13E-03	2.98E-33
	S2580	22	6	16	5	114916898	115105269	188.372	346	4.44E-30	8.00E-02	2.13E-03	2.98E-33
	S2582	22	4	18	6	71744101	71857261	113.161	310	1.44E-12	5.33E-02	2.40E-03	9.63E-16
	S2593	22	5	17	9	94129286	94646689	517.404	1087	2.52E-20	6.67E-02	2.27E-03	1.69E-23
	S2611	22	3	19	13	53482298	53615104	132.807	243	8.51E-07	4.00E-02	2.53E-03	5.71E-10
	S2623	22	3	19	20	24730591	25039695	309.105	639	8.51E-07	4.00E-02	2.53E-03	5.71E-10

	S2628	21	6	15	1	94921350	95089873	168.524	438	1.54E-31	8.00E-02	2.00E-03	1.03E-34
	S2630	21	3	18	1	186052504	186179685	127.182	189	4.18E-07	4.00E-02	2.40E-03	2.80E-10
	S2643	21	4	17	2	166340163	166498891	158.729	302	3.64E-13	5.33E-02	2.27E-03	2.44E-16
	S2650	21	4	17	3	120551674	120659251	107.578	219	3.64E-13	5.33E-02	2.27E-03	2.44E-16
	S2654	21	4	17	3	155360686	155879249	518.564	815	3.64E-13	5.33E-02	2.27E-03	2.44E-16
	S2663	21	3	18	4	104009571	104303404	293.834	406	4.18E-07	4.00E-02	2.40E-03	2.80E-10
	S2674	21	5	16	5	62230173	62704338	474.166	731	2.64E-21	6.67E-02	2.13E-03	1.77E-24
	S2675	21	4	17	5	64771729	65093506	321.778	439	3.64E-13	5.33E-02	2.27E-03	2.44E-16
	S2679	21	4	17	5	92788688	92914109	125.422	162	3.64E-13	5.33E-02	2.27E-03	2.44E-16
	S2683	21	3	18	6	66695187	66845333	150.147	444	4.18E-07	4.00E-02	2.40E-03	2.80E-10
	S2698	21	3	18	7	53846990	54420876	573.887	1172	4.18E-07	4.00E-02	2.40E-03	2.80E-10
	S2699	21	3	18	7	54442337	54556182	113.846	263	4.18E-07	4.00E-02	2.40E-03	2.80E-10
	S2726	21	4	17	11	104173698	104319001	145.304	274	3.64E-13	5.33E-02	2.27E-03	2.44E-16
	S2728	21	4	17	12	113172684	113337273	164.59	205	3.64E-13	5.33E-02	2.27E-03	2.44E-16

	S2730	21	3	18	13	51851960	52727001	875.042	797	4.18E-07	4.00E-02	2.40E-03	2.80E-10
	S2733	21	3	18	14	48258630	48514048	255.419	395	4.18E-07	4.00E-02	2.40E-03	2.80E-10
	S2750	20	5	15	1	69325182	69577300	252.119	475	2.20E-22	6.67E-02	2.00E-03	1.48E-25
	S2757	20	5	15	1	179090815	179502350	411.536	1109	2.20E-22	6.67E-02	2.00E-03	1.48E-25
	S2769	20	3	17	2	74088950	74200888	111.939	162	1.91E-07	4.00E-02	2.27E-03	1.28E-10
	S2770	20	3	17	2	74206825	74492875	286.051	245	1.91E-07	4.00E-02	2.27E-03	1.28E-10
	S2776	20	3	17	2	109156091	109333040	176.95	287	1.91E-07	4.00E-02	2.27E-03	1.28E-10
	S2786	20	3	17	3	156019099	156180200	161.102	217	1.91E-07	4.00E-02	2.27E-03	1.28E-10
	S2792	20	3	17	4	63117518	63239728	122.211	359	1.91E-07	4.00E-02	2.27E-03	1.28E-10
	S2797	20	3	17	4	103076526	103461086	384.561	517	1.91E-07	4.00E-02	2.27E-03	1.28E-10
	S2798	20	5	15	4	108215568	108628351	412.784	690	2.20E-22	6.67E-02	2.00E-03	1.48E-25
	S2814	20	6	14	5	120723494	121037190	313.697	610	3.81E-33	8.00E-02	1.87E-03	2.56E-36
	S2834	20	6	14	7	116675151	116807803	132.653	141	3.81E-33	8.00E-02	1.87E-03	2.56E-36
	S2841	20	5	15	9	97417559	97519530	101.972	116	2.20E-22	6.67E-02	2.00E-03	1.48E-25

	S2844	20	4	16	10	87024733	87139423	114.691	198	8.03E-14	5.33E-02	2.13E-03	5.38E-17
	S2854	20	5	15	11	81943048	82276048	333.001	754	2.20E-22	6.67E-02	2.00E-03	1.48E-25
	S2855	20	3	17	11	83322955	83767909	444.955	674	1.91E-07	4.00E-02	2.27E-03	1.28E-10
	S2878	20	5	15	20	38504150	39239740	735.591	1173	2.20E-22	6.67E-02	2.00E-03	1.48E-25
	S2888	19	3	16	1	186881247	186999579	118.333	305	8.07E-08	4.00E-02	2.13E-03	5.41E-11
	S2892	19	3	16	2	40045179	40199527	154.349	418	8.07E-08	4.00E-02	2.13E-03	5.41E-11
	S2894	19	3	16	2	51800133	51960015	159.883	341	8.07E-08	4.00E-02	2.13E-03	5.41E-11
	S2895	19	5	14	2	84608215	84912339	304.125	406	1.42E-23	6.67E-02	1.87E-03	9.51E-27
	S2904	19	5	14	3	146215990	146366672	150.683	232	1.42E-23	6.67E-02	1.87E-03	9.51E-27
	S2907	19	3	16	4	72020257	72135818	115.562	107	8.07E-08	4.00E-02	2.13E-03	5.41E-11
	S2908	19	3	16	4	72989406	73124906	135.501	112	8.07E-08	4.00E-02	2.13E-03	5.41E-11
	S2909	19	3	16	4	79023203	79273570	250.368	351	8.07E-08	4.00E-02	2.13E-03	5.41E-11
	S2910	19	3	16	4	79275415	79408348	132.934	156	8.07E-08	4.00E-02	2.13E-03	5.41E-11
	S2911	19	3	16	4	79640216	79753497	113.282	263	8.07E-08	4.00E-02	2.13E-03	5.41E-11

	S2912	19	3	16	4	79896824	80058292	161.469	295	8.07E-08	4.00E-02	2.13E-03	5.41E-11
	S2915	19	3	16	4	102299244	102467856	168.613	321	8.07E-08	4.00E-02	2.13E-03	5.41E-11
	S2919	19	3	16	4	120862858	121050058	187.201	405	8.07E-08	4.00E-02	2.13E-03	5.41E-11
	S2920	19	4	15	4	122203821	122337115	133.295	153	1.51E-14	5.33E-02	2.00E-03	1.02E-17
	S2921	19	4	15	4	122362008	122684588	322.581	557	1.51E-14	5.33E-02	2.00E-03	1.02E-17
	S2928	19	4	15	4	160668218	160879898	211.681	521	1.51E-14	5.33E-02	2.00E-03	1.02E-17
	S2938	19	5	14	5	94465772	94860050	394.279	592	1.42E-23	6.67E-02	1.87E-03	9.51E-27
	S2941	19	6	13	5	103842705	103979003	136.299	270	6.41E-35	8.00E-02	1.73E-03	4.30E-38
	S2960	19	4	15	7	113297046	113698834	401.789	580	1.51E-14	5.33E-02	2.00E-03	1.02E-17
	S2965	19	3	16	8	74532571	74663290	130.72	218	8.07E-08	4.00E-02	2.13E-03	5.41E-11
	S2969	19	5	14	9	103206390	103481476	275.087	457	1.42E-23	6.67E-02	1.87E-03	9.51E-27
	S2975	19	4	15	10	87393376	87709196	315.821	433	1.51E-14	5.33E-02	2.00E-03	1.02E-17
	S2979	19	6	13	10	110444589	110589446	144.858	235	6.41E-35	8.00E-02	1.73E-03	4.30E-38
	S2983	19	4	15	11	63987039	64108085	121.047	177	1.51E-14	5.33E-02	2.00E-03	1.02E-17

	S2995	19	5	14	13	90027560	90164304	136.745	433	1.42E-23	6.67E-02	1.87E-03	9.51E-27
	S2999	19	4	15	14	43208865	43440539	231.675	548	1.51E-14	5.33E-02	2.00E-03	1.02E-17
	S3011	19	3	16	20	21570020	22519928	949.909	1213	8.07E-08	4.00E-02	2.13E-03	5.41E-11
	S3015	18	4	14	1	88424691	89054547	629.857	1044	2.37E-15	5.33E-02	1.87E-03	1.59E-18
	S3021	18	3	15	1	153455093	153583772	128.68	206	3.10E-08	4.00E-02	2.00E-03	2.08E-11
	S3041	18	3	15	3	37699926	37905377	205.452	277	3.10E-08	4.00E-02	2.00E-03	2.08E-11
	S3050	18	3	15	4	77791086	77960750	169.665	226	3.10E-08	4.00E-02	2.00E-03	2.08E-11
	S3053	18	4	14	4	101802162	102103230	301.069	444	2.37E-15	5.33E-02	1.87E-03	1.59E-18
	S3058	18	4	14	4	123892694	124489142	596.449	1049	2.37E-15	5.33E-02	1.87E-03	1.59E-18
	S3059	18	4	14	4	129270221	129410251	140.031	267	2.37E-15	5.33E-02	1.87E-03	1.59E-18
	S3065	18	3	15	5	36470689	36595701	125.013	266	3.10E-08	4.00E-02	2.00E-03	2.08E-11
	S3068	18	6	12	5	113485440	113767881	282.442	766	6.85E-37	8.00E-02	1.60E-03	4.59E-40
	S3081	18	5	13	7	116386431	116522675	136.245	251	6.74E-25	6.67E-02	1.73E-03	4.52E-28
	S3086	18	3	15	8	93417861	93603344	185.484	223	3.10E-08	4.00E-02	2.00E-03	2.08E-11

	S3094	18	5	13	9	97854739	98082725	227.987	352	6.74E-25	6.67E-02	1.73E-03	4.52E-28
	S3104	18	3	15	11	43031345	43254070	222.726	652	3.10E-08	4.00E-02	2.00E-03	2.08E-11
	S3110	18	3	15	12	49348706	49543609	194.904	237	3.10E-08	4.00E-02	2.00E-03	2.08E-11
	S3113	18	3	15	13	51524383	51626868	102.486	100	3.10E-08	4.00E-02	2.00E-03	2.08E-11
	S3115	18	3	15	13	80375230	80586775	211.546	357	3.10E-08	4.00E-02	2.00E-03	2.08E-11
	S3117	18	3	15	13	85579280	85738495	159.216	343	3.10E-08	4.00E-02	2.00E-03	2.08E-11
	S3121	18	5	13	16	14382126	14861506	479.381	301	6.74E-25	6.67E-02	1.73E-03	4.52E-28
	S3122	18	5	13	16	15304078	15554459	250.382	259	6.74E-25	6.67E-02	1.73E-03	4.52E-28
	S3138	17	7	10	1	98266257	98474702	208.446	423	2.08E-54	9.33E-02	1.33E-03	1.39E-57
	S3144	17	3	14	2	19889650	20178444	288.795	564	1.06E-08	4.00E-02	1.87E-03	7.13E-12
	S3146	17	4	13	2	35888056	36141118	253.063	612	3.00E-16	5.33E-02	1.73E-03	2.01E-19
	S3152	17	3	14	2	106142889	106337831	194.943	335	1.06E-08	4.00E-02	1.87E-03	7.13E-12
	S3153	17	3	14	2	106520001	106943651	423.651	925	1.06E-08	4.00E-02	1.87E-03	7.13E-12
	S3163	17	3	14	2	181324823	181446867	122.045	189	1.06E-08	4.00E-02	1.87E-03	7.13E-12

	S3166	17	4	13	3	36817092	37404889	587.798	667	3.00E-16	5.33E-02	1.73E-03	2.01E-19
	S3167	17	4	13	3	37418996	37569129	150.134	288	3.00E-16	5.33E-02	1.73E-03	2.01E-19
	S3168	17	4	13	3	39378730	39561173	182.444	286	3.00E-16	5.33E-02	1.73E-03	2.01E-19
	S3169	17	5	12	3	39932537	40150764	218.228	330	2.24E-26	6.67E-02	1.60E-03	1.50E-29
	S3174	17	3	14	3	111175609	111535059	359.451	599	1.06E-08	4.00E-02	1.87E-03	7.13E-12
	S3179	17	3	14	3	130583509	130789857	206.349	228	1.06E-08	4.00E-02	1.87E-03	7.13E-12
	S3183	17	4	13	4	62816824	62929036	112.213	188	3.00E-16	5.33E-02	1.73E-03	2.01E-19
	S3200	17	3	14	4	156695073	156890211	195.139	309	1.06E-08	4.00E-02	1.87E-03	7.13E-12
	S3204	17	4	13	5	58044929	58254966	210.038	569	3.00E-16	5.33E-02	1.73E-03	2.01E-19
	S3219	17	4	13	7	52956080	53094947	138.868	372	3.00E-16	5.33E-02	1.73E-03	2.01E-19
	S3222	17	4	13	7	103569959	103718024	148.066	267	3.00E-16	5.33E-02	1.73E-03	2.01E-19
	S3228	17	4	13	9	73871927	74111519	239.593	384	3.00E-16	5.33E-02	1.73E-03	2.01E-19
	S3230	17	5	12	9	111073708	111928034	854.327	1828	2.24E-26	6.67E-02	1.60E-03	1.50E-29
	S3257	17	5	12	13	83745085	83895499	150.415	113	2.24E-26	6.67E-02	1.60E-03	1.50E-29

	S3262	17	3	14	13	85163328	85279952	116.625	264	1.06E-08	4.00E-02	1.87E-03	7.13E-12
	S3277	16	6	10	1	99883099	100255291	372.193	758	1.43E-41	8.00E-02	1.33E-03	9.61E-45
	S3279	16	5	11	1	100361534	100560437	198.904	273	4.88E-28	6.67E-02	1.47E-03	3.27E-31
	S3282	16	3	13	1	153809535	154158721	349.187	478	3.20E-09	4.00E-02	1.73E-03	2.15E-12
	S3283	16	3	13	1	154576516	154683482	106.967	179	3.20E-09	4.00E-02	1.73E-03	2.15E-12
	S3299	16	3	13	2	71490543	71667696	177.154	332	3.20E-09	4.00E-02	1.73E-03	2.15E-12
	S3306	16	3	13	2	181537230	181924539	387.31	427	3.20E-09	4.00E-02	1.73E-03	2.15E-12
	S3307	16	3	13	2	181996698	182300974	304.277	490	3.20E-09	4.00E-02	1.73E-03	2.15E-12
	S3321	16	3	13	3	110369920	110581278	211.359	356	3.20E-09	4.00E-02	1.73E-03	2.15E-12
	S3322	16	3	13	3	110802487	110952118	149.632	139	3.20E-09	4.00E-02	1.73E-03	2.15E-12
	S3330	16	3	13	4	75905987	76402625	496.639	974	3.20E-09	4.00E-02	1.73E-03	2.15E-12
	S3334	16	4	12	4	123205795	123317378	111.584	154	2.93E-17	5.33E-02	1.60E-03	1.96E-20
	S3335	16	4	12	4	124746308	124943442	197.135	485	2.93E-17	5.33E-02	1.60E-03	1.96E-20
	S3337	16	4	12	4	156031242	156229420	198.179	458	2.93E-17	5.33E-02	1.60E-03	1.96E-20

	S3353	16	4	12	7	103755459	103893018	137.56	337	2.93E-17	5.33E-02	1.60E-03	1.96E-20
	S3354	16	4	12	7	103951375	104082720	131.346	226	2.93E-17	5.33E-02	1.60E-03	1.96E-20
	S3359	16	4	12	7	113760374	113960428	200.055	234	2.93E-17	5.33E-02	1.60E-03	1.96E-20
	S3368	16	4	12	9	95446162	95554049	107.888	157	2.93E-17	5.33E-02	1.60E-03	1.96E-20
	S3370	16	6	10	9	108879247	109071512	192.266	421	1.43E-41	8.00E-02	1.33E-03	9.61E-45
	S3380	16	6	10	10	100833866	100999999	166.134	247	1.43E-41	8.00E-02	1.33E-03	9.61E-45
	S3389	16	5	11	12	31628444	31745841	117.398	202	4.88E-28	6.67E-02	1.47E-03	3.27E-31
	S3390	16	3	13	12	48390156	48566275	176.12	460	3.20E-09	4.00E-02	1.73E-03	2.15E-12
	S3413	16	4	12	20	37614458	37932599	318.142	470	2.93E-17	5.33E-02	1.60E-03	1.96E-20
	S3419	15	5	10	1	90760037	91024576	264.54	417	6.39E-30	6.67E-02	1.33E-03	4.28E-33
	S3423	15	5	10	1	111863560	111992930	129.371	296	6.39E-30	6.67E-02	1.33E-03	4.28E-33
	S3432	15	3	12	1	242651579	243624192	972.614	1472	8.22E-10	4.00E-02	1.60E-03	5.52E-13
	S3438	15	3	12	2	40399996	40841566	441.571	770	8.22E-10	4.00E-02	1.60E-03	5.52E-13
	S3443	15	4	11	2	76427435	76694126	266.692	646	2.10E-18	5.33E-02	1.47E-03	1.41E-21

	S3444	15	4	11	2	85290783	85447453	156.671	305	2.10E-18	5.33E-02	1.47E-03	1.41E-21
	S3445	15	3	12	2	85838133	86096216	258.084	412	8.22E-10	4.00E-02	1.60E-03	5.52E-13
	S3446	15	3	12	2	86332262	86438385	106.124	154	8.22E-10	4.00E-02	1.60E-03	5.52E-13
	S3454	15	3	12	3	22533607	23728406	1194.8	2487	8.22E-10	4.00E-02	1.60E-03	5.52E-13
	S3457	15	4	11	3	40194610	40669866	475.257	501	2.10E-18	5.33E-02	1.47E-03	1.41E-21
	S3462	15	6	9	3	134919546	135037148	117.603	244	2.24E-44	8.00E-02	1.20E-03	1.50E-47
	S3465	15	5	10	3	140255544	140393506	137.963	213	6.39E-30	6.67E-02	1.33E-03	4.28E-33
	S3467	15	5	10	3	141877041	142051807	174.767	361	6.39E-30	6.67E-02	1.33E-03	4.28E-33
	S3468	15	5	10	3	144193170	144800114	606.945	887	6.39E-30	6.67E-02	1.33E-03	4.28E-33
	S3469	15	5	10	3	144937030	145178697	241.668	394	6.39E-30	6.67E-02	1.33E-03	4.28E-33
	S3470	15	5	10	3	178620339	178843865	223.527	499	6.39E-30	6.67E-02	1.33E-03	4.28E-33
	S3475	15	5	10	4	99414429	99721371	306.943	521	6.39E-30	6.67E-02	1.33E-03	4.28E-33
	S3476	15	5	10	4	100084122	100231322	147.201	310	6.39E-30	6.67E-02	1.33E-03	4.28E-33
	S3477	15	4	11	4	101398129	101602384	204.256	283	2.10E-18	5.33E-02	1.47E-03	1.41E-21

	S3478	15	4	11	4	101623076	101762340	139.265	325	2.10E-18	5.33E-02	1.47E-03	1.41E-21
	S3486	15	5	10	5	107971620	108258657	287.038	597	6.39E-30	6.67E-02	1.33E-03	4.28E-33
	S3487	15	5	10	5	108261691	109123256	861.566	1775	6.39E-30	6.67E-02	1.33E-03	4.28E-33
	S3488	15	6	9	5	109553994	109897792	343.799	793	2.24E-44	8.00E-02	1.20E-03	1.50E-47
	S3489	15	5	10	5	109899863	110016297	116.435	170	6.39E-30	6.67E-02	1.33E-03	4.28E-33
	S3490	15	6	9	5	110727539	111055212	327.674	428	2.24E-44	8.00E-02	1.20E-03	1.50E-47
	S3492	15	5	10	5	112201871	112637844	435.974	906	6.39E-30	6.67E-02	1.33E-03	4.28E-33
	S3495	15	5	10	5	116669616	116786594	116.979	218	6.39E-30	6.67E-02	1.33E-03	4.28E-33
	S3509	15	4	11	7	52724462	52880113	155.652	431	2.10E-18	5.33E-02	1.47E-03	1.41E-21
	S3512	15	4	11	7	68438460	68558144	119.685	260	2.10E-18	5.33E-02	1.47E-03	1.41E-21
	S3522	15	4	11	9	33187899	33318590	130.692	178	2.10E-18	5.33E-02	1.47E-03	1.41E-21
	S3527	15	5	10	9	102590939	102797485	206.547	595	6.39E-30	6.67E-02	1.33E-03	4.28E-33
	S3528	15	5	10	9	109282483	109419417	136.935	232	6.39E-30	6.67E-02	1.33E-03	4.28E-33
	S3533	15	7	8	10	106395543	106607970	212.428	325	8.87E-62	9.33E-02	1.07E-03	5.95E-65

	S3534	15	4	11	10	114971070	115134555	163.486	176	2.10E-18	5.33E-02	1.47E-03	1.41E-21
	S3535	15	5	10	11	26153726	26653514	499.789	1020	6.39E-30	6.67E-02	1.33E-03	4.28E-33
	S3536	15	3	12	11	40853581	41188057	334.477	567	8.22E-10	4.00E-02	1.60E-03	5.52E-13
	S3537	15	3	12	11	41393487	41701745	308.259	602	8.22E-10	4.00E-02	1.60E-03	5.52E-13
	S3546	15	3	12	12	30775783	30919706	143.924	324	8.22E-10	4.00E-02	1.60E-03	5.52E-13
	S3548	15	4	11	12	46545731	46736257	190.527	246	2.10E-18	5.33E-02	1.47E-03	1.41E-21
	S3554	15	3	12	13	94082159	94199614	117.456	227	8.22E-10	4.00E-02	1.60E-03	5.52E-13
	S3555	15	4	11	14	48886762	49111687	224.926	370	2.10E-18	5.33E-02	1.47E-03	1.41E-21
	S3556	15	4	11	14	49374173	49725908	351.736	439	2.10E-18	5.33E-02	1.47E-03	1.41E-21
	S3559	15	3	12	14	61958980	62176938	217.959	487	8.22E-10	4.00E-02	1.60E-03	5.52E-13
	S3560	15	3	12	14	71183965	71624711	440.747	540	8.22E-10	4.00E-02	1.60E-03	5.52E-13
	S3565	15	4	11	15	45143019	45253280	110.262	160	2.10E-18	5.33E-02	1.47E-03	1.41E-21
	S3566	15	3	12	16	20406378	20593795	187.418	189	8.22E-10	4.00E-02	1.60E-03	5.52E-13
	S3567	15	9	6	16	49626115	49842961	216.847	421	1.95E-105	1.20E-01	7.99E-04	1.31E-108

	S3568	15	9	6	16	49920928	50053087	132.16	221	1.95E-105	1.20E-01	7.99E-04	1.31E-108
	S3569	15	3	12	16	74896352	75123124	226.773	403	8.22E-10	4.00E-02	1.60E-03	5.52E-13
	S3581	15	3	12	20	40669156	40811614	142.459	372	8.22E-10	4.00E-02	1.60E-03	5.52E-13
	S3586	14	5	9	1	108368891	108651391	282.501	295	4.51E-32	6.67E-02	1.20E-03	3.02E-35
	S3587	14	4	10	1	112474673	112644156	169.484	324	1.04E-19	5.33E-02	1.33E-03	6.95E-23
	S3588	14	4	10	1	112683940	112868110	184.171	314	1.04E-19	5.33E-02	1.33E-03	6.95E-23
	S3589	14	4	10	1	112985572	113194234	208.663	310	1.04E-19	5.33E-02	1.33E-03	6.95E-23
	S3590	14	5	9	1	113271452	113492013	220.562	406	4.51E-32	6.67E-02	1.20E-03	3.02E-35
	S3591	14	4	10	1	117024431	117558213	533.783	1108	1.04E-19	5.33E-02	1.33E-03	6.95E-23
	S3604	14	3	11	2	105640761	105903778	263.018	565	1.74E-10	4.00E-02	1.47E-03	1.17E-13
	S3621	14	5	9	3	43884819	44031978	147.16	223	4.51E-32	6.67E-02	1.20E-03	3.02E-35
	S3623	14	5	9	3	142328162	142820107	491.946	605	4.51E-32	6.67E-02	1.20E-03	3.02E-35
	S3631	14	3	11	4	80439027	80572955	133.929	177	1.74E-10	4.00E-02	1.47E-03	1.17E-13
	S3633	14	5	9	4	100327069	100689879	362.811	781	4.51E-32	6.67E-02	1.20E-03	3.02E-35

	S3635	14	4	10	4	111629692	112326279	696.588	839	1.04E-19	5.33E-02	1.33E-03	6.95E-23
	S3637	14	5	9	4	152491242	152626885	135.644	121	4.51E-32	6.67E-02	1.20E-03	3.02E-35
	S3640	14	3	11	4	161328894	162324602	995.709	2003	1.74E-10	4.00E-02	1.47E-03	1.17E-13
	S3642	14	4	10	5	55224050	55413868	189.819	243	1.04E-19	5.33E-02	1.33E-03	6.95E-23
	S3643	14	4	10	5	55593749	56123279	529.531	779	1.04E-19	5.33E-02	1.33E-03	6.95E-23
	S3646	14	5	9	5	65783584	66215943	432.36	568	4.51E-32	6.67E-02	1.20E-03	3.02E-35
	S3647	14	5	9	5	97176979	97453455	276.477	473	4.51E-32	6.67E-02	1.20E-03	3.02E-35
	S3651	14	3	11	5	160974709	161401577	426.869	908	1.74E-10	4.00E-02	1.47E-03	1.17E-13
	S3652	14	3	11	5	161703565	161870295	166.731	347	1.74E-10	4.00E-02	1.47E-03	1.17E-13
	S3661	14	3	11	6	131646664	131801369	154.706	177	1.74E-10	4.00E-02	1.47E-03	1.17E-13
	S3663	14	5	9	6	162515945	163183260	667.316	1299	4.51E-32	6.67E-02	1.20E-03	3.02E-35
	S3668	14	5	9	7	109100534	109589327	488.794	873	4.51E-32	6.67E-02	1.20E-03	3.02E-35
	S3674	14	3	11	8	95577650	95684951	107.302	259	1.74E-10	4.00E-02	1.47E-03	1.17E-13
	S3676	14	3	11	8	96300258	96435196	134.939	233	1.74E-10	4.00E-02	1.47E-03	1.17E-13

	S3679	14	5	9	9	104299284	104783622	484.339	850	4.51E-32	6.67E-02	1.20E-03	3.02E-35
	S3682	14	4	10	9	110007352	110219729	212.378	462	1.04E-19	5.33E-02	1.33E-03	6.95E-23
	S3683	14	4	10	9	112215418	112505048	289.631	508	1.04E-19	5.33E-02	1.33E-03	6.95E-23
	S3698	14	3	11	12	29224621	29496222	271.602	524	1.74E-10	4.00E-02	1.47E-03	1.17E-13
	S3700	14	3	11	12	46848016	47082605	234.59	425	1.74E-10	4.00E-02	1.47E-03	1.17E-13
	S3704	14	3	11	12	102210457	102502099	291.643	321	1.74E-10	4.00E-02	1.47E-03	1.17E-13
	S3708	14	3	11	13	76566952	76809889	242.938	452	1.74E-10	4.00E-02	1.47E-03	1.17E-13
	S3709	14	3	11	13	92656308	93204655	548.348	916	1.74E-10	4.00E-02	1.47E-03	1.17E-13
	S3719	14	4	10	15	46794051	47655539	861.489	1360	1.04E-19	5.33E-02	1.33E-03	6.95E-23
	S3723	14	6	8	16	61062499	61737667	675.169	828	1.39E-47	8.00E-02	1.07E-03	9.32E-51
	S3736	13	4	9	1	158051970	158405408	353.439	790	3.22E-21	5.33E-02	1.20E-03	2.16E-24
	S3737	13	4	9	1	158410638	158668470	257.833	575	3.22E-21	5.33E-02	1.20E-03	2.16E-24
	S3738	13	3	10	1	158790636	158936329	145.694	359	2.92E-11	4.00E-02	1.33E-03	1.96E-14
	S3739	13	3	10	1	176025894	176966604	940.711	1736	2.92E-11	4.00E-02	1.33E-03	1.96E-14

	S3751	13	4	9	2	49830251	50273223	442.973	964	3.22E-21	5.33E-02	1.20E-03	2.16E-24
	S3755	13	5	8	2	77994546	78329816	335.271	635	1.49E-34	6.67E-02	1.07E-03	9.99E-38
	S3756	13	5	8	2	78578705	78792492	213.788	355	1.49E-34	6.67E-02	1.07E-03	9.99E-38
	S3770	13	5	8	3	43247708	43806958	559.251	705	1.49E-34	6.67E-02	1.07E-03	9.99E-38
	S3773	13	4	9	4	15021890	15336242	314.353	556	3.22E-21	5.33E-02	1.20E-03	2.16E-24
	S3776	13	3	10	4	77249630	77372145	122.516	304	2.92E-11	4.00E-02	1.33E-03	1.96E-14
	S3781	13	3	10	4	125345151	125533756	188.606	339	2.92E-11	4.00E-02	1.33E-03	1.96E-14
	S3784	13	4	9	4	154900767	155009051	108.285	155	3.22E-21	5.33E-02	1.20E-03	2.16E-24
	S3790	13	5	8	5	56391208	56606118	214.911	466	1.49E-34	6.67E-02	1.07E-03	9.99E-38
	S3807	13	4	9	6	142816051	143009259	193.209	349	3.22E-21	5.33E-02	1.20E-03	2.16E-24
	S3808	13	4	9	7	23067749	23926540	858.792	1480	3.22E-21	5.33E-02	1.20E-03	2.16E-24
	S3826	13	4	9	9	32939659	33090322	150.664	293	3.22E-21	5.33E-02	1.20E-03	2.16E-24
	S3827	13	4	9	9	83354952	84054511	699.56	1341	3.22E-21	5.33E-02	1.20E-03	2.16E-24
	S3828	13	4	9	9	105079259	105519419	440.161	629	3.22E-21	5.33E-02	1.20E-03	2.16E-24

	S3831	13	4	9	9	109603390	109981608	378.219	868	3.22E-21	5.33E-02	1.20E-03	2.16E-24
	S3832	13	5	8	9	122808071	123055769	247.699	245	1.49E-34	6.67E-02	1.07E-03	9.99E-38
	S3841	13	3	10	10	88700053	88986603	286.551	480	2.92E-11	4.00E-02	1.33E-03	1.96E-14
	S3848	13	3	10	12	22168408	22587409	419.002	576	2.92E-11	4.00E-02	1.33E-03	1.96E-14
	S3873	13	5	8	14	50513086	50640439	127.354	178	1.49E-34	6.67E-02	1.07E-03	9.99E-38
	S3876	13	4	9	14	68661235	68850260	189.026	474	3.22E-21	5.33E-02	1.20E-03	2.16E-24
	S3877	13	3	10	14	69353697	69669707	316.011	421	2.92E-11	4.00E-02	1.33E-03	1.96E-14
	S3891	13	3	10	15	78420357	78662037	241.681	400	2.92E-11	4.00E-02	1.33E-03	1.96E-14
	S3894	13	3	10	16	64747261	65197000	449.74	962	2.92E-11	4.00E-02	1.33E-03	1.96E-14
	S3897	13	3	10	16	74092772	74280381	187.61	358	2.92E-11	4.00E-02	1.33E-03	1.96E-14
	S3899	13	4	9	16	75488802	75615181	126.38	215	3.22E-21	5.33E-02	1.20E-03	2.16E-24
	S3901	13	3	10	16	76012030	76207515	195.486	355	2.92E-11	4.00E-02	1.33E-03	1.96E-14
	S3905	13	3	10	18	49658476	50557654	899.179	1740	2.92E-11	4.00E-02	1.33E-03	1.96E-14
	S3908	13	4	9	20	44962225	46208137	1245.913	2007	3.22E-21	5.33E-02	1.20E-03	2.16E-24

	S3915	12	3	9	1	105160481	105349960	189.48	426	3.64E-12	4.00E-02	1.20E-03	2.44E-15
	S3918	12	3	9	1	169116349	169504265	387.917	826	3.64E-12	4.00E-02	1.20E-03	2.44E-15
	S3919	12	3	9	1	169552079	169679328	127.25	317	3.64E-12	4.00E-02	1.20E-03	2.44E-15
	S3926	12	3	9	1	210074471	210244031	169.561	249	3.64E-12	4.00E-02	1.20E-03	2.44E-15
	S3944	12	3	9	2	180600610	180769937	169.328	370	3.64E-12	4.00E-02	1.20E-03	2.44E-15
	S3951	12	4	8	3	42818666	43057981	239.316	321	5.64E-23	5.33E-02	1.07E-03	3.78E-26
	S3956	12	5	7	3	143529605	143642198	112.594	236	1.90E-37	6.67E-02	9.33E-04	1.28E-40
	S3960	12	4	8	4	12137081	13072922	935.842	1989	5.64E-23	5.33E-02	1.07E-03	3.78E-26
	S3964	12	4	8	4	55690554	56662840	972.287	1845	5.64E-23	5.33E-02	1.07E-03	3.78E-26
	S3977	12	5	7	5	66336449	66615465	279.017	559	1.90E-37	6.67E-02	9.33E-04	1.28E-40
	S3979	12	3	9	5	72186060	73204954	1018.895	1358	3.64E-12	4.00E-02	1.20E-03	2.44E-15
	S3980	12	3	9	5	74945323	76297886	1352.564	2231	3.64E-12	4.00E-02	1.20E-03	2.44E-15
	S3981	12	5	7	5	96626713	96745995	119.283	236	1.90E-37	6.67E-02	9.33E-04	1.28E-40
	S3990	12	3	9	6	132608199	133163967	555.769	978	3.64E-12	4.00E-02	1.20E-03	2.44E-15

	S3991	12	4	8	7	24108825	24212310	103.486	213	5.64E-23	5.33E-02	1.07E-03	3.78E-26
	S4002	12	3	9	7	80201263	80436361	235.099	398	3.64E-12	4.00E-02	1.20E-03	2.44E-15
	S4003	12	3	9	7	80531052	80701284	170.233	242	3.64E-12	4.00E-02	1.20E-03	2.44E-15
	S4012	12	3	9	7	94921248	95451030	529.783	728	3.64E-12	4.00E-02	1.20E-03	2.44E-15
	S4018	12	3	9	8	95194325	95374265	179.941	374	3.64E-12	4.00E-02	1.20E-03	2.44E-15
	S4026	12	3	9	9	84627121	84802872	175.752	283	3.64E-12	4.00E-02	1.20E-03	2.44E-15
	S4027	12	3	9	9	85095932	85201941	106.01	151	3.64E-12	4.00E-02	1.20E-03	2.44E-15
	S4031	12	3	9	9	122024841	122181435	156.595	224	3.64E-12	4.00E-02	1.20E-03	2.44E-15
	S4032	12	3	9	9	122225314	122376416	151.103	157	3.64E-12	4.00E-02	1.20E-03	2.44E-15
	S4034	12	4	8	9	123984155	124102218	118.064	250	5.64E-23	5.33E-02	1.07E-03	3.78E-26
	S4038	12	3	9	10	89225030	89505741	280.712	458	3.64E-12	4.00E-02	1.20E-03	2.44E-15
	S4039	12	3	9	10	89521788	89865786	343.999	686	3.64E-12	4.00E-02	1.20E-03	2.44E-15
	S4043	12	7	5	10	97987740	98253487	265.748	335	1.97E-77	9.33E-02	6.66E-04	1.32E-80
	S4049	12	3	9	11	101006738	101288537	281.8	601	3.64E-12	4.00E-02	1.20E-03	2.44E-15

	S4051	12	4	8	11	102163463	102359398	195.936	243	5.64E-23	5.33E-02	1.07E-03	3.78E-26
	S4053	12	4	8	11	103042416	103185625	143.21	221	5.64E-23	5.33E-02	1.07E-03	3.78E-26
	S4056	12	3	9	12	21026265	21527675	501.411	1106	3.64E-12	4.00E-02	1.20E-03	2.44E-15
	S4057	12	3	9	12	21594374	21963053	368.68	364	3.64E-12	4.00E-02	1.20E-03	2.44E-15
	S4066	12	3	9	12	105549022	105811720	262.699	511	3.64E-12	4.00E-02	1.20E-03	2.44E-15
	S4071	12	4	8	14	51245994	51597625	351.632	780	5.64E-23	5.33E-02	1.07E-03	3.78E-26
	S4079	12	4	8	15	55357863	55537491	179.629	378	5.64E-23	5.33E-02	1.07E-03	3.78E-26
	S4082	12	3	9	15	79283727	79560175	276.449	574	3.64E-12	4.00E-02	1.20E-03	2.44E-15
	S4083	12	4	8	15	80221491	80334134	112.644	262	5.64E-23	5.33E-02	1.07E-03	3.78E-26
	S4084	12	6	6	16	50982680	51286761	304.082	587	1.35E-55	8.00E-02	7.99E-04	9.04E-59
	S4086	12	3	9	16	76398926	76558842	159.917	368	3.64E-12	4.00E-02	1.20E-03	2.44E-15
	S4089	12	3	9	18	35402493	36084761	682.269	1161	3.64E-12	4.00E-02	1.20E-03	2.44E-15
	S4097	12	3	9	20	48588786	48882173	293.388	571	3.64E-12	4.00E-02	1.20E-03	2.44E-15
	S4100	11	5	6	1	7698700	9019095	1320.396	2489	7.27E-41	6.67E-02	7.99E-04	4.87E-44

	S4103	11	2	9	1	36142826	36392544	249.719	285	2.23E-05	2.67E-02	1.20E-03	1.49E-08
	S4107	11	2	9	1	84756600	84925457	168.858	438	2.23E-05	2.67E-02	1.20E-03	1.49E-08
	S4108	11	4	7	1	107493034	107984430	491.397	1164	4.75E-25	5.33E-02	9.33E-04	3.19E-28
	S4111	11	3	8	1	115493253	115870126	376.874	780	3.11E-13	4.00E-02	1.07E-03	2.09E-16
	S4113	11	3	8	1	159146030	159758862	612.833	1450	3.11E-13	4.00E-02	1.07E-03	2.09E-16
	S4115	11	3	8	1	160672214	160905887	233.674	426	3.11E-13	4.00E-02	1.07E-03	2.09E-16
	S4116	11	3	8	1	160950381	161259053	308.673	581	3.11E-13	4.00E-02	1.07E-03	2.09E-16
	S4122	11	2	9	1	184020326	184627672	607.347	1163	2.23E-05	2.67E-02	1.20E-03	1.49E-08
	S4138	11	3	8	2	105262911	105434566	171.656	376	3.11E-13	4.00E-02	1.07E-03	2.09E-16
	S4150	11	2	9	2	228215401	228465495	250.095	483	2.23E-05	2.67E-02	1.20E-03	1.49E-08
	S4153	11	2	9	3	16764179	17118586	354.408	641	2.23E-05	2.67E-02	1.20E-03	1.49E-08
	S4154	11	2	9	3	17148999	17960084	811.086	857	2.23E-05	2.67E-02	1.20E-03	1.49E-08
	S4157	11	4	7	3	42513672	42670027	156.356	240	4.75E-25	5.33E-02	9.33E-04	3.19E-28
	S4158	11	3	8	3	64560783	64893582	332.8	627	3.11E-13	4.00E-02	1.07E-03	2.09E-16

	S4159	11	3	8	3	64900811	65746810	846	1902	3.11E-13	4.00E-02	1.07E-03	2.09E-16
	S4160	11	3	8	3	76723424	76954294	230.871	487	3.11E-13	4.00E-02	1.07E-03	2.09E-16
	S4162	11	4	7	3	131972365	132109860	137.496	317	4.75E-25	5.33E-02	9.33E-04	3.19E-28
	S4169	11	4	7	4	13295980	13526740	230.761	236	4.75E-25	5.33E-02	9.33E-04	3.19E-28
	S4171	11	2	9	4	22600521	22829316	228.796	337	2.23E-05	2.67E-02	1.20E-03	1.49E-08
	S4172	11	2	9	4	22877503	23357754	480.252	580	2.23E-05	2.67E-02	1.20E-03	1.49E-08
	S4173	11	2	9	4	23364823	23565257	200.435	397	2.23E-05	2.67E-02	1.20E-03	1.49E-08
	S4181	11	2	9	4	153673034	153896765	223.732	388	2.23E-05	2.67E-02	1.20E-03	1.49E-08
	S4190	11	2	9	5	32866393	33474832	608.44	1190	2.23E-05	2.67E-02	1.20E-03	1.49E-08
	S4196	11	5	6	5	66974494	67142346	167.853	211	7.27E-41	6.67E-02	7.99E-04	4.87E-44
	S4197	11	5	6	5	67187297	67339845	152.549	238	7.27E-41	6.67E-02	7.99E-04	4.87E-44
	S4201	11	5	6	5	68012384	68371535	359.152	647	7.27E-41	6.67E-02	7.99E-04	4.87E-44
	S4204	11	5	6	5	126163276	126450912	287.637	615	7.27E-41	6.67E-02	7.99E-04	4.87E-44
	S4207	11	3	8	6	135386348	136047840	661.493	812	3.11E-13	4.00E-02	1.07E-03	2.09E-16

	S4208	11	4	7	6	147016387	147497968	481.582	474	4.75E-25	5.33E-02	9.33E-04	3.19E-28
	S4210	11	4	7	6	157997246	158853069	855.824	1826	4.75E-25	5.33E-02	9.33E-04	3.19E-28
	S4211	11	3	8	6	161444200	161686497	242.298	542	3.11E-13	4.00E-02	1.07E-03	2.09E-16
	S4212	11	6	5	6	163582685	163903689	321.005	630	1.08E-60	8.00E-02	6.66E-04	7.23E-64
	S4213	11	2	9	7	5297549	5842100	544.552	1163	2.23E-05	2.67E-02	1.20E-03	1.49E-08
	S4214	11	2	9	7	5864236	6838803	974.568	1654	2.23E-05	2.67E-02	1.20E-03	1.49E-08
	S4215	11	3	8	7	20089076	20225757	136.682	267	3.11E-13	4.00E-02	1.07E-03	2.09E-16
	S4228	11	2	9	7	79287574	79815754	528.181	811	2.23E-05	2.67E-02	1.20E-03	1.49E-08
	S4229	11	3	8	7	81340584	81772828	432.245	510	3.11E-13	4.00E-02	1.07E-03	2.09E-16
	S4230	11	4	7	7	81774653	82384018	609.366	1198	4.75E-25	5.33E-02	9.33E-04	3.19E-28
	S4232	11	4	7	7	94426694	94684311	257.618	360	4.75E-25	5.33E-02	9.33E-04	3.19E-28
	S4233	11	3	8	7	105403992	105509980	105.989	170	3.11E-13	4.00E-02	1.07E-03	2.09E-16
	S4234	11	4	7	7	108000008	108139685	139.678	164	4.75E-25	5.33E-02	9.33E-04	3.19E-28
	S4235	11	2	9	7	129246980	130071005	824.026	1441	2.23E-05	2.67E-02	1.20E-03	1.49E-08

	S4244	11	3	8	8	71833712	71955377	121.666	288	3.11E-13	4.00E-02	1.07E-03	2.09E-16
	S4252	11	3	8	8	129064176	129340981	276.806	463	3.11E-13	4.00E-02	1.07E-03	2.09E-16
	S4254	11	2	9	8	136664978	137613088	948.111	1700	2.23E-05	2.67E-02	1.20E-03	1.49E-08
	S4262	11	2	9	9	85399570	85977842	578.273	742	2.23E-05	2.67E-02	1.20E-03	1.49E-08
	S4264	11	5	6	9	107582729	107726008	143.28	316	7.27E-41	6.67E-02	7.99E-04	4.87E-44
	S4265	11	2	9	9	116335288	116722340	387.053	738	2.23E-05	2.67E-02	1.20E-03	1.49E-08
	S4273	11	2	9	10	82737604	83312126	574.523	1181	2.23E-05	2.67E-02	1.20E-03	1.49E-08
	S4277	11	3	8	11	22857299	23056677	199.379	462	3.11E-13	4.00E-02	1.07E-03	2.09E-16
	S4278	11	3	8	11	24064194	24418784	354.591	770	3.11E-13	4.00E-02	1.07E-03	2.09E-16
	S4279	11	3	8	11	24421480	24735779	314.3	721	3.11E-13	4.00E-02	1.07E-03	2.09E-16
	S4285	11	3	8	11	100884590	101002222	117.633	322	3.11E-13	4.00E-02	1.07E-03	2.09E-16
	S4289	11	2	9	12	69603502	70418763	815.262	1946	2.23E-05	2.67E-02	1.20E-03	1.49E-08
	S4295	11	2	9	12	108933605	109202226	268.622	416	2.23E-05	2.67E-02	1.20E-03	1.49E-08
	S4296	11	2	9	13	47410436	47555398	144.963	255	2.23E-05	2.67E-02	1.20E-03	1.49E-08

	S4299	11	3	8	13	75974766	76279883	305.118	759	3.11E-13	4.00E-02	1.07E-03	2.09E-16
	S4304	11	3	8	13	94442131	95046259	604.129	1140	3.11E-13	4.00E-02	1.07E-03	2.09E-16
	S4306	11	4	7	13	102103344	102299561	196.218	410	4.75E-25	5.33E-02	9.33E-04	3.19E-28
	S4311	11	2	9	14	56317271	56437219	119.949	288	2.23E-05	2.67E-02	1.20E-03	1.49E-08
	S4320	11	4	7	15	52973737	53244988	271.252	561	4.75E-25	5.33E-02	9.33E-04	3.19E-28
	S4323	11	5	6	15	65502862	66126696	623.835	933	7.27E-41	6.67E-02	7.99E-04	4.87E-44
	S4324	11	5	6	16	51998601	52452407	453.807	820	7.27E-41	6.67E-02	7.99E-04	4.87E-44
	S4325	11	5	6	16	58288385	58461459	173.075	271	7.27E-41	6.67E-02	7.99E-04	4.87E-44
	S4329	11	2	9	16	73544935	73865221	320.287	584	2.23E-05	2.67E-02	1.20E-03	1.49E-08
	S4330	11	3	8	16	77098072	77389530	291.459	885	3.11E-13	4.00E-02	1.07E-03	2.09E-16
	S4331	11	3	8	16	77457800	77709032	251.233	769	3.11E-13	4.00E-02	1.07E-03	2.09E-16
	S4333	11	3	8	18	27519587	27668626	149.04	262	3.11E-13	4.00E-02	1.07E-03	2.09E-16
	S4335	11	2	9	18	57290500	57670960	380.461	725	2.23E-05	2.67E-02	1.20E-03	1.49E-08
	S4340	11	2	9	20	49781513	50058789	277.277	544	2.23E-05	2.67E-02	1.20E-03	1.49E-08

	S4344	11	3	8	22	34151045	34597404	446.36	994	3.11E-13	4.00E-02	1.07E-03	2.09E-16
	S4345	11	3	8	22	34749533	34895284	145.752	318	3.11E-13	4.00E-02	1.07E-03	2.09E-16
	S4349	10	2	8	1	23015700	23814521	798.822	1199	7.49E-06	2.67E-02	1.07E-03	5.02E-09
	S4356	10	4	6	1	114799108	115297896	498.789	1189	1.54E-27	5.33E-02	7.99E-04	1.04E-30
	S4359	10	4	6	1	157345386	157481515	136.13	314	1.54E-27	5.33E-02	7.99E-04	1.04E-30
	S4360	10	3	7	1	159957347	160496566	539.22	945	1.64E-14	4.00E-02	9.33E-04	1.10E-17
	S4361	10	3	7	1	162470743	162600585	129.843	222	1.64E-14	4.00E-02	9.33E-04	1.10E-17
	S4362	10	3	7	1	162998128	163530994	532.867	1437	1.64E-14	4.00E-02	9.33E-04	1.10E-17
	S4363	10	3	7	1	163539720	164268365	728.646	1443	1.64E-14	4.00E-02	9.33E-04	1.10E-17
	S4364	10	3	7	1	168333311	168455826	122.516	237	1.64E-14	4.00E-02	9.33E-04	1.10E-17
	S4371	10	4	6	2	79508574	79696007	187.434	345	1.54E-27	5.33E-02	7.99E-04	1.04E-30
	S4386	10	2	8	3	1409603	1842964	433.362	1346	7.49E-06	2.67E-02	1.07E-03	5.02E-09
	S4387	10	3	7	3	2393584	2709509	315.926	730	1.64E-14	4.00E-02	9.33E-04	1.10E-17
	S4389	10	4	6	3	60800620	61950254	1149.635	2275	1.54E-27	5.33E-02	7.99E-04	1.04E-30

	S4391	10	3	7	3	69855721	70036135	180.415	175	1.64E-14	4.00E-02	9.33E-04	1.10E-17
	S4394	10	3	7	3	112764530	113086154	321.625	542	1.64E-14	4.00E-02	9.33E-04	1.10E-17
	S4395	10	3	7	3	123430486	123695355	264.87	309	1.64E-14	4.00E-02	9.33E-04	1.10E-17
	S4396	10	3	7	3	123876585	124550093	673.509	958	1.64E-14	4.00E-02	9.33E-04	1.10E-17
	S4397	10	3	7	3	124556912	124967889	410.978	915	1.64E-14	4.00E-02	9.33E-04	1.10E-17
	S4399	10	3	7	3	150713302	150872381	159.08	177	1.64E-14	4.00E-02	9.33E-04	1.10E-17
	S4401	10	3	7	4	13835317	14009972	174.656	350	1.64E-14	4.00E-02	9.33E-04	1.10E-17
	S4412	10	4	6	5	122198652	122478049	279.398	285	1.54E-27	5.33E-02	7.99E-04	1.04E-30
	S4413	10	5	5	5	124279867	124566139	286.273	510	5.77E-45	6.67E-02	6.66E-04	3.87E-48
	S4414	10	4	6	5	125630120	125804876	174.757	396	1.54E-27	5.33E-02	7.99E-04	1.04E-30
	S4417	10	3	7	5	134230104	135130379	900.276	1001	1.64E-14	4.00E-02	9.33E-04	1.10E-17
	S4420	10	4	6	6	39932543	40144923	212.381	376	1.54E-27	5.33E-02	7.99E-04	1.04E-30
	S4426	10	2	8	6	99841307	100029426	188.12	309	7.49E-06	2.67E-02	1.07E-03	5.02E-09
	S4429	10	4	6	6	158887949	159197271	309.323	481	1.54E-27	5.33E-02	7.99E-04	1.04E-30

	S4431	10	3	7	7	1224511	2672318	1447.808	2986	1.64E-14	4.00E-02	9.33E-04	1.10E-17
	S4432	10	3	7	7	10356343	10592588	236.246	644	1.64E-14	4.00E-02	9.33E-04	1.10E-17
	S4433	10	2	8	7	21078350	21675136	596.787	1551	7.49E-06	2.67E-02	1.07E-03	5.02E-09
	S4434	10	3	7	7	22240125	22384820	144.696	284	1.64E-14	4.00E-02	9.33E-04	1.10E-17
	S4442	10	3	7	7	128941276	129198928	257.653	414	1.64E-14	4.00E-02	9.33E-04	1.10E-17
	S4456	10	2	8	8	122658757	122769894	111.138	158	7.49E-06	2.67E-02	1.07E-03	5.02E-09
	S4457	10	3	7	8	127792869	128160234	367.366	697	1.64E-14	4.00E-02	9.33E-04	1.10E-17
	S4459	10	2	8	8	135948293	136260338	312.046	596	7.49E-06	2.67E-02	1.07E-03	5.02E-09
	S4461	10	2	8	9	10963411	11083979	120.569	352	7.49E-06	2.67E-02	1.07E-03	5.02E-09
	S4465	10	5	5	9	74714451	74845691	131.241	189	5.77E-45	6.67E-02	6.66E-04	3.87E-48
	S4467	10	2	8	9	86521274	86833583	312.31	492	7.49E-06	2.67E-02	1.07E-03	5.02E-09
	S4469	10	3	7	9	98217063	98402965	185.903	417	1.64E-14	4.00E-02	9.33E-04	1.10E-17
	S4479	10	2	8	10	119296873	119821778	524.906	965	7.49E-06	2.67E-02	1.07E-03	5.02E-09
	S4480	10	2	8	11	4427419	5075101	647.683	1628	7.49E-06	2.67E-02	1.07E-03	5.02E-09

	S4484	10	3	7	11	22312503	22625424	312.922	576	1.64E-14	4.00E-02	9.33E-04	1.10E-17
	S4485	10	2	8	11	23395219	23499332	104.114	282	7.49E-06	2.67E-02	1.07E-03	5.02E-09
	S4489	10	2	8	11	92580342	92872820	292.479	405	7.49E-06	2.67E-02	1.07E-03	5.02E-09
	S4490	10	2	8	11	94744147	94890796	146.65	108	7.49E-06	2.67E-02	1.07E-03	5.02E-09
	S4494	10	3	7	12	23086330	23494197	407.868	504	1.64E-14	4.00E-02	9.33E-04	1.10E-17
	S4495	10	3	7	12	23826293	24187936	361.644	723	1.64E-14	4.00E-02	9.33E-04	1.10E-17
	S4496	10	3	7	12	24203498	24393854	190.357	399	1.64E-14	4.00E-02	9.33E-04	1.10E-17
	S4499	10	2	8	12	64210920	64467462	256.543	353	7.49E-06	2.67E-02	1.07E-03	5.02E-09
	S4506	10	2	8	12	96432833	96763449	330.617	699	7.49E-06	2.67E-02	1.07E-03	5.02E-09
	S4513	10	4	6	12	114833879	115016132	182.254	360	1.54E-27	5.33E-02	7.99E-04	1.04E-30
	S4514	10	2	8	12	125196264	125345986	149.723	276	7.49E-06	2.67E-02	1.07E-03	5.02E-09
	S4516	10	4	6	13	90667118	90802500	135.383	252	1.54E-27	5.33E-02	7.99E-04	1.04E-30
	S4519	10	3	7	13	101094089	101653039	558.951	1137	1.64E-14	4.00E-02	9.33E-04	1.10E-17
	S4520	10	2	8	14	37036574	37593915	557.342	589	7.49E-06	2.67E-02	1.07E-03	5.02E-09

	S4521	10	3	7	14	69909652	70187041	277.39	686	1.64E-14	4.00E-02	9.33E-04	1.10E-17
	S4528	10	5	5	15	66746574	66932154	185.581	352	5.77E-45	6.67E-02	6.66E-04	3.87E-48
	S4529	10	5	5	15	66932872	67118464	185.593	385	5.77E-45	6.67E-02	6.66E-04	3.87E-48
	S4535	10	3	7	16	24668787	25063831	395.045	671	1.64E-14	4.00E-02	9.33E-04	1.10E-17
	S4537	10	5	5	16	26937027	27162303	225.277	568	5.77E-45	6.67E-02	6.66E-04	3.87E-48
	S4538	10	4	6	16	53134146	53342939	208.794	264	1.54E-27	5.33E-02	7.99E-04	1.04E-30
	S4539	10	4	6	16	56338927	56929410	590.484	1140	1.54E-27	5.33E-02	7.99E-04	1.04E-30
	S4540	10	5	5	16	57412095	57855908	443.814	847	5.77E-45	6.67E-02	6.66E-04	3.87E-48
	S4549	10	2	8	18	51453244	51588653	135.41	199	7.49E-06	2.67E-02	1.07E-03	5.02E-09
	S4552	10	4	6	19	31082574	31630862	548.289	778	1.54E-27	5.33E-02	7.99E-04	1.04E-30
	S4553	10	5	5	19	31928119	32439163	511.045	854	5.77E-45	6.67E-02	6.66E-04	3.87E-48
	S4557	10	2	8	19	39128280	39241322	113.043	182	7.49E-06	2.67E-02	1.07E-03	5.02E-09
	S4562	9	5	4	1	18808720	19714095	905.376	2184	5.66E-50	6.67E-02	5.33E-04	3.79E-53
	S4563	9	3	6	1	21530637	22019906	489.27	1353	4.50E-16	4.00E-02	7.99E-04	3.02E-19

	S4566	9	2	7	1	36598595	36701114	102.52	159	1.99E-06	2.67E-02	9.33E-04	1.33E-09
	S4567	9	2	7	1	43659475	43877501	218.027	356	1.99E-06	2.67E-02	9.33E-04	1.33E-09
	S4568	9	2	7	1	57144256	57659636	515.381	1034	1.99E-06	2.67E-02	9.33E-04	1.33E-09
	S4569	9	5	4	1	66482157	67301843	819.687	1798	5.66E-50	6.67E-02	5.33E-04	3.79E-53
	S4571	9	4	5	1	110486865	110661096	174.232	365	1.41E-30	5.33E-02	6.66E-04	9.47E-34
	S4572	9	3	6	1	167228524	167749038	520.515	1088	4.50E-16	4.00E-02	7.99E-04	3.02E-19
	S4574	9	3	6	1	199645702	200047874	402.173	907	4.50E-16	4.00E-02	7.99E-04	3.02E-19
	S4580	9	2	7	2	44183401	44309494	126.094	201	1.99E-06	2.67E-02	9.33E-04	1.33E-09
	S4592	9	3	6	3	14667411	15272505	605.095	977	4.50E-16	4.00E-02	7.99E-04	3.02E-19
	S4593	9	3	6	3	15290398	16112722	822.325	1437	4.50E-16	4.00E-02	7.99E-04	3.02E-19
	S4596	9	2	7	3	24314143	24504776	190.634	293	1.99E-06	2.67E-02	9.33E-04	1.33E-09
	S4602	9	3	6	3	70398296	71000213	601.918	830	4.50E-16	4.00E-02	7.99E-04	3.02E-19
	S4603	9	3	6	3	104575557	104676465	100.909	183	4.50E-16	4.00E-02	7.99E-04	3.02E-19
	S4605	9	2	7	3	104845312	105113360	268.049	632	1.99E-06	2.67E-02	9.33E-04	1.33E-09

	S4606	9	2	7	3	105856566	106105366	248.801	388	1.99E-06	2.67E-02	9.33E-04	1.33E-09
	S4607	9	2	7	3	106200948	106406190	205.243	335	1.99E-06	2.67E-02	9.33E-04	1.33E-09
	S4610	9	3	6	3	113967463	114235308	267.846	377	4.50E-16	4.00E-02	7.99E-04	3.02E-19
	S4611	9	3	6	3	114747253	114850556	103.304	121	4.50E-16	4.00E-02	7.99E-04	3.02E-19
	S4612	9	3	6	3	114927416	115210665	283.25	232	4.50E-16	4.00E-02	7.99E-04	3.02E-19
	S4614	9	3	6	3	126167480	126377383	209.904	466	4.50E-16	4.00E-02	7.99E-04	3.02E-19
	S4615	9	3	6	3	149949639	150244227	294.589	539	4.50E-16	4.00E-02	7.99E-04	3.02E-19
	S4620	9	2	7	4	20897300	21145173	247.874	534	1.99E-06	2.67E-02	9.33E-04	1.33E-09
	S4622	9	3	6	4	54850038	55016727	166.69	278	4.50E-16	4.00E-02	7.99E-04	3.02E-19
	S4624	9	2	7	4	163334277	163496093	161.817	304	1.99E-06	2.67E-02	9.33E-04	1.33E-09
	S4628	9	2	7	5	76921710	77378485	456.776	833	1.99E-06	2.67E-02	9.33E-04	1.33E-09
	S4630	9	3	6	5	79805294	80260350	455.057	940	4.50E-16	4.00E-02	7.99E-04	3.02E-19
	S4632	9	4	5	5	124578128	125507383	929.256	1900	1.41E-30	5.33E-02	6.66E-04	9.47E-34
	S4634	9	2	7	5	159708201	159855895	147.695	227	1.99E-06	2.67E-02	9.33E-04	1.33E-09

	S4635	9	3	6	5	159869312	160150806	281.495	376	4.50E-16	4.00E-02	7.99E-04	3.02E-19
	S4636	9	3	6	5	163697822	164049107	351.286	728	4.50E-16	4.00E-02	7.99E-04	3.02E-19
	S4638	9	2	7	6	21818985	22058588	239.604	480	1.99E-06	2.67E-02	9.33E-04	1.33E-09
	S4639	9	2	7	6	22960022	23228668	268.647	486	1.99E-06	2.67E-02	9.33E-04	1.33E-09
	S4642	9	3	6	6	40904167	41338238	434.072	655	4.50E-16	4.00E-02	7.99E-04	3.02E-19
	S4645	9	4	5	6	147718119	147995687	277.569	642	1.41E-30	5.33E-02	6.66E-04	9.47E-34
	S4647	9	3	6	6	151376381	151902397	526.017	960	4.50E-16	4.00E-02	7.99E-04	3.02E-19
	S4648	9	5	4	6	164602133	165511640	909.508	2158	5.66E-50	6.67E-02	5.33E-04	3.79E-53
	S4649	9	3	6	7	4637402	4831264	193.863	299	4.50E-16	4.00E-02	7.99E-04	3.02E-19
	S4650	9	3	6	7	4847612	5101392	253.781	665	4.50E-16	4.00E-02	7.99E-04	3.02E-19
	S4651	9	4	5	7	14149680	14346907	197.228	412	1.41E-30	5.33E-02	6.66E-04	9.47E-34
	S4652	9	2	7	7	19611482	20058372	446.891	994	1.99E-06	2.67E-02	9.33E-04	1.33E-09
	S4654	9	2	7	7	32418271	33234842	816.572	1790	1.99E-06	2.67E-02	9.33E-04	1.33E-09
	S4655	9	2	7	7	33332922	33507007	174.086	350	1.99E-06	2.67E-02	9.33E-04	1.33E-09

	S4661	9	4	5	7	128381802	128733088	351.287	828	1.41E-30	5.33E-02	6.66E-04	9.47E-34
	S4672	9	2	7	8	127389769	127519182	129.414	314	1.99E-06	2.67E-02	9.33E-04	1.33E-09
	S4674	9	2	7	8	127557457	127788701	231.245	431	1.99E-06	2.67E-02	9.33E-04	1.33E-09
	S4679	9	4	5	9	75254932	75435447	180.516	385	1.41E-30	5.33E-02	6.66E-04	9.47E-34
	S4682	9	2	7	10	8740581	9163012	422.432	804	1.99E-06	2.67E-02	9.33E-04	1.33E-09
	S4683	9	2	7	10	9646289	9907872	261.584	459	1.99E-06	2.67E-02	9.33E-04	1.33E-09
	S4685	9	2	7	10	59823437	60007089	183.653	328	1.99E-06	2.67E-02	9.33E-04	1.33E-09
	S4686	9	4	5	10	97140410	97577159	436.75	704	1.41E-30	5.33E-02	6.66E-04	9.47E-34
	S4687	9	4	5	10	97608158	97730376	122.219	182	1.41E-30	5.33E-02	6.66E-04	9.47E-34
	S4688	9	5	4	10	97732914	97873937	141.024	167	5.66E-50	6.67E-02	5.33E-04	3.79E-53
	S4689	9	2	7	10	121705316	122133359	428.044	1000	1.99E-06	2.67E-02	9.33E-04	1.33E-09
	S4694	9	2	7	11	79503121	79696782	193.662	359	1.99E-06	2.67E-02	9.33E-04	1.33E-09
	S4696	9	3	6	11	99742967	99844216	101.25	286	4.50E-16	4.00E-02	7.99E-04	3.02E-19
	S4699	9	3	6	12	16365732	16583317	217.586	300	4.50E-16	4.00E-02	7.99E-04	3.02E-19

	S4709	9	4	5	12	114394173	114547018	152.846	289	1.41E-30	5.33E-02	6.66E-04	9.47E-34
	S4710	9	4	5	12	115867416	116351437	484.022	688	1.41E-30	5.33E-02	6.66E-04	9.47E-34
	S4711	9	4	5	12	116365752	116486938	121.187	266	1.41E-30	5.33E-02	6.66E-04	9.47E-34
	S4717	9	2	7	13	44669217	44779412	110.196	152	1.99E-06	2.67E-02	9.33E-04	1.33E-09
	S4725	9	3	6	14	91226541	91747623	521.083	977	4.50E-16	4.00E-02	7.99E-04	3.02E-19
	S4727	9	3	6	14	94809152	95311752	502.601	1121	4.50E-16	4.00E-02	7.99E-04	3.02E-19
	S4730	9	5	4	16	16854795	17110288	255.494	454	5.66E-50	6.67E-02	5.33E-04	3.79E-53
	S4731	9	3	6	16	19279092	19384618	105.527	226	4.50E-16	4.00E-02	7.99E-04	3.02E-19
	S4732	9	5	4	16	27223625	27840555	616.931	818	5.66E-50	6.67E-02	5.33E-04	3.79E-53
	S4733	9	4	5	16	52757088	52945399	188.312	335	1.41E-30	5.33E-02	6.66E-04	9.47E-34
	S4734	9	3	6	16	55188902	55673342	484.441	858	4.50E-16	4.00E-02	7.99E-04	3.02E-19
	S4736	9	2	7	16	78094370	78606465	512.096	1753	1.99E-06	2.67E-02	9.33E-04	1.33E-09
	S4738	9	3	6	18	26520059	26782785	262.727	518	4.50E-16	4.00E-02	7.99E-04	3.02E-19
	S4746	9	3	6	20	18485702	18914007	428.306	920	4.50E-16	4.00E-02	7.99E-04	3.02E-19

	S4749	9	3	6	20	24078358	24280743	202.386	345	4.50E-16	4.00E-02	7.99E-04	3.02E-19
	S4750	9	3	6	21	33354925	33852066	497.142	667	4.50E-16	4.00E-02	7.99E-04	3.02E-19
	S4751	9	3	6	22	33213881	33356446	142.566	228	4.50E-16	4.00E-02	7.99E-04	3.02E-19
	S4753	9	2	7	22	47270455	47878603	608.149	1339	1.99E-06	2.67E-02	9.33E-04	1.33E-09
	S4754	8	4	4	1	6601672	7187275	585.604	1273	2.26E-34	5.33E-02	5.33E-04	1.52E-37
	S4757	8	2	6	1	38820417	38954990	134.574	313	3.81E-07	2.67E-02	7.99E-04	2.56E-10
	S4758	8	2	6	1	38973972	39811650	837.679	1602	3.81E-07	2.67E-02	7.99E-04	2.56E-10
	S4761	8	4	4	1	109905062	110381992	476.931	786	2.26E-34	5.33E-02	5.33E-04	1.52E-37
	S4763	8	3	5	1	200181610	200447162	265.553	441	5.06E-18	4.00E-02	6.66E-04	3.40E-21
	S4765	8	2	6	1	207118477	207969549	851.073	1522	3.81E-07	2.67E-02	7.99E-04	2.56E-10
	S4769	8	2	6	2	1797292	2003713	206.422	402	3.81E-07	2.67E-02	7.99E-04	2.56E-10
	S4771	8	2	6	2	2247281	2532844	285.564	536	3.81E-07	2.67E-02	7.99E-04	2.56E-10
	S4778	8	2	6	3	30227026	30682944	455.919	925	3.81E-07	2.67E-02	7.99E-04	2.56E-10
	S4780	8	2	6	3	54541513	55315794	774.282	1544	3.81E-07	2.67E-02	7.99E-04	2.56E-10

	S4781	8	3	5	3	60424973	60591036	166.064	485	5.06E-18	4.00E-02	6.66E-04	3.40E-21
	S4782	8	3	5	3	66660387	66856351	195.965	440	5.06E-18	4.00E-02	6.66E-04	3.40E-21
	S4783	8	3	5	3	67924310	68164445	240.136	395	5.06E-18	4.00E-02	6.66E-04	3.40E-21
	S4784	8	3	5	3	68285725	69387118	1101.394	1919	5.06E-18	4.00E-02	6.66E-04	3.40E-21
	S4785	8	2	6	3	75762886	76444458	681.573	975	3.81E-07	2.67E-02	7.99E-04	2.56E-10
	S4786	8	4	4	3	175327907	175534037	206.131	406	2.26E-34	5.33E-02	5.33E-04	1.52E-37
	S4789	8	2	6	4	82172483	82695162	522.68	947	3.81E-07	2.67E-02	7.99E-04	2.56E-10
	S4797	8	2	6	5	76506020	76662097	156.078	309	3.81E-07	2.67E-02	7.99E-04	2.56E-10
	S4800	8	4	4	5	145936404	146357478	421.075	574	2.26E-34	5.33E-02	5.33E-04	1.52E-37
	S4803	8	3	5	6	6226151	6593462	367.312	748	5.06E-18	4.00E-02	6.66E-04	3.40E-21
	S4804	8	4	4	6	7006326	7232156	225.831	390	2.26E-34	5.33E-02	5.33E-04	1.52E-37
	S4805	8	4	4	6	7569093	7761495	192.403	414	2.26E-34	5.33E-02	5.33E-04	1.52E-37
	S4806	8	4	4	6	7772466	8376017	603.552	1269	2.26E-34	5.33E-02	5.33E-04	1.52E-37
	S4807	8	4	4	6	8378389	8887404	509.016	1051	2.26E-34	5.33E-02	5.33E-04	1.52E-37

	S4817	8	4	4	6	137283083	138644122	1361.04	2503	2.26E-34	5.33E-02	5.33E-04	1.52E-37
	S4827	8	2	6	8	23837494	25028994	1191.501	1710	3.81E-07	2.67E-02	7.99E-04	2.56E-10
	S4829	8	2	6	8	68212014	68314186	102.173	181	3.81E-07	2.67E-02	7.99E-04	2.56E-10
	S4830	8	2	6	8	68372232	68973448	601.217	1041	3.81E-07	2.67E-02	7.99E-04	2.56E-10
	S4831	8	2	6	8	123409792	123597973	188.182	466	3.81E-07	2.67E-02	7.99E-04	2.56E-10
	S4832	8	2	6	8	123681170	123853016	171.847	276	3.81E-07	2.67E-02	7.99E-04	2.56E-10
	S4833	8	2	6	8	123900286	125259706	1359.421	2329	3.81E-07	2.67E-02	7.99E-04	2.56E-10
	S4834	8	2	6	8	134563496	134814337	250.842	536	3.81E-07	2.67E-02	7.99E-04	2.56E-10
	S4835	8	2	6	9	4740545	4883257	142.713	317	3.81E-07	2.67E-02	7.99E-04	2.56E-10
	S4842	8	2	6	9	78349772	78998022	648.251	1141	3.81E-07	2.67E-02	7.99E-04	2.56E-10
	S4848	8	2	6	9	113648961	113817969	169.009	345	3.81E-07	2.67E-02	7.99E-04	2.56E-10
	S4851	8	2	6	9	126850851	127066219	215.369	293	3.81E-07	2.67E-02	7.99E-04	2.56E-10
	S4856	8	3	5	10	76388111	76900800	512.69	703	5.06E-18	4.00E-02	6.66E-04	3.40E-21
	S4858	8	2	6	10	116756707	116971850	215.144	227	3.81E-07	2.67E-02	7.99E-04	2.56E-10

	S4861	8	3	5	11	34628852	35482083	853.232	1728	5.06E-18	4.00E-02	6.66E-04	3.40E-21
	S4862	8	3	5	11	61463423	61865710	402.288	458	5.06E-18	4.00E-02	6.66E-04	3.40E-21
	S4865	8	2	6	11	95072383	95174814	102.432	195	3.81E-07	2.67E-02	7.99E-04	2.56E-10
	S4868	8	2	6	12	11231190	11542930	311.741	460	3.81E-07	2.67E-02	7.99E-04	2.56E-10
	S4870	8	2	6	12	24912795	25543144	630.35	1474	3.81E-07	2.67E-02	7.99E-04	2.56E-10
	S4873	8	4	4	12	116519565	116631678	112.114	225	2.26E-34	5.33E-02	5.33E-04	1.52E-37
	S4876	8	2	6	13	44887231	46175106	1287.876	1862	3.81E-07	2.67E-02	7.99E-04	2.56E-10
	S4888	8	3	5	15	23960958	25248109	1287.152	1985	5.06E-18	4.00E-02	6.66E-04	3.40E-21
	S4890	8	4	4	15	61048372	61374329	325.958	531	2.26E-34	5.33E-02	5.33E-04	1.52E-37
	S4891	8	4	4	15	61540658	61835798	295.141	568	2.26E-34	5.33E-02	5.33E-04	1.52E-37
	S4892	8	5	3	15	62261912	62400579	138.668	302	3.12E-56	6.67E-02	4.00E-04	2.09E-59
	S4895	8	5	3	15	68589185	68724294	135.11	215	3.12E-56	6.67E-02	4.00E-04	2.09E-59
	S4896	8	2	6	15	97302448	97534348	231.901	455	3.81E-07	2.67E-02	7.99E-04	2.56E-10
	S4898	8	3	5	16	12209094	12515873	306.78	617	5.06E-18	4.00E-02	6.66E-04	3.40E-21

	S4900	8	4	4	16	62922806	63074628	151.823	250	2.26E-34	5.33E-02	5.33E-04	1.52E-37
	S4901	8	2	6	16	80276142	81134969	858.828	1923	3.81E-07	2.67E-02	7.99E-04	2.56E-10
	S4907	8	3	5	18	13572355	14132941	560.587	1012	5.06E-18	4.00E-02	6.66E-04	3.40E-21
	S4909	8	2	6	18	25631759	26459190	827.432	1303	3.81E-07	2.67E-02	7.99E-04	2.56E-10
	S4911	8	2	6	18	57854558	58115947	261.39	509	3.81E-07	2.67E-02	7.99E-04	2.56E-10
	S4912	8	2	6	18	58117197	59191972	1074.776	2069	3.81E-07	2.67E-02	7.99E-04	2.56E-10
	S4913	8	3	5	18	60044465	60425987	381.523	538	5.06E-18	4.00E-02	6.66E-04	3.40E-21
	S4915	8	3	5	19	4976401	5245619	269.219	664	5.06E-18	4.00E-02	6.66E-04	3.40E-21
	S4916	8	3	5	19	32874928	32982129	107.202	302	5.06E-18	4.00E-02	6.66E-04	3.40E-21
	S4923	8	3	5	20	46952464	47087472	135.009	243	5.06E-18	4.00E-02	6.66E-04	3.40E-21
	S4925	8	2	6	20	52062666	53169564	1106.899	1851	3.81E-07	2.67E-02	7.99E-04	2.56E-10
	S4929	8	2	6	21	43836504	44092475	255.972	396	3.81E-07	2.67E-02	7.99E-04	2.56E-10
	S4934	8	2	6	22	39362248	39570681	208.434	266	3.81E-07	2.67E-02	7.99E-04	2.56E-10
	S4936	7	3	4	1	9989294	10944390	955.097	1856	1.59E-20	4.00E-02	5.33E-04	1.07E-23

	S4940	7	2	5	1	30133716	30483565	349.85	797	4.60E-08	2.67E-02	6.66E-04	3.09E-11
	S4948	7	2	5	1	238558740	238747202	188.463	384	4.60E-08	2.67E-02	6.66E-04	3.09E-11
	S4953	7	2	5	2	191627083	191733298	106.216	156	4.60E-08	2.67E-02	6.66E-04	3.09E-11
	S4958	7	2	5	3	3516970	3658272	141.303	321	4.60E-08	2.67E-02	6.66E-04	3.09E-11
	S4964	7	3	4	3	183467293	183579399	112.107	193	1.59E-20	4.00E-02	5.33E-04	1.07E-23
	S4965	7	3	4	3	187063125	187580707	517.583	999	1.59E-20	4.00E-02	5.33E-04	1.07E-23
	S4974	7	3	4	5	80420045	81166695	746.651	1526	1.59E-20	4.00E-02	5.33E-04	1.07E-23
	S4975	7	3	4	5	81307822	81629911	322.09	500	1.59E-20	4.00E-02	5.33E-04	1.07E-23
	S4978	7	2	5	5	164379548	164974265	594.718	822	4.60E-08	2.67E-02	6.66E-04	3.09E-11
	S4979	7	2	5	5	165424979	165526110	101.132	186	4.60E-08	2.67E-02	6.66E-04	3.09E-11
	S4984	7	4	3	6	8991328	9190384	199.057	319	3.00E-39	5.33E-02	4.00E-04	2.01E-42
	S4991	7	3	4	6	138972226	139095603	123.378	234	1.59E-20	4.00E-02	5.33E-04	1.07E-23
	S4992	7	2	5	6	148891314	149289626	398.313	761	4.60E-08	2.67E-02	6.66E-04	3.09E-11
	S4994	7	2	5	6	155873417	156554841	681.425	1356	4.60E-08	2.67E-02	6.66E-04	3.09E-11

	S4996	7	2	5	7	7096062	7401521	305.46	784	4.60E-08	2.67E-02	6.66E-04	3.09E-11
	S4998	7	3	4	7	8213875	8715936	502.062	977	1.59E-20	4.00E-02	5.33E-04	1.07E-23
	S5000	7	3	4	7	15103127	15582769	479.643	1019	1.59E-20	4.00E-02	5.33E-04	1.07E-23
	S5001	7	3	4	7	18110576	18352248	241.673	442	1.59E-20	4.00E-02	5.33E-04	1.07E-23
	S5004	7	3	4	7	93394948	94055956	661.009	1003	1.59E-20	4.00E-02	5.33E-04	1.07E-23
	S5007	7	2	5	7	157593810	157740587	146.778	308	4.60E-08	2.67E-02	6.66E-04	3.09E-11
	S5009	7	2	5	8	23635862	23768832	132.971	298	4.60E-08	2.67E-02	6.66E-04	3.09E-11
	S5021	7	2	5	10	77417925	77865495	447.571	826	4.60E-08	2.67E-02	6.66E-04	3.09E-11
	S5022	7	2	5	10	117437399	117731241	293.843	555	4.60E-08	2.67E-02	6.66E-04	3.09E-11
	S5029	7	3	4	11	114388318	115822692	1434.375	2373	1.59E-20	4.00E-02	5.33E-04	1.07E-23
	S5030	7	2	5	11	118955052	119125195	170.144	260	4.60E-08	2.67E-02	6.66E-04	3.09E-11
	S5031	7	4	3	12	1010556	1804326	793.771	1395	3.00E-39	5.33E-02	4.00E-04	2.01E-42
	S5033	7	3	4	12	20006480	20374978	368.499	705	1.59E-20	4.00E-02	5.33E-04	1.07E-23
	S5035	7	2	5	12	53531587	53710439	178.853	279	4.60E-08	2.67E-02	6.66E-04	3.09E-11

	S5040	7	2	5	12	118024123	118462612	438.49	711	4.60E-08	2.67E-02	6.66E-04	3.09E-11
	S5044	7	2	5	13	74723411	75013512	290.102	404	4.60E-08	2.67E-02	6.66E-04	3.09E-11
	S5052	7	4	3	15	69823443	69935918	112.476	250	3.00E-39	5.33E-02	4.00E-04	2.01E-42
	S5055	7	2	5	16	12774758	12919187	144.43	318	4.60E-08	2.67E-02	6.66E-04	3.09E-11
	S5056	7	3	4	16	13079905	13245014	165.11	342	1.59E-20	4.00E-02	5.33E-04	1.07E-23
	S5061	7	3	4	18	4375900	4683756	307.857	638	1.59E-20	4.00E-02	5.33E-04	1.07E-23
	S5062	7	3	4	18	12484728	13458064	973.337	1836	1.59E-20	4.00E-02	5.33E-04	1.07E-23
	S5064	7	2	5	18	59698392	59802901	104.51	205	4.60E-08	2.67E-02	6.66E-04	3.09E-11
	S5066	7	2	5	18	61178794	61339044	160.251	251	4.60E-08	2.67E-02	6.66E-04	3.09E-11
	S5072	7	3	4	20	47972893	48518558	545.666	959	1.59E-20	4.00E-02	5.33E-04	1.07E-23
	S5073	7	3	4	21	34342724	34746398	403.675	563	1.59E-20	4.00E-02	5.33E-04	1.07E-23
	S5081	7	3	4	22	39179687	39289038	109.352	221	1.59E-20	4.00E-02	5.33E-04	1.07E-23
	S5085	6	2	4	1	11805747	12134732	328.986	715	2.77E-09	2.67E-02	5.33E-04	1.86E-12
	S5086	6	2	4	1	12240622	13373715	1133.094	1014	2.77E-09	2.67E-02	5.33E-04	1.86E-12

	S5087	6	3	3	1	20565333	20744926	179.594	501	7.44E-24	4.00E-02	4.00E-04	4.99E-27
	S5089	6	2	4	1	203167962	203362106	194.145	438	2.77E-09	2.67E-02	5.33E-04	1.86E-12
	S5090	6	3	3	1	208038505	208455658	417.154	892	7.44E-24	4.00E-02	4.00E-04	4.99E-27
	S5095	6	2	4	1	239925699	240061959	136.261	440	2.77E-09	2.67E-02	5.33E-04	1.86E-12
	S5104	6	2	4	3	29133845	29670933	537.089	1291	2.77E-09	2.67E-02	5.33E-04	1.86E-12
	S5108	6	2	4	3	186011964	186228961	216.998	259	2.77E-09	2.67E-02	5.33E-04	1.86E-12
	S5109	6	2	4	3	192442281	192621852	179.572	260	2.77E-09	2.67E-02	5.33E-04	1.86E-12
	S5110	6	2	4	3	194876514	195509651	633.138	1198	2.77E-09	2.67E-02	5.33E-04	1.86E-12
	S5114	6	2	4	4	24487818	24733937	246.12	403	2.77E-09	2.67E-02	5.33E-04	1.86E-12
	S5122	6	2	4	5	147296856	147460390	163.535	189	2.77E-09	2.67E-02	5.33E-04	1.86E-12
	S5126	6	3	3	6	37207819	37357639	149.821	209	7.44E-24	4.00E-02	4.00E-04	4.99E-27
	S5128	6	2	4	6	41527553	42320118	792.566	1519	2.77E-09	2.67E-02	5.33E-04	1.86E-12
	S5131	6	2	4	6	150147387	150278470	131.084	322	2.77E-09	2.67E-02	5.33E-04	1.86E-12
	S5133	6	2	4	6	155037515	155419151	381.637	754	2.77E-09	2.67E-02	5.33E-04	1.86E-12

	S5135	6	3	3	7	17116005	17969582	853.578	1743	7.44E-24	4.00E-02	4.00E-04	4.99E-27
	S5141	6	2	4	8	2353774	2510250	156.477	192	2.77E-09	2.67E-02	5.33E-04	1.86E-12
	S5142	6	2	4	8	5548502	6129625	581.124	2472	2.77E-09	2.67E-02	5.33E-04	1.86E-12
	S5143	6	2	4	8	20747941	21034004	286.064	658	2.77E-09	2.67E-02	5.33E-04	1.86E-12
	S5151	6	3	3	9	76695330	76829672	134.343	261	7.44E-24	4.00E-02	4.00E-04	4.99E-27
	S5152	6	2	4	9	89411806	89629605	217.8	538	2.77E-09	2.67E-02	5.33E-04	1.86E-12
	S5153	6	2	4	10	4567117	4918672	351.556	815	2.77E-09	2.67E-02	5.33E-04	1.86E-12
	S5156	6	2	4	10	6193032	7133489	940.458	2209	2.77E-09	2.67E-02	5.33E-04	1.86E-12
	S5158	6	3	3	10	10270906	10487471	216.566	283	7.44E-24	4.00E-02	4.00E-04	4.99E-27
	S5159	6	3	3	10	10635037	11241862	606.826	1129	7.44E-24	4.00E-02	4.00E-04	4.99E-27
	S5160	6	2	4	10	48972420	49404701	432.282	894	2.77E-09	2.67E-02	5.33E-04	1.86E-12
	S5165	6	2	4	10	120198681	120609072	410.392	701	2.77E-09	2.67E-02	5.33E-04	1.86E-12
	S5178	6	2	4	12	3742080	4349866	607.787	1159	2.77E-09	2.67E-02	5.33E-04	1.86E-12
	S5179	6	2	4	12	5296681	5611049	314.369	567	2.77E-09	2.67E-02	5.33E-04	1.86E-12

	S5180	6	2	4	12	52250686	52549360	298.675	731	2.77E-09	2.67E-02	5.33E-04	1.86E-12
	S5187	6	2	4	13	108842523	109533802	691.28	1574	2.77E-09	2.67E-02	5.33E-04	1.86E-12
	S5199	6	3	3	15	59266157	59502327	236.171	445	7.44E-24	4.00E-02	4.00E-04	4.99E-27
	S5200	6	4	2	15	70116822	70363632	246.811	470	9.56E-46	5.33E-02	2.66E-04	6.41E-49
	S5201	6	2	4	15	89916435	90692159	775.725	1371	2.77E-09	2.67E-02	5.33E-04	1.86E-12
	S5211	6	3	3	18	1787728	1916395	128.668	181	7.44E-24	4.00E-02	4.00E-04	4.99E-27
	S5212	6	3	3	18	2902404	3365773	463.37	970	7.44E-24	4.00E-02	4.00E-04	4.99E-27
	S5213	6	2	4	18	5334544	5559267	224.724	345	2.77E-09	2.67E-02	5.33E-04	1.86E-12
	S5216	6	3	3	18	73454806	73835678	380.873	886	7.44E-24	4.00E-02	4.00E-04	4.99E-27
	S5219	6	2	4	20	16073849	16744125	670.277	1575	2.77E-09	2.67E-02	5.33E-04	1.86E-12
	S5221	6	3	3	21	31365198	31772197	407	822	7.44E-24	4.00E-02	4.00E-04	4.99E-27
	S5227	5	2	3	1	60670085	61092333	422.249	953	5.52E-11	2.67E-02	4.00E-04	3.70E-14
	S5235	5	2	3	2	173630158	173740905	110.748	167	5.52E-11	2.67E-02	4.00E-04	3.70E-14
	S5243	5	3	2	3	173711383	174527931	816.549	1291	1.64E-28	4.00E-02	2.66E-04	1.10E-31

	S5244	5	4	1	3	182675426	182843221	167.796	275	7.78E-55	5.33E-02	1.33E-04	5.22E-58
	S5248	5	2	3	4	25861086	25967309	106.224	202	5.52E-11	2.67E-02	4.00E-04	3.70E-14
	S5261	5	2	3	5	170740401	171616668	876.268	1284	5.52E-11	2.67E-02	4.00E-04	3.70E-14
	S5262	5	2	3	6	12258537	12400414	141.878	276	5.52E-11	2.67E-02	4.00E-04	3.70E-14
	S5263	5	2	3	6	12530603	13356097	825.495	1230	5.52E-11	2.67E-02	4.00E-04	3.70E-14
	S5264	5	2	3	6	13467716	13961763	494.048	761	5.52E-11	2.67E-02	4.00E-04	3.70E-14
	S5265	5	2	3	6	13965926	14133342	167.417	294	5.52E-11	2.67E-02	4.00E-04	3.70E-14
	S5269	5	3	2	6	105198282	105396602	198.321	372	1.64E-28	4.00E-02	2.66E-04	1.10E-31
	S5270	5	2	3	6	105617855	105869500	251.646	451	5.52E-11	2.67E-02	4.00E-04	3.70E-14
	S5271	5	2	3	6	105880938	106358088	477.151	817	5.52E-11	2.67E-02	4.00E-04	3.70E-14
	S5287	5	2	3	8	21757132	22574864	817.733	1395	5.52E-11	2.67E-02	4.00E-04	3.70E-14
	S5297	5	2	3	8	141012783	141419373	406.591	761	5.52E-11	2.67E-02	4.00E-04	3.70E-14
	S5298	5	2	3	8	141528693	142423309	894.617	1876	5.52E-11	2.67E-02	4.00E-04	3.70E-14
	S5300	5	2	3	9	3086025	3570204	484.18	833	5.52E-11	2.67E-02	4.00E-04	3.70E-14

	S5307	5	2	3	10	52393841	52921401	527.561	854	5.52E-11	2.67E-02	4.00E-04	3.70E-14
	S5308	5	2	3	10	131566072	133466191	1900.12	3836	5.52E-11	2.67E-02	4.00E-04	3.70E-14
	S5310	5	2	3	11	116608867	117059966	451.1	1025	5.52E-11	2.67E-02	4.00E-04	3.70E-14
	S5314	5	3	2	12	12104304	12873451	769.148	1465	1.64E-28	4.00E-02	2.66E-04	1.10E-31
	S5316	5	3	2	12	117599701	117727676	127.976	313	1.64E-28	4.00E-02	2.66E-04	1.10E-31
	S5329	5	2	3	15	29253412	29454723	201.312	387	5.52E-11	2.67E-02	4.00E-04	3.70E-14
	S5330	5	3	2	15	88871064	89515313	644.25	1064	1.64E-28	4.00E-02	2.66E-04	1.10E-31
	S5331	5	2	3	15	92223230	92363732	140.503	280	5.52E-11	2.67E-02	4.00E-04	3.70E-14
	S5332	5	3	2	15	92454402	94026616	1572.215	3733	1.64E-28	4.00E-02	2.66E-04	1.10E-31
	S5337	5	2	3	17	1754605	2901672	1147.068	2088	5.52E-11	2.67E-02	4.00E-04	3.70E-14
	S5343	5	2	3	18	2362860	2601771	238.912	486	5.52E-11	2.67E-02	4.00E-04	3.70E-14
	S5344	5	2	3	18	71387001	71716158	329.158	842	5.52E-11	2.67E-02	4.00E-04	3.70E-14
	S5345	5	2	3	18	71872556	72350248	477.693	931	5.52E-11	2.67E-02	4.00E-04	3.70E-14
	S5350	5	3	2	21	31192067	31296802	104.736	126	1.64E-28	4.00E-02	2.66E-04	1.10E-31

	S5357	4	2	2	1	59765623	60409875	644.253	1126	1.59E-13	2.67E-02	2.66E-04	1.07E-16
	S5365	4	4	0	3	188777145	190116061	1338.917	2412	1.85E-68	5.33E-02	0.00E+00	1.24E-71
	S5366	4	2	2	3	194040829	194550048	509.22	1031	1.59E-13	2.67E-02	2.66E-04	1.07E-16
	S5376	4	2	2	5	148836339	149160025	323.687	574	1.59E-13	2.67E-02	2.66E-04	1.07E-16
	S5378	4	2	2	5	169417174	169904066	486.893	994	1.59E-13	2.67E-02	2.66E-04	1.07E-16
	S5379	4	2	2	5	170202868	170388480	185.613	368	1.59E-13	2.67E-02	2.66E-04	1.07E-16
	S5403	4	2	2	12	6806740	7895125	1088.386	1691	1.59E-13	2.67E-02	2.66E-04	1.07E-16
	S5404	4	2	2	12	127659470	128324588	665.119	1324	1.59E-13	2.67E-02	2.66E-04	1.07E-16
	S5405	4	2	2	12	129151166	129726850	575.685	1358	1.59E-13	2.67E-02	2.66E-04	1.07E-16
	S5413	4	2	2	14	100888741	101169312	280.572	611	1.59E-13	2.67E-02	2.66E-04	1.07E-16
	S5414	4	2	2	15	27219111	28712165	1493.055	1484	1.59E-13	2.67E-02	2.66E-04	1.07E-16
	S5437	4	2	2	21	27367234	27903745	536.512	1027	1.59E-13	2.67E-02	2.66E-04	1.07E-16
	S5438	4	2	2	21	41666784	41940272	273.489	800	1.59E-13	2.67E-02	2.66E-04	1.07E-16
	S5439	3	2	1	1	3731690	3850023	118.334	373	9.63E-18	2.67E-02	1.33E-04	6.46E-21

	S5445	3	2	1	3	73382679	73984585	601.907	1130	9.63E-18	2.67E-02	1.33E-04	6.46E-21
	S5454	3	2	1	8	4213245	4457827	244.583	1167	9.63E-18	2.67E-02	1.33E-04	6.46E-21
	S5456	3	2	1	9	135776385	136047043	270.659	503	9.63E-18	2.67E-02	1.33E-04	6.46E-21
	S5478	3	2	1	18	7460197	8787859	1327.663	2290	9.63E-18	2.67E-02	1.33E-04	6.46E-21
**SAS**	S432	7	3	4	8	66043202	66722849	679.648	184	9.21E-06	3.75E-01	2.02E-02	3.34E-08
	S541	6	3	3	7	55649184	57334218	1685.035	315	1.16E-06	3.75E-01	1.52E-02	4.22E-09
	S595	6	3	3	16	71247813	72152410	904.598	250	1.16E-06	3.75E-01	1.52E-02	4.22E-09
	S620	5	3	2	1	4467712	4644895	177.184	118	6.54E-08	3.75E-01	1.01E-02	2.37E-10
	S887	2	2	0	4	62816976	65403313	2586.338	791	1.68E-07	2.50E-01	0.00E+00	6.11E-10
	S892	2	2	0	8	128064327	129470564	1406.238	522	1.68E-07	2.50E-01	0.00E+00	6.11E-10

Legend: AAC = African Admixed, AFR = African, AJ = Ashkenazi Jewish, AMR = American Admixed, CAS = Central Asian, CHR = chromosome, EAS = East Asian, EUR = European, KB = kilobase, NSNP = number of single nucleotide polymorphisms, ROH = runs of homozygosity, SAS = South Asian, SNP = single nucleotide polymorphism. The Bonferroni threshold was adjusted by dividing 0.05 by the number of enriched ROHs to account for multiple comparisons.

Supplementary Table 21: *Runs of homozygosity pools enriched in individuals with consanguinity that pass Bonferroni correction intersecting with known Parkinson's disease, dystonia, and atypical parkinsonism gene regions and risk loci*

**Ancestry**	**ROH POOL**	**Total participants**	**Total cases**	**Total controls**	**CHR**	**Base position 1**	**Base position 2**	**ROH KB**	**NSNP**	**PD, parkinsonism, or dystonia gene**	**p-value**	**Proportion cases**	**Proportion controls**	**Bonferroni correction**
**AAC**	S109	6	2	4	15	50538139	51386390	848.252	2004	*USP8*	3.50E-12	3.33E-01	4.98E-03	1.95E-13
	S232	3	1	2	2	102642764	104499042	1856.279	3085	*MAP4K4*	1.27E-03	1.67E-01	2.49E-03	7.05E-05
	S318	3	1	2	16	17746297	18219727	473.431	870	*SYT17*	1.27E-03	1.67E-01	2.49E-03	7.05E-05
	S320	3	1	2	17	44919781	46788132	1868.352	2462	*UBTF FAM171A2 MAP3K14 CRHR1 MAPT-AS1 KANSL1 MAPT NSF WNT3*	1.27E-03	1.67E-01	2.49E-03	7.05E-05
	S393	2	1	1	6	32836793	35038899	2202.107	4929	*SLC44A4 HLA-DRB5 ¬†AHR*	6.18E-05	1.67E-01	1.24E-03	3.43E-06
	S404	2	1	1	7	22118464	24040285	1921.822	4492	*GPNMB KLHL7 NUPL2*	6.18E-05	1.67E-01	1.24E-03	3.43E-06
	S420	2	1	1	8	107635268	109528850	1893.583	4533	*SYBU*	6.18E-05	1.67E-01	1.24E-03	3.43E-06
	S457	2	1	1	12	131246290	133220283	1973.994	4851	*FBRSL1*	6.18E-05	1.67E-01	1.24E-03	3.43E-06

	S465	2	1	1	14	53431888	56631839	3199.952	7120	*GCH1*	6.18E-05	1.67E-01	1.24E-03	3.43E-06
AFR	S139	22	4	18	6	31415915	31521867	105.953	129	*SLC44A4*	7.62E-16	3.08E-01	1.10E-02	1.12E-17
	S1731	2	1	1	1	154314139	154678568	364.43	762	*PMVK GBAP1 ASH1L KRTCAP2*	1.10E-04	7.69E-02	6.13E-04	1.62E-06
	S1748	2	1	1	3	29269509	29926787	657.279	1803	*LINC00693*	1.10E-04	7.69E-02	6.13E-04	1.62E-06
	S1779	2	1	1	6	162191075	163885184	1694.11	4348	*PARK2*	1.10E-04	7.69E-02	6.13E-04	1.62E-06
	S213	17	3	14	3	52502292	52986528	484.237	999	*ITIH3 ITIH4*	7.45E-11	2.31E-01	8.58E-03	1.10E-12
	S41	50	4	46	6	28180332	28285755	105.424	192	*LOC100131289 RSL24D1P1*	4.80E-07	3.08E-01	2.82E-02	7.06E-09
	S433	11	2	9	6	33310149	33514091	203.943	296	*HLA-DRB5*	1.39E-06	1.54E-01	5.52E-03	2.05E-08
	S441	11	2	9	6	70720341	70955770	235.43	328	*RIMS1*	1.39E-06	1.54E-01	5.52E-03	2.05E-08
	S442	11	2	9	6	71029738	71360658	330.921	824	*RIMS1*	1.39E-06	1.54E-01	5.52E-03	2.05E-08
	S443	11	2	9	6	71437857	71584215	146.359	333	*RIMS1*	1.39E-06	1.54E-01	5.52E-03	2.05E-08
	S444	11	2	9	6	71739873	72255955	516.083	927	*RIMS1*	1.39E-06	1.54E-01	5.52E-03	2.05E-08
	S448	11	2	9	6	145205964	146492402	1286.439	2362	*RAB32*	1.39E-06	1.54E-01	5.52E-03	2.05E-08
	S458	11	2	9	8	108860914	109306803	445.89	1263	*SYBU*	1.39E-06	1.54E-01	5.52E-03	2.05E-08

	S461	11	2	9	9	34421281	34540917	119.637	152	*UBAP2*	1.39E-06	1.54E-01	5.52E-03	2.05E-08
	S475	11	2	9	15	50284963	50761937	476.975	1168	*USP8*	1.39E-06	1.54E-01	5.52E-03	2.05E-08
	S476	11	2	9	15	50763691	52095388	1331.698	2725	*USP8*	1.39E-06	1.54E-01	5.52E-03	2.05E-08
	S634	9	2	7	9	34768208	34889377	121.17	275	*UBAP2*	6.97E-08	1.54E-01	4.29E-03	1.02E-09
	S64	36	4	32	6	29403395	29516457	113.063	270	*TRIM40*	9.50E-10	3.08E-01	1.96E-02	1.40E-11
	S67	35	5	30	6	29636570	29764813	128.244	510	*TRIM40*	3.74E-16	3.85E-01	1.84E-02	5.50E-18
**AJ**	S1039	6	4	2	13	95543241	96945540	1402.3	1766	*MBNL2*	4.93E-05	8.16E-02	4.36E-04	4.41E-07
	S1106	5	4	1	1	204388918	205845567	1456.65	2230	*RAB29 SLC41A1*	4.46E-06	8.16E-02	2.18E-04	3.98E-08
	S151	12	7	5	12	40624560	40770009	145.45	277	*LRRK2 CNTN1 PDZRN4*	1.29E-07	1.43E-01	1.09E-03	1.15E-09
	S152	12	8	4	12	40788769	41954393	1165.625	2219	*LRRK2 CNTN1 PDZRN4 GXYLT1*	3.64E-10	1.63E-01	8.73E-04	3.25E-12
	S153	12	8	4	12	42529792	42653120	123.329	241	*GXYLT1*	3.64E-10	1.63E-01	8.73E-04	3.25E-12
	S1536	4	3	1	20	909079	3069655	2160.577	3718	*DDRGK1 PSMF1*	3.30E-04	6.12E-02	2.18E-04	2.95E-06
	S194	11	5	6	12	46228486	46901666	673.181	802	*SCAF11 SLC38A1*	3.91E-04	1.02E-01	1.31E-03	3.50E-06

	S24	27	9	18	12	33129292	34190817	1061.526	1700	*FGD4 PKP2 ALG10*	8.04E-05	1.84E-01	3.93E-03	7.18E-07
	S271	10	7	3	12	44649310	45574415	925.106	963	*SCAF11 SLC38A1*	2.21E-09	1.43E-01	6.54E-04	1.97E-11
	S272	10	6	4	12	45610378	45913265	302.888	231	*SCAF11 SLC38A1*	9.56E-07	1.22E-01	8.73E-04	8.54E-09
	S340	9	5	4	5	60276522	61263213	986.692	1263	*ELOVL7*	3.59E-05	1.02E-01	8.73E-04	3.21E-07
	S410	9	5	4	16	58499171	58934725	435.555	942	*CNOT1*	3.59E-05	1.02E-01	8.73E-04	3.21E-07
	S411	9	5	4	16	59418341	59854704	436.364	748	*CNOT1*	3.59E-05	1.02E-01	8.73E-04	3.21E-07
	S800	7	4	3	13	97321467	97927891	606.425	869	*MBNL2*	2.76E-04	8.16E-02	6.54E-04	2.46E-06
	S832	7	5	2	21	43194143	44092986	898.844	1041	*PDXK*	8.44E-07	1.02E-01	4.36E-04	7.53E-09
**AMR**	S200	3	3	0	7	70645942	73741147	3095.206	4119	*GALNT17*	1.91E-04	1.50E-01	0.00E+00	5.02E-06
	S211	3	3	0	10	15180551	15923173	742.623	1062	*ITGA8 FAM171A1*	1.91E-04	1.50E-01	0.00E+00	5.02E-06
**CAS**	S201	6	4	2	12	40662517	42677591	2015.075	3661	*LRRK2 CNTN1 PDZRN4 GXYLT1*	4.94E-09	2.67E-01	7.49E-03	7.27E-11
	S341	5	3	2	12	44863086	47069014	2205.929	2919	*SCAF11 SLC38A1*	7.06E-06	2.00E-01	7.49E-03	1.04E-07
	S353	5	3	2	17	14867684	17192330	2324.647	2715	*PIGL*	7.06E-06	2.00E-01	7.49E-03	1.04E-07

	S391	4	3	1	2	100722823	101159757	436.935	722	*MAP4K4*	2.86E-07	2.00E-01	3.75E-03	4.20E-09
	S398	4	3	1	2	165453151	167634399	2181.249	3154	*STK39*	2.86E-07	2.00E-01	3.75E-03	4.20E-09
	S399	4	3	1	2	167644704	167844661	199.958	360	*STK39*	2.86E-07	2.00E-01	3.75E-03	4.20E-09
	S457	4	4	0	6	110411111	111140162	729.052	1070	*FYN*	1.62E-13	2.67E-01	0.00E+00	2.39E-15
	S512	4	3	1	11	84667999	86999400	2331.402	3319	*DLG2*	2.86E-07	2.00E-01	3.75E-03	4.20E-09
	S608	3	2	1	3	151124172	153121159	1996.988	2899	*MED12L*	5.26E-04	1.33E-01	3.75E-03	7.74E-06
	S618	3	2	1	4	14928795	15879079	950.285	1654	*BST1 BST1 FAM200B CD38*	5.26E-04	1.33E-01	3.75E-03	7.74E-06
	S633	3	2	1	4	168662491	168844939	182.449	307	*CLCN3*	5.26E-04	1.33E-01	3.75E-03	7.74E-06
	S827	2	2	0	2	16368968	17996554	1627.587	2063	*KCNS3*	1.05E-05	1.33E-01	0.00E+00	1.54E-07
	S828	2	2	0	2	18171735	20375251	2203.517	3441	*KCNS3*	1.05E-05	1.33E-01	0.00E+00	1.54E-07
**EAS**	S1079	9	2	7	11	84504716	84644243	139.528	110	*DLG2*	1.04E-13	2.00E-01	3.04E-03	2.73E-15
	S1520	5	1	4	4	76422974	76561775	138.802	180	*SCARB2 FAM47E FAM47E-STBD1*	1.10E-03	1.00E-01	1.74E-03	2.90E-05
	S1594	5	1	4	12	46976766	47096091	119.326	236	*SCAF11*	1.10E-03	1.00E-01	1.74E-03	2.90E-05

	S1688	4	1	3	3	178535521	178850775	315.255	446	*AC076966.1*	2.32E-04	1.00E-01	1.30E-03	6.12E-06
	S1689	4	1	3	3	179312326	179604007	291.682	327	*AC076966.1*	2.32E-04	1.00E-01	1.30E-03	6.12E-06
	S1784	4	1	3	13	96654734	97783122	1128.389	937	*MBNL2*	2.32E-04	1.00E-01	1.30E-03	6.12E-06
	S1818	3	1	2	1	171080339	171247758	167.42	242	*VAMP4*	1.81E-05	1.00E-01	8.69E-04	4.75E-07
	S1824	3	1	2	2	18373851	20413320	2039.47	2497	*KCNS3*	1.81E-05	1.00E-01	8.69E-04	4.75E-07
	S1890	3	1	2	6	162333574	163245816	912.243	1217	*PARK2*	1.81E-05	1.00E-01	8.69E-04	4.75E-07
	S2068	2	1	1	21	36541167	36645075	103.909	121	*DYRK1A*	1.18E-07	1.00E-01	4.34E-04	3.10E-09
	S293	34	2	32	22	41017765	41949177	931.413	990	*RBX1*	3.68E-04	2.00E-01	1.39E-02	9.69E-06
	S427	22	2	20	6	145191755	146344288	1152.534	1464	*RAB32*	4.52E-06	2.00E-01	8.69E-03	1.19E-07
	S622	15	2	13	1	92380023	93057526	677.504	462	*MTF2*	1.47E-08	2.00E-01	5.65E-03	3.87E-10
	S654	15	2	13	11	82784557	82985843	201.287	221	*DLG2*	1.47E-08	2.00E-01	5.65E-03	3.87E-10
**EUR**	S1036	55	6	49	5	101986791	102198676	211.886	499	*PAM*	1.23E-11	8.00E-02	6.53E-03	9.50E-14
	S1057	54	6	48	5	102250985	102534752	283.768	580	*PAM*	7.30E-12	8.00E-02	6.40E-03	5.61E-14
	S1135	50	5	45	3	160436998	160601723	164.726	176	*SPTSSB*	9.37E-09	6.67E-02	6.00E-03	7.21E-11

	S1650	34	4	30	15	50918936	51207300	288.365	506	*USP8*	3.93E-08	5.33E-02	4.00E-03	3.02E-10
	S1699	33	3	30	12	123032370	123283558	251.189	232	*HIP1R*	1.28E-04	4.00E-02	4.00E-03	9.82E-07
	S1756	32	3	29	12	40435763	40587993	152.231	390	*LRRK2 CNTN1 PDZRN4*	9.31E-05	4.00E-02	3.86E-03	7.16E-07
	S1758	32	3	29	12	122319709	122626238	306.53	401	*HIP1R OGFOD2*	9.31E-05	4.00E-02	3.86E-03	7.16E-07
	S1831	31	4	27	12	40266815	40421822	155.008	387	*LRRK2 CNTN1*	6.38E-09	5.33E-02	3.60E-03	4.91E-11
	S1914	30	7	23	16	29991755	30108956	117.202	121	*SETD1A*	1.98E-30	9.33E-02	3.06E-03	1.52E-32
	S1984	29	4	25	12	41120483	41284161	163.679	295	*LRRK2 CNTN1 PDZRN4 GXYLT1*	1.54E-09	5.33E-02	3.33E-03	1.19E-11
	S2068	27	3	24	1	152969457	153087421	117.965	209	*HRNR*	1.37E-05	4.00E-02	3.20E-03	1.05E-07
	S2391	24	3	21	12	123582733	123773596	190.864	255	*HIP1R*	2.96E-06	4.00E-02	2.80E-03	2.28E-08
	S2439	23	5	18	3	152349004	152562959	213.956	343	*MED12L*	1.98E-19	6.67E-02	2.40E-03	1.52E-21
	S2468	23	4	19	6	71358447	71667230	308.784	582	*RIMS1*	5.04E-12	5.33E-02	2.53E-03	3.88E-14
	S2503	23	4	19	12	41615371	41820036	204.666	523	*PDZRN4 GXYLT1*	5.04E-12	5.33E-02	2.53E-03	3.88E-14
	S2534	22	4	18	1	155061442	155464151	402.71	432	*GBAP1 ASH1L PMVK KRTCAP2*	1.44E-12	5.33E-02	2.40E-03	1.10E-14

										GBA GBA1 SEMA4A				
	S2582	22	4	18	6	71744101	71857261	113.161	310	*RIMS1*	1.44E-12	5.33E-02	2.40E-03	1.10E-14
	S2770	20	3	17	2	74206825	74492875	286.051	245	*DCTN1*	1.91E-07	4.00E-02	2.27E-03	1.47E-09
	S2855	20	3	17	11	83322955	83767909	444.955	674	*DLG2*	1.91E-07	4.00E-02	2.27E-03	1.47E-09
	S2941	19	6	13	5	103842705	103979003	136.299	270	*PAM*	6.41E-35	8.00E-02	1.73E-03	4.93E-37
	S3282	16	3	13	1	153809535	154158721	349.187	478	*PMVK*	3.20E-09	4.00E-02	1.73E-03	2.46E-11
	S3283	16	3	13	1	154576516	154683482	106.967	179	*PMVK KRTCAP2 GBAP1 ASH1L PMVK*	3.20E-09	4.00E-02	1.73E-03	2.46E-11
	S3330	16	3	13	4	75905987	76402625	496.639	974	*SCARB2 FAM47E FAM47E-STBD1*	3.20E-09	4.00E-02	1.73E-03	2.46E-11
	S3389	16	5	11	12	31628444	31745841	117.398	202	*FGD4*	4.88E-28	6.67E-02	1.47E-03	3.75E-30
	S3470	15	5	10	3	178620339	178843865	223.527	499	*AC076966.1*	6.39E-30	6.67E-02	1.33E-03	4.91E-32
	S3522	15	4	11	9	33187899	33318590	130.692	178	*UBAP2*	2.10E-18	5.33E-02	1.47E-03	1.62E-20
	S3548	15	4	11	12	46545731	46736257	190.527	246	*SCAF11*	2.10E-18	5.33E-02	1.47E-03	1.62E-20
	S3565	15	4	11	15	45143019	45253280	110.262	160	*SPG11*	2.10E-18	5.33E-02	1.47E-03	1.62E-20

	S3567	15	9	6	16	49626115	49842961	216.847	421	*NOD2*	1.95E-105	1.20E-01	7.99E-04	1.50E-107
	S3568	15	9	6	16	49920928	50053087	132.16	221	*NOD2*	1.95E-105	1.20E-01	7.99E-04	1.50E-107
	S3569	15	3	12	16	74896352	75123124	226.773	403	*FA2H*	8.22E-10	4.00E-02	1.60E-03	6.33E-12
	S3627	14	2	12	4	2295975	2404772	108.798	288	*ADD1*	2.34E-04	2.67E-02	1.60E-03	1.80E-06
	S3635	14	4	10	4	111629692	112326279	696.588	839	*ANK2*	1.04E-19	5.33E-02	1.33E-03	7.97E-22
	S3663	14	5	9	6	162515945	163183260	667.316	1299	*PARK2*	4.51E-32	6.67E-02	1.20E-03	3.47E-34
	S3700	14	3	11	12	46848016	47082605	234.59	425	*SCAF11*	1.74E-10	4.00E-02	1.47E-03	1.34E-12
	S3726	14	2	12	17	62828828	63866085	1037.258	1077	*BRIP1*	2.34E-04	2.67E-02	1.60E-03	1.80E-06
	S3730	14	2	12	18	45670414	45809520	139.107	300	*SYT4*	2.34E-04	2.67E-02	1.60E-03	1.80E-06
	S3773	13	4	9	4	15021890	15336242	314.353	556	*BST1*	3.22E-21	5.33E-02	1.20E-03	2.48E-23
	S3776	13	3	10	4	77249630	77372145	122.516	304	*FAM47E-STBD1*	2.92E-11	4.00E-02	1.33E-03	2.24E-13
	S3808	13	4	9	7	23067749	23926540	858.792	1480	*GPNMB KLHL7 NUPL2*	3.22E-21	5.33E-02	1.20E-03	2.48E-23
	S3826	13	4	9	9	32939659	33090322	150.664	293	*UBAP2*	3.22E-21	5.33E-02	1.20E-03	2.48E-23
	S3880	13	2	11	14	75008572	75131153	122.582	109	*RPS6KL1*	1.20E-04	2.67E-02	1.47E-03	9.23E-07

	S3897	13	3	10	16	74092772	74280381	187.61	358	*FA2H*	2.92E-11	4.00E-02	1.33E-03	2.24E-13
	S3905	13	3	10	18	49658476	50557654	899.179	1740	*MEX3C*	2.92E-11	4.00E-02	1.33E-03	2.24E-13
	S3980	12	3	9	5	74945323	76297886	1352.564	2231	*SV2C*	3.64E-12	4.00E-02	1.20E-03	2.80E-14
	S3990	12	3	9	6	132608199	133163967	555.769	978	*RPS12* *HMGB1P13*	3.64E-12	4.00E-02	1.20E-03	2.80E-14
	S3991	12	4	8	7	24108825	24212310	103.486	213	*GPNMB*	5.64E-23	5.33E-02	1.07E-03	4.34E-25
	S4072	12	2	10	14	55104131	55449449	345.319	570	*GCH1*	5.54E-05	2.67E-02	1.33E-03	4.26E-07
	S4084	12	6	6	16	50982680	51286761	304.082	587	*NOD2*	1.35E-55	8.00E-02	7.99E-04	1.04E-57
	S4100	11	5	6	1	7698700	9019095	1320.396	2489	*DJ-1*	7.27E-41	6.67E-02	7.99E-04	5.59E-43
	S4115	11	3	8	1	160672214	160905887	233.674	426	*FCGR2A*	3.11E-13	4.00E-02	1.07E-03	2.40E-15
	S4116	11	3	8	1	160950381	161259053	308.673	581	*FCGR2A*	3.11E-13	4.00E-02	1.07E-03	2.40E-15
	S4154	11	2	9	3	17148999	17960084	811.086	857	*SATB1* *TBC1D5*	2.23E-05	2.67E-02	1.20E-03	1.71E-07
	S4208	11	4	7	6	147016387	147497968	481.582	474	*RAB32*	4.75E-25	5.33E-02	9.33E-04	3.65E-27
	S4211	11	3	8	6	161444200	161686497	242.298	542	*PARK2*	3.11E-13	4.00E-02	1.07E-03	2.40E-15
	S4252	11	3	8	8	129064176	129340981	276.806	463	*FAM49B*	3.11E-13	4.00E-02	1.07E-03	2.40E-15

	S4324	11	5	6	16	51998601	52452407	453.807	820	*CASC16 CHD9 TOX3 PHBP2*	7.27E-41	6.67E-02	7.99E-04	5.59E-43
	S4325	11	5	6	16	58288385	58461459	173.075	271	*CNOT1*	7.27E-41	6.67E-02	7.99E-04	5.59E-43
	S4361	10	3	7	1	162470743	162600585	129.843	222	*FCGR2A*	1.64E-14	4.00E-02	9.33E-04	1.26E-16
	S4395	10	3	7	3	123430486	123695355	264.87	309	*KPNA1*	1.64E-14	4.00E-02	9.33E-04	1.26E-16
	S4399	10	3	7	3	150713302	150872381	159.08	177	*MED12L*	1.64E-14	4.00E-02	9.33E-04	1.26E-16
	S4413	10	5	5	5	124279867	124566139	286.273	510	*ZNF608*	5.77E-45	6.67E-02	6.66E-04	4.44E-47
	S4414	10	4	6	5	125630120	125804876	174.757	396	*ZNF608*	1.54E-27	5.33E-02	7.99E-04	1.19E-29
	S4417	10	3	7	5	134230104	135130379	900.276	1001	*C5orf24 AC006077.1*	1.64E-14	4.00E-02	9.33E-04	1.26E-16
	S4434	10	3	7	7	22240125	22384820	144.696	284	*GPNMB*	1.64E-14	4.00E-02	9.33E-04	1.26E-16
	S444	129	6	123	14	66782901	67094191	311.291	340	*TMEM229B*	1.51E-04	8.00E-02	1.64E-02	1.16E-06
	S4479	10	2	8	10	119296873	119821778	524.906	965	*BAG3 INPP5F*	7.49E-06	2.67E-02	1.07E-03	5.76E-08
	S4520	10	2	8	14	37036574	37593915	557.342	589	*MIPOL1*	7.49E-06	2.67E-02	1.07E-03	5.76E-08
	S4538	10	4	6	16	53134146	53342939	208.794	264	*CASC16 CHD9 PHBP2*	1.54E-27	5.33E-02	7.99E-04	1.19E-29
	S4540	10	5	5	16	57412095	57855908	443.814	847	*CNOT1*	5.77E-45	6.67E-02	6.66E-04	4.44E-47

	S4549	10	2	8	18	51453244	51588653	135.41	199	*MEX3C SMAD4*	7.49E-06	2.67E-02	1.07E-03	5.76E-08
	S4632	9	4	5	5	124578128	125507383	929.256	1900	*ZNF608*	1.41E-30	5.33E-02	6.66E-04	1.09E-32
	S4731	9	3	6	16	19279092	19384618	105.527	226	*SYT17*	4.50E-16	4.00E-02	7.99E-04	3.46E-18
	S4733	9	4	5	16	52757088	52945399	188.312	335	*CASC16 CHD9 PHBP2*	1.41E-30	5.33E-02	6.66E-04	1.09E-32
	S4797	8	2	6	5	76506020	76662097	156.078	309	*SV2C*	3.81E-07	2.67E-02	7.99E-04	2.93E-09
	S4890	8	4	4	15	61048372	61374329	325.958	531	*VPS13C lincRNA*	2.26E-34	5.33E-02	5.33E-04	1.74E-36
	S4891	8	4	4	15	61540658	61835798	295.141	568	*VPS13C lincRNA VPS13C*	2.26E-34	5.33E-02	5.33E-04	1.74E-36
	S4892	8	5	3	15	62261912	62400579	138.668	302	*VPS13C*	3.12E-56	6.67E-02	4.00E-04	2.40E-58
	S4929	8	2	6	21	43836504	44092475	255.972	396	*PDXK*	3.81E-07	2.67E-02	7.99E-04	2.93E-09
	S4964	7	3	4	3	183467293	183579399	112.107	193	*MCCC1*	1.59E-20	4.00E-02	5.33E-04	1.23E-22
	S5009	7	2	5	8	23635862	23768832	132.971	298	*BIN3*	4.60E-08	2.67E-02	6.66E-04	3.54E-10
	S5087	6	3	3	1	20565333	20744926	179.594	501	*PINK1*	7.44E-24	4.00E-02	4.00E-04	5.72E-26
	S5104	6	2	4	3	29133845	29670933	537.089	1291	*LINC00693 RBMS3*	2.77E-09	2.67E-02	5.33E-04	2.13E-11
	S5165	6	2	4	10	120198681	120609072	410.392	701	*BAG3 INPP5F*	2.77E-09	2.67E-02	5.33E-04	2.13E-11

	S5187	6	2	4	13	108842523	109533802	691.28	1574	*RN7SKP10*	2.77E-09	2.67E-02	5.33E-04	2.13E-11
	S5244	5	4	1	3	182675426	182843221	167.796	275	*MCCC1*	7.78E-55	5.33E-02	1.33E-04	5.98E-57
	S5287	5	2	3	8	21757132	22574864	817.733	1395	*BIN3 BIN3*	5.52E-11	2.67E-02	4.00E-04	4.25E-13
	S5330	5	3	2	15	88871064	89515313	644.25	1064	*POLG1*	1.64E-28	4.00E-02	2.66E-04	1.26E-30
	S796	75	7	68	10	103044198	103193308	149.111	260	*GBF1*	1.47E-11	9.33E-02	9.06E-03	1.13E-13
	S881	64	6	58	1	92419450	93018831	599.382	859	*MTF2*	6.73E-10	8.00E-02	7.73E-03	5.17E-12
	S933	61	8	53	5	60612700	61170442	557.743	801	*ELOVL7*	3.33E-19	1.07E-01	7.06E-03	2.56E-21
	S953	59	5	54	3	161193918	161461944	268.027	332	*SPTSSB*	2.32E-07	6.67E-02	7.20E-03	1.79E-09
	S970	58	5	53	3	160982708	161090052	107.345	163	*SPTSSB*	1.71E-07	6.67E-02	7.06E-03	1.31E-09
**SAS**	S541	6	3	3	7	55649184	57334218	1685.035	315	*CHCHD2*	1.16E-06	3.75E-01	1.52E-02	3.88E-08
	S892	2	2	0	8	128064327	129470564	1406.238	522	*FAM49B*	1.68E-07	2.50E-01	0.00E+00	5.62E-09

Legend: AAC = African Admixed, AFR = African, AJ = Ashkenazi Jewish, AMR = American Admixed, CAS = Central Asian, CHR = chromosome, EAS = East Asian, EUR = European, KB = kilobase, NSNP = number of single nucleotide polymorphisms, ROH = runs of homozygosity, SAS = South Asian, SNP = single nucleotide polymorphism. The Bonferroni threshold was adjusted by dividing 0.05 by the number of enriched ROHs to account for multiple comparisons.

Supplementary Table 22: *Runs of homozygosity pools enriched in early-onset cases that pass Bonferroni correction*

Ancestry	ROH POOL	Total participants	Total cases	Total controls	CHR	Base position 1	Base position 2	ROH KB	NSNP	p-value	Proportion cases	Proportion controls	Bonferroni correction
AFR	S471	10	2	8	6	56159013	56285898	126.886	336	1.01E-04	0.095	4.90E-03	3.55E-07
	S483	10	2	8	8	50110122	50371754	261.633	619	1.01E-04	0.095	4.90E-03	3.55E-07
	S563	9	2	7	8	50929511	51047399	117.889	321	3.56E-05	0.095	4.29E-03	1.25E-07
	S590	9	2	7	13	83852625	84244091	391.467	829	3.56E-05	0.095	4.29E-03	1.25E-07
	S591	9	2	7	13	84346393	84466830	120.438	322	3.56E-05	0.095	4.29E-03	1.25E-07
	S740	7	2	5	2	21445013	22189250	744.238	1196	1.84E-06	0.095	3.07E-03	6.42E-09
	S741	7	2	5	2	22323717	22483764	160.048	331	1.84E-06	0.095	3.07E-03	6.42E-09
	S812	7	2	5	6	65338009	65627749	289.741	601	1.84E-06	0.095	3.07E-03	6.42E-09
	S861	7	2	5	13	82670299	82855364	185.066	478	1.84E-06	0.095	3.07E-03	6.42E-09
	S935	6	2	4	6	36751302	37009125	257.824	672	2.02E-07	0.095	2.45E-03	7.05E-10
	S960	6	2	4	8	65359477	65773338	413.862	785	2.02E-07	0.095	2.45E-03	7.05E-10
	S1000	6	2	4	11	97985598	98466352	480.755	1494	2.02E-07	0.095	2.45E-03	7.05E-10

	S1020	6	2	4	13	83291736	83567001	275.266	625	2.02E-07	0.095	2.45E-03	7.05E-10
	S1118	5	2	3	6	86802921	87341282	538.362	1289	9.30E-09	0.095	1.84E-03	3.25E-11
	S1157	5	2	3	8	96157210	97227476	1070.267	2080	9.30E-09	0.095	1.84E-03	3.25E-11
	S1376	4	2	2	11	95311792	95427262	115.471	215	9.44E-11	0.095	1.23E-03	3.30E-13
**EUR**	S4130	10	6	4	1	67749103	68044287	295.185	545	3.47E-05	0.006	5.33E-04	5.19E-08

Legend: AAC = African Admixed, AFR = African, CHR = chromosome, EUR = European, KB = kilobase, NSNP = number of single nucleotide polymorphisms, ROH = runs of homozygosity, SNP = single nucleotide polymorphism. The Bonferroni threshold was adjusted by dividing 0.05 by the number of enriched ROHs to account for multiple comparisons.

References

Foo, J.N. *et al.* (2020) 'Identification of Risk Loci for Parkinson Disease in Asians and Comparison of Risk Between Asians and Europeans: A Genome-Wide Association Study', *JAMA neurology*, 77(6), pp. 746–754.

Hop, P.J. *et al.* (2024) 'Systematic rare variant analyses identify RAB32 as a susceptibility gene for familial Parkinson's disease', *Nature genetics*, 56(7), pp. 1371–1376.

Kim, J.J. *et al.* (2024) 'Multi-ancestry genome-wide association meta-analysis of Parkinson's disease', *Nature genetics*, 56(1), pp. 27–36.

Loesch, D.P. *et al.* (2021) 'Characterizing the Genetic Architecture of Parkinson's Disease in Latinos', *Annals of neurology*, 90(3), pp. 353–365.

Magrinelli, F. *et al.* (2024) 'PSMF1 variants cause a phenotypic spectrum from early-onset Parkinson's disease to perinatal lethality by disrupting mitochondrial pathways', *medRxiv: the preprint server for health sciences* [Preprint]. Available at: https://doi.org/10.1101/2024.06.19.24308302.

Nalls, M.A. *et al.* (2019) 'Identification of novel risk loci, causal insights, and heritable risk for Parkinson's disease: a meta-analysis of genome-wide association studies', *Lancet neurology*, 18(12), pp. 1091–1102.

Rizig, M. *et al.* (2023) 'Identification of genetic risk loci and causal insights associated with Parkinson's disease in African and African admixed populations: a genome-wide association study', *Lancet neurology*, 22(11), pp. 1015–1025.

Simón-Sánchez, J. *et al.* (2012) 'Cooperative genome-wide analysis shows increased homozygosity in early onset Parkinson's disease', *PloS one*, 7(3), p. e28787.

Banner Authors: Global Parkinson's Genetics Program (GP2)

Country	Name	Institution	Funders and Disclosures
**Algeria**	Yasser Mecheri	Centre Hospitalo-Universitaire Dr Benbadis Constantine	Nothing to declare
**Argentina**	Emilia M Gatto	Sanatorio de la Trinidad Mitre- INEBA	Nothing to declare
	Marcelo Kauffman	Hospital JM Ramos Mejia	Nothing to declare
	Federico Capparelli	Centro de Educación Médica e Investigaciones Clínicas Norberto Quirno	Nothing to declare
**Armenia**	Samson Khachatryan	Somnus Neurology Clinic	Nothing to declare
	Zaruhi Tavadyan	Somnus Neurology Clinic	Nothing to declare
	Mariam Isayan	Somnus Neurology Clinic	Nothing to declare
**Australia**	Claire E Shepherd	Neuroscience Research Australia	The Sydney Brain Bank is located at and supported by Neuroscience Research Australia
	Kishore Kumar	Garvan Institute of Medical Research and Concord Repatriation General Hospital	Paul Ainsworth Family Foundation
	Melina Ellis	Concord Hospital	Nothing to declare
	Miguel E. Rentería	QIMR Berghofer Medical Research Institute	The Australian Parkinson's Genetics Study is supported by the Shake It Up Australia Foundation and The Michael J. Fox Foundation for Parkinson's Research
	Sulev Koks	Murdoch University	Nothing to declare
	Simon Rowe	Neuroscience Research Australia	Nothing to declare
	Laura Rudaks	Neuroscience Research Australia	Nothing to declare
	Dennis Yeow	Neuroscience Research Australia	Nothing to declare
	Carolyn Sue	Neuroscience Research Australia	Nothing to declare

	Victor Flores Ocampo	QIMR Berghofer Medical Research Institute	Nothing to declare
**Austria**	Alexander Zimprich	Medical University Vienna Austria	Nothing to declare
**Azerbaijan**	Kanan Jafarov	Istanbul Klinik	Nothing to declare
**Belgium**	David Crosiers	University of Antwerp	Nothing to declare
**Brazil**	Artur F. Schumacher-Schuh	Universidade Federal do Rio Grande do Sul / Hospital de Clínicas de Porto Alegre	Nothing to declare
	Carlos Rieder	Federal University of Health Sciences of Porto Alegre	Nothing to declare
	Paula Saffie Awad	Universidade Federal do Rio Grande do Sul	Global Parkinson's Genetics Program
	Vitor Tumas	University of São Paulo	Nothing to declare
	Sarah Camargos	Universidade Federal de Minas Gerais	Nothing to declare
	Lucas Faria Costa	Universidade Federal de Minas Gerais	Nothing to declare
**Canada**	Edward A. Fon	Montreal Neurological Institute	Nothing to declare
	Oury Monchi	Institut universitaire de gériatrie de Montréal	CIHR, Brain Canada, Parkinson Canada
	Edward Fon	McGill University	Nothing to declare
	Robert Thibault	Aligning Science Across Parkinson's	Nothing to declare
	Ziv Gan-Or	McGill University	Nothing to declare
	Anthony Lang	University of Toronto	Nothing to declare
	Konstantin Senkevich	McGill University	Nothing to declare
**Chile**	Benjamin Pizarro Galleguillos	Universidad de Chile	Nothing to declare
	Marcelo Miranda	Fundación Diagnosis	Nothing to declare
	Maria Leonor Bustamante	Faculty of Medicine Universidad de Chile	Nothing to declare
	Patricio Olguin	Universidad de Chile	Nothing to declare

	Pedro Chana	CETRAM	Nothing to declare
	Elias Fernandez	Hospital Regional de Concepción	Nothing to declare
**China**	Beisha Tang	Central South University	Nothing to declare
	Huifang Shang	West China Hospital Sichuan University	Nothing to declare
	Jifeng Guo	Xiangya Hospital	Nothing to declare
	Piu Chan	Capital Medical University	Nothing to declare
	Wei Luo	Zhejiang University	Nothing to declare
	Zhenhua Liu	Xiangya Hospital, Central South University	Nothing to declare
**Colombia**	Gonzalo Arboleda	Universidad Nacional de Colombia	Nothing to declare
	Jorge Orozco	Fundación Valle del Lili	Nothing to declare
	Marlene Jimenez del Rio	University of Antioquia	Nothing to declare
**Costa Rica**	Alvaro Hernandez	University of Costa Rica	Nothing to declare
**Denmark**	Per Borghammer	Aarhus University	Nothing to declare
**Egypt**	Mohamed Salama	The American University in Cairo	The AUC/ ASRT/ DAAD
	Walaa A. Kamel	Beni-Suef University	Nothing to declare
**Ethiopia**	Yared Z. Zewde	Addis Ababa University	Nothing to declare
**France**	Alexis Brice	Paris Brain Institute	Nothing to declare
	Jean-Christophe Corvol	Sorbonne Université	Nothing to declare
	Mari Vidailhet	Salpêtriere Hospital (AP-HP), Sorbonne Université	Nothing to declare
**Georgia**	Mariam Kekenadze	Tbilisi State Medical University	Nothing to declare
	Irine Khatiashvili	S. Khechinashvili University Hospital	Nothing to declare
**Germany**	Ana Westenberger	University of Lübeck	Nothing to declare

Anastasia Illarionova	Deutsches Zentrum für Neurodegenerative Erkrankungen	Nothing to declare
Brit Mollenhauer	University Medical Center Göttingen	Nothing to declare
Christine Klein	University of Lübeck	CK serves as a medical Advisor to Centogene on genetic testing reports in the field of movement disorders, except Parkinson's disease, and is a member of the Scientific Advisory Board of Retromer Therapeutics
Eva-Juliane Vollstedt	University of Lübeck	Nothing to declare
Franziska Hopfner	Department of Neurology, University Hospital, LMU Munich	Nothing to declare
Günter Höglinger	Department of Neurology, University Hospital, LMU Munich	Nothing to declare
Harutyun Madoev	University of Lübeck	Nothing to declare
Joanne Trinh	University of Lübeck	Nothing to declare
Katja Lohmann	University of Lübeck	Nothing to declare
Lara M. Lange	University of Lübeck and University Medical Center Schleswig-Holstein	Nothing to declare
Manu Sharma	University of Tubingen	Dr. Sharma is further funded by the Michael J Fox Foundation, USA Genetic Diversity in PD Program: GAP-India Grant ID: 009411.
Sergiu Groppa	University of Mainz	Nothing to declare
Thomas Gasser	University of Tübingen	Nothing to declare
Zih-Hua Fang	The German Center for Neurodegenerative Diseases	Nothing to declare
Karl Heilbron	Charité - Universitätsmedizin Berlin	Former employee of 23andMe, Inc. Owns stock and/or stock options in 23andMe, Inc.
Theresa Luth	University of Luebeck	Nothing to declare
Wenhua Sun	University of Teubingen	Nothing to declare

	Inke König	University of Lübeck	Nothing to declare
	Daniela Berg	University Medical Center Schleswig-Holstein	Nothing to declare
	Bernhard Haslinger	Technical University of Munich	Nothing to declare
	Teresa Kleinz	University of Lübeck	Nothing to declare
	Norbert Brüggemann	University of Lübeck	Dr. Brüggemann received honaria from Abbvie, Esteve, Ipsen, Merz, Teva and Zambon
**Ghana**	Albert Akpalu	University of Ghana Medical School	Nothing to declare
**Greece**	Georgia Xiromerisiou	University of Thessaly	Nothing to declare
	Georgios Hadjigorgiou	University of Thessaly	Nothing to declare
	Ioannis Dagklis	Aristotle University of Thessaloniki	Nothing to declare
	Ioannis Tarnanas	Ionian University	Nothing to declare
	Leonidas Stefanis	Biomedical research Foundation of the Academy of Athens	PPMI2 (funded by MJFF), ALAMEDA (H2020 grant), funding by HFRI
	Maria Stamelou	Diagnostic and Therapeutic Centre HYGEIA Hospital	The non-profit organisation for scientific research in Parkinson's disease and related disorders
	Efthymios Dadiotis	University of Thessaly	Nothing to declare
**Honduras**	Alex Medina	Hospital San Felipe	Nothing to declare
**Hong Kong**	Germaine Hiu-Fai Chan	Queen Elizabeth Hospital	Nothing to declare
	Nancy Ip	The Hong Kong University of Science and Technology	Nothing to declare
	Nelson Yuk-Fai Cheung	Queen Elizabeth Hospital	Nothing to declare
	Phillip Chan	The Hong Kong University of Science and Technology	Nothing to declare

	Xiaopu Zhou	The Hong Kong University of Science and Technology	Nothing to declare
India	Asha Kishore	Aster Medcity	Michael J. Fox Foundation
	Divya KP	Sree Chitra Tirunal Institute for Medical Sciences and Technology	Nothing to declare
	Pramod Pal	National Institute of Mental Health & Neurosciences	Nothing to declare
	Prashanth Lingappa Kukkle	Manipal Hospital	Nothing to declare
	Roopa Rajan	All India Institute of Medical Sciences	Nothing to declare
	Rupam Borgohain	Nizam's Institute Of Medical Sciences	Nothing to declare
**Iran**	Mehri Salari	Shahid Beheshti University of Medical Science	Nothing to declare
**Israel**	Tamara Shiner	Tel Aviv Sourasky Medical Center	Nothing to declare
	Avner Thaler	Tel Aviv Sourasky Medical Center	Nothing to declare
**Italy**	Andrea Quattrone	Magna Græcia University of Catanzaro	Nothing to declare
	Enza Maria Valente	University of Pavia	Nothing to declare
	Grazia Annesi	National Research Council	Nothing to declare
	Lucilla Parnetti	University of Perugia	Nothing to declare
	Micol Avenali	University of Pavia	Nothing to declare
	Monica Gagliardi	Magna Graecia University	Nothing to declare
	Tommaso Schirinzi	University of Rome Tor Vergata	Nothing to declare
	Caterina Galandra	IRCCS Mondino Foundation	Nothing to declare
	Anna De Rosa	University of Naples Federico II	Nothing to declare
**Japan**	Manabu Funayama	Juntendo University	Nothing to declare
	Nobutaka Hattori	Juntendo University faculty of medicine	Nothing to declare

	Tomotaka Shiraishi	Jikei University School of Medicine	Nothing to declare
**Kazakhstan**	Altynay Karimova	Institute of Neurology and Neurorehabilitation	Nothing to declare
	Gulnaz Kaishibayeva	Institute of Neurology and Neurorehabilitation	Nothing to declare
	Aigerim Utegenova	West Kazakhstan Marat Ospanov State Medical University	Nothing to declare
	Aigul Yemagambetova	West Kazakhstan Marat Ospanov State Medical University	Nothing to declare
	Vadim Akhmetzhanov	Medline medical center	Nothing to declare
	Seitzhan Aidarov	National Center for Neurosurgery	Nothing to declare
	Tautanova Raushan	Astana Medical University	Nothing to declare
	Bagyzhan Syzdykova Syzdykova	City multi-field hospital No. 1	Nothing to declare
	Dinara Alzhanova	Astana Medical University	Nothing to declare
	Zhanybek Myrzayev	International University of Postgraduate Education	Nothing to declare
	Saltanat Abdraimova	South Kazakhstan Medical Academy	Nothing to declare
**Kyrgyzstan**	Cholpon Shambetova	Kyrgyz State Medical Academy	Nothing to declare
**Luxembourg**	Rejko Krüger	University of Luxembourg	Nothing to declare
	Patrick May	University of Luxembourg	Nothing to declare
**Malaysia**	Ai Huey Tan	University of Malaya	Nothing to declare
	Azlina Ahmad-Annuar	University of Malaya	Nothing to declare
	Mohamed Ibrahim Norlinah	Universiti Kebangsaan Malaysia	Nothing to declare
	Nor Azian Abdul Murad	UKM Medical Molecular Biology Institute	Nothing to declare

	Shahrul Azmin	Universiti Kebangsaan Malaysia Medical Centre	Nothing to declare
	Shen-Yang Lim	University of Malaya	Nothing to declare
	Wael Mohamed	International Islamic University	Nothing to declare
	Yi Wen Tay	University of Malaya	Nothing to declare
	Azalea Tenerife Pajo	University of Malaya	Nothing to declare
**Mexico**	Daniel Martinez-Ramirez	Tecnologico de Monterrey	Nothing to declare
	Mayela Rodriguez-Violante	Instituto Nacional de Neurologia y Neurocirugia	Nothing to declare
	Paula Reyes-Pérez	Universidad Nacional Autónoma de México	Global Parkinson's Genetics Program
	Alejandra Medina Rivera	Universidad Nacional Autónoma de México	Nothing to declare
	Nancy Monroy Jaramillo	Instituto Nacional de Neurología y Neurocirugía	Nothing to declare
**Mongolia**	Bayasgalan Tserensodnom	Mongolian National University of Medical Sciences	Nothing to declare
**Nepal**	Rajeev Ojha	Tribhuvan University	Nothing to declare
**Netherlands**	Wilma Van De Berg	Vanderbilt University Medical Center	Nothing to declare
	Bas Bleom	Radboud University	Nothing to declare
	Bart Van de Warrenburg	Radboud University Medical Center	Nothing to declare
	Lisette Charbonnier	Brain Research and Innovation Center	Nothing to declare
**New Zealand**	Tim J. Anderson	University of Otago	Health Research Council of New Zealand; Ministry of Business Innovation and Employment (New Zealand), Neurological Foundation of New Zealand
	Toni L. Pitcher	University of Otago	Funding - Health Research Council of New Zealand

**Nigeria**	Arinola Sanyaolu	University of Lagos	Nothing to declare
	Njideka Okubadejo	University of Lagos	Michael J Fox Foundation; Tertiary Education Trust Fund (TETFUND) National Research Fund
	Oluwadamilola Ojo	College of Medicine of the University of Lagos	Nothing to declare
	Simon Izuchukwu Ozomma	University of Calabar Teaching Hospital	Nothing to declare
	Kolawole Wahab	University of Ilorin	Nothing to declare
**Norway**	Lasse Pihlstrøm	Oslo University Hospital	Southeastern Regional Health Authority, Norway and Michael J. Fox Foundation
	Manuela Tan	Oslo University Hospital	Southeastern Norway Regional Health Authority Norway and Michael J. Fox Foundation
	Ingeborg Haugesag Lie	Oslo University Hospital	Nothing to declare
	Jodi Maple-Grødem	Stavanger University Hospital	Nothing to declare
**Pakistan**	Shoaib Ur-Rehman	University of Science and Technology Bannu	Nothing to declare
**Peru**	Mario Cornejo-Olivas	Universidad Cientifica del Sur	Michael J. Fox Foundation for Parkinson's Research and Aligning Science Across Parkinson's Initiative
**Philippines**	Maria Leila Doquenia	Metropolitan Medical Center	Nothing to declare
	Raymond Rosales	Metropolitan Medical Center	Nothing to declare
**Puerto Rico**	Angel Vinuela	University of Puerto Rico	Nothing to declare
**Russia**	Elena Iakovenko	Research Center of Neurology	Nothing to declare
	Anna Gareeva	Ufa Federal Research Center	Nothing to declare
	Gulnara Akhmadeeva	Ufa Scientific Center	Nothing to declare

	Irina Gilyazova	Russian Academy of Sciences / Bashkir State Medical University	Nothing to declare
**Saudi Arabia**	Bashayer Al Mubarak	King Faisal Specialist Hospital and Research Center	Nothing to declare
	Muhammad Umair	King Abdullah International Medical Research Center	Nothing to declare
**Singapore**	Eng-King Tan	National Neuroscience Institute	Singapore National Medical Research Council (MOH-OFLCG-000207)
	Jia Nee Foo	Nanyang Technological University	Singapore National Medical Research Council (MOH-000559)
	Elaine Chew	Nanyang Technological University	Nothing to declare
**Slovenia**	Vesna van Midden	Ljubljana University Medical Centre	Nothing to declare
**South Africa**	Ferzana Amod	University of KwaZulu-Natal	Nothing to declare
	Jonathan Carr	University of Stellenbosch	Nothing to declare
	Soraya Bardien	Stellenbosch University	National Research Foundation of South Africa[Grant Number 129249]
	Nikita Pillay	University of the Western Cape	Nothing to declare
	Kathryn Step	Stellenbosch University	Nothing to declare
**South Korea**	Beomseok Jeon	Seoul National University Hospital	Nothing to declare
	Yun Joong Kim	Yongin Severance Hospital	Nothing to declare
	Jung Hwan Shin	Seoul National University	Nothing to declare
	Joowon Jang	Seoul National University	Nothing to declare
**Spain**	Esther Cubo	Hospital Universitario Burgos	Nothing to declare
	Ignacio Alvarez	University Hospital Mutua Terrassa	Nothing to declare

	Janet Hoenicka	Institut de Recerca Sant Joan de Deu	Fondo de Investigación Sanitaria, Instituto Salud Carlos III, Grant PI019/00126
	Katrin Beyer	Research Institute Germans Trias i Pujol	Nothing to declare
	Maria Teresa Periñan	Instituto de Biomedicina de Sevilla	Nothing to declare
	Pau Pastor	University Hospital Germans Trias i Pujol	Nothing to declare
	Ruben Fernandez-Santiago	Hospital Clínic de Barcelona	Nothing to declare
	Pilar Gómez Garre	Instituto de Biomedicina de Sevilla	Nothing to declare
	Pablo Mir	Instituto de Biomedicina de Sevilla	Nothing to declare
**Sudan**	Sarah El-Sadig	Faculty of medicine university of Khartoum	Nothing to declare
**Sweden**	Kajsa Brolin	Lund University	Nothing to declare
	Per Svenningsson	Karolinska Institute	Nothing to declare
	Maria Swanberg	Lund University	Nothing to declare
**Switzerland**	Christiane Zweier	Inselspital Bern, University of Bern	Nothing to declare
	Gerd Tinkhauser	University Hospital Bern	Nothing to declare
	Paul Krack	Inselspital Bern, University of Bern	Nothing to declare
**Taiwan**	Chin-Hsien Lin	National Taiwan University Hospital	Nothing to declare
	Hsiu-Chuan Wu	Chang Gung Memorial Hospital	Nothing to declare
	Pin-Jui Kung	National Taiwan University	Global Parkinson's Genetics Program
	Ruey-Meei Wu	National Taiwan University Hospital	Minister of Science and Technology, Taiwan Government; National Taiwan University; Parkinson foundation (USA); Michael J. Fox Foundation
	Yihru Wu	Chang Gung Memorial Hospital	Nothing to declare

**Tajikistan**	Ganieva Manizha	Avicenna Tajik State Medical University	Nothing to declare
**Tunisia**	Rim Amouri	National Institute Mongi Ben Hamida of Neurology	Nothing to declare
	Samia Ben Sassi	Mongi Ben Hmida National Institute of Neurology	Nothing to declare
**Turkey**	A. Nazlı Başak	Koç University	Kirac Foundation and Koc Univ.
	Gencer Genc	Şişli Etfal Training and Research Hospital	Nothing to declare
	Özgür Öztop Çakmak	Koç University	Nothing to declare
	Sibel Ertan	Koç University	Nothing to declare
**United Kingdom**	Alastair Noyce	Queen Mary University of London	Prof. Noyce reports grants from Parkinson's UK, Barts Charity, Cure Parkinson's, NIHR, Innovate UK, Virginia Keiley benefaction, Alchemab, Aligning Science Across Parkinson's and Michael J Fox Foundation. Consultancy and personal fees from Astra Zeneca, AbbVie, Profile, Roche, Biogen, UCB, Bial, Charco Neurotech, uMedeor and Britannia.
	Alejandro Martínez-Carrasco	University College London	Global Parkinson's Genetics Program
	Anette Schrag	University College London	NIHR UCL/H Biomedical Research Centre
	Anthony Schapira	University College London	ASAP
	Camille Carroll	University of Plymouth	C Carroll receives salary from University of Plymouth, University Hospitals Plymouth NHS Trust and National Institute of Health Research; she has received advisory, consulting, and/or lecture fees from AbbVie,

			Bial, Lundbeck, Global Kinetics, Britannia and Medscape, and research funding from Parkinson's UK, Edmond J Safra Foundation, National Institute of Health Research and Cure Parkinson’s
	Donald Grosset	University of Glasgow	Tracking Parkinson's (J-1101) is funded by Parkinson's UK
	Eleanor J. Stafford	University College London	Nothing to declare
	Henry Houlden	University College London	Nothing to declare
	Huw R Morris	University College London	Dr Morris is employed by UCL. In the last 12 months he reports paid consultancy from Roche and Amylyx ; lecture fees/honoraria - BMJ, Kyowa Kirin, Movement Disorders Society. Research Grants from Parkinson’s UK, Cure Parkinson’s Trust, PSP Association, CBD Solutions, Drake Foundation, Medical Research Council, Michael J Fox Foundation. Dr Morris is a co-applicant on a patent application related to C9ORF72 - Method for diagnosing a neurodegenerative disease (PCT/GB2012/052140)
	John Hardy	University College London	Nothing to declare
	Kin Ying Mok	Univeristy College London	Nothing to declare
	Mie Rizig	University College London	Nothing to declare
	Nicholas Wood	University College London	ASAP- CRN
	Nigel Williams	Cardiff University	Parkinson's UK

	Olaitan Okunoye	University College London	Nothing to declare
	Rauan Kaiyrzhanov	University College London	Nothing to declare
	Rimona Weil	University College London	Nothing to declare
	Seth Love	University of Bristol	Medical Research Council (UK) MR/T018569/1
	Simona Jasaityte	University College London	Nothing to declare
	Sumit Dey	Queen Mary University of London	The Michael J. Fox Foundation for Parkinson's Research
	Vida Obese	University College London	Nothing to declare
	Spencer Finch	Queen Mary University of London	Nothing to declare
	Valentina Escott-Price	Cardiff University	Nothing to declare
	Hamin Lee	St George's, University of London	Nothing to declare
	Roger Barker	University of Cambridge	Nothing to declare
	Mina Ryten	University College London	Nothing to declare
	Michele Hu	University of Oxford	Nothing to declare
	Laura Parkkinen	University of Oxford	Nothing to declare
	Kailash Bhatia	University College London	Nothing to declare
	Richard Walker	Northumbria Healthcare at NHS Foundation Trust	Nothing to declare
	Steve Gentleman	Imperial College London	Nothing to declare
	Thomas Warner	University College London	Nothing to declare
	David Burn	Newcastle University	Nothing to declare
	Christian Lambert	Imperial College London	Nothing to declare

	Caroline Williams-Gray	University of Cambridge	Nothing to declare
	Chris Morris	Newcastle University	Newcastle University, Alzheimer's Society, Alzheimer's Research UK, Medical Research Council
	Deborah Attuah	YLD	Nothing to declare
	Raquel Real	University College London	Nothing to declare
	Yen Tai	Imperial College London	Nothing to declare
**USA**	Alberto Espay	University of Cincinnati	Nothing to declare
	Alyssa O'Grady	The Michael J. Fox Foundation for Parkinson's Research	Nothing to declare
	Andrew B Singleton	National Institute on Aging	Michael J Fox Foundation for Parkinson's disease Research and Aligning Science Across Parkinson's Initiative
	Andrew K. Sobering	Augusta University / University of Georgia Medical Partnership	Nothing to declare
	Bernadette Siddiqi	The Michael J. Fox Foundation for Parkinson's Research	Nothing to declare
	Bradford Casey	The Michael J. Fox Foundation for Parkinson's Research	Nothing to declare
	Brian Fiske	The Michael J. Fox Foundation for Parkinson's Research	Nothing to declare
	Cabell Jonas	Mid-Atlantic Permanente Medical Group	Nothing to declare
	Carlos Cruchaga	Washington University	National Institutes of Health (R01AG044546 (CC), P01AG003991(CC, JCM), RF1AG053303 (CC), RF1AG058501 (CC), U01AG058922 (CC), RF1AG074007 (YJS)), the Chuck Zuckerberg Initiative (CZI), the Michael J. Fox Foundation (LI, CC), and the

			Department of Defense (LI-W81XWH2010849). The recruitment and clinical characterization of research participants at Washington University were supported by NIH P30AG066444 (JCM), P01AG03991(JCM), and P01AG026276(JCM).
	Caroline B. Pantazis	National Institutes of Health	Nothing to declare
	Charisse Comart	The Michael J. Fox Foundation for Parkinson's Research	Nothing to declare
	Claire Wegel	Indiana University	Nothing to declare
	Cornelis Blauwendraat	National Institutes of Health	Nothing to declare
	Dan Vitale	National Institutes of Health	Nothing to declare
	Deborah Hall	Rush University	Nothing to declare
	Dena Hernandez	National Institutes of Health	Nothing to declare
	Ejaz Shiamim	Kaiser Permanente	Nothing to declare
	Ekemini Riley	Coalition for Aligning Science	Nothing to declare
	Faraz Faghri	National Institutes of Health	F.F.'s participation in this research was supported in part by the Intramural Research Program of the NIH, National Institute on Aging (NIA), National Institutes of Health, Department of Health and Human Services; project number ZO1 AG000535, as well as the National Institute of Neurological Disorders and Stroke. F.F.'s participation in this project was part of a competitive contract awarded to Data

			Tecnica International LLC by the National Institutes of Health to support open science research.
	Geidy E. Serrano	Banner Sun Health Research Institute	Banner Sun Health Research Institute Brain and Body Donation Program of Sun City, Arizona for the provision of human biological materials. The Brain and Body Donation Program has been supported by the National Institute of Neurological Disorders and Stroke (U24 NS072026 National Brain and Tissue Resource for Parkinson's Disease and Related Disorders), the National Institute on Aging (P30 AG19610 and P30AG072980, Arizona Alzheimer's Disease Center), the Arizona Department of Health Services (contract 211002, Arizona Alzheimer's Research Center), the Arizona Biomedical Research Commission (contracts 4001, 0011, 05-901 and 1001 to the Arizona Parkinson's Disease Consortium) and the Michael J. Fox Foundation for Parkinson's Research
	Hampton Leonard	National Institute on Aging/National Institutes of Health	H.L.L is supported by a competitive contract awarded to Data Tecnica International LLC by the National

			Institutes of Health to support open science research
	Hirotaka Iwaki	Data Tecnica International	H.I is supported by a competitive contract awarded to Data Tecnica International LLC by the National Institutes of Health to support open science research
	Honglei Chen	Michigan State University	NIH/DoD/Parkinson Foundation/MSU Foundation/Gibby vs. Parky Foundation - No COI to disclose
	Ignacio F. Mata	Cleveland Clinic	Funding from MJFF and NIH
	Ignacio Juan Keller Sarmiento	Northwestern University	Nothing to declare
	Jared Williamson	Kaiser Permanente	Nothing to declare
	Jonggeol Jeff Kim	National Institutes of Health	Nothing to declare
	Joseph Jankovic	Baylor College of Medicine	Nothing to declare
	Joshua Shulman	Baylor College of Medicine / Texas Children's Hospital	Collection of samples and data at Baylor College of Medicine was supported by Huffington Foundation.
	Justin C. Solle	The Michael J. Fox Foundation for Parkinson's Research	Nothing to declare
	Kaileigh Murphy	The Michael J. Fox Foundation for Parkinson's Research	Nothing to declare
	Kamalini Ghosh Galvelis	Parkinson's Foundation	Nothing to declare
	Karen Nuytemans	University of Miami Miller School of Medicine	This work has been supported by the American Parkinson Disease Association and the Margaret Q. Landenberger Research Foundation.

	Karl Kieburtz	Beth Israel Deaconess Medical Center	Nothing to declare
	Kate Andersh	National Institute on Aging	Nothing to declare
	Katerina Markopoulou	North Shore University Health System	Nothing to declare
	Kenneth Marek	Institute for Neurodegenerative Disorders	Consultant for Michael J Fox Foundation, GE Healthcare, Roche, UCB, BIAL, Denali, Takeda, , Cerapsir, UCB, Biohaven, Neuron23, Aprinoia, Astellas, Calico, Genentech, Invicro
	Kristin S. Levine	Data Tecnica International	Nothing to declare
	Lana M. Chahine	University of Pittsburgh	Nothing to declare
	Laura Ibanez	Washington University	Nothing to declare
	Laurel Screven	National Institute on Aging	Nothing to declare
	Lauren Ruffrage	University of Alabama at Birmingham	Nothing to declare
	Lisa Shulman	University of Maryland	Nothing to declare
	Luca Marsili	University of Cincinnati	Nothing to declare
	Maggie Kuhl	The Michael J. Fox Foundation for Parkinson's Research	Nothing to declare
	Marissa Dean	University of Alabama at Birmingham	Dr. Dean is an investigator in studies funded by Abbvie, Inc., Hoffmann-La Roche, CHDI Foundation, Inc., Annexon, Inc., Retrophin, Inc, Neurocrine Biosciences, UniQure Biopharma B.V., Praxis Precision Medicines, Neuraly, Inc., Michael J. Fox Foundation for Parkinson’s Research, and US Army Medical Research and Material Command (grant#W81XWH-18-1-

			0508). In addition, Dr. Dean receives support through the Huntington's Disease Society of American Centers of Excellence program.
	Mary B Makarious	National Institutes of Health	Nothing to declare
	Matthew Farrer	University of Florida - Neurology	Nothing to declare
	Mathew Koretsky	National Institutes of Health	Nothing to declare
	Megan J. Puckelwartz	Northwestern University	Nothing to declare
	Miguel Inca-Martinez	Cleveland Clinic	Nothing to declare
	Mike A. Nalls	National Institutes of Health	M.A.N.'s participation in this project was part of a competitive contract awarded to Data Tecnica International LLC by the National Institutes of Health to support open science research. M.A.N. also currently serves as an advisor for Clover Therapeutics and Neuron23 Inc.
	Naomi Louie	The Michael J. Fox Foundation for Parkinson's Research	Nothing to declare
	Niccolò Emanuele Mencacci	Northwestern University	Nothing to declare
	Roger Albin	Universit of Michigan	P50NS123067; Parkinson's Foundation
	Roy Alcalay	Columbia University	Dr. Alcalay is funded by the Micharl J. Fox Foundation and the Parkinson's Foundation. He received consultation fees from Avrobio, Caraway, GSK, Merck, Sanofi, Ono Therapeutics and Takeda

	Ruth Walker	James J. Peters Veterans Affairs Medical Center	Research funding from the Department of Veterans Affairs (CSRD Merit Award CX002342), consulted for Teladoc, Inc. and received an honorarium from New York University (NYU) Medical Center.
	Sara Bandres-Ciga	National Institutes of Health	Nothing to declare
	Sohini Chowdhury	The Michael J. Fox Foundation for Parkinson's Research	Nothing to declare
	Sonya Dumanis	Aligning Science Across Parkinson's	Nothing to declare
	Steven Lubbe	Northwestern University	Nothing to declare
	Tao Xie	University of Chicago	Nothing to declare
	Tatiana Foroud	Indiana University School of Medicine	The Michael J. Fox Foundation for Parkinson's Research
	Thomas Beach	Sun Health Research Institution	Banner Sun Health Research Institute Brain and Body Donation Program of Sun City, Arizona for the provision of human biological materials. The Brain and Body Donation Program has been supported by the National Institute of Neurological Disorders and Stroke (U24NS072026 National Brain and Tissue Resource for Parkinson's Disease and Related Disorders), the National Institute on Aging (P30 AG19610 and P30AG072980, Arizona Alzheimer's Disease Center), the Arizona Department of Health Services (contract 211002, Arizona Alzheimer's

			Research Center), the Arizona Biomedical Research Commission (contracts 4001, 0011, 05-901 and 1001 to the Arizona Parkinson's Disease Consortium) and the Michael J. Fox Foundation for Parkinson’s Research
	Todd Sherer	The Michael J Fox Foundation for Parkinson's Research	Nothing to declare
	Yeajin Song	National Institutes of Health	Nothing to declare
	Dana Lewis	Aligning Science Across Parkinson's	Nothing to declare
	Shreya Menon	Gladstone Institutes	Nothing to declare
	Melissa Nirenberg	Icahn School of Medicine at Mount Sinai	Nothing to declare
	Spencer Grant	National Institutes of Health	Nothing to declare
	Shannon Ballard	National Institutes of Health	Nothing to declare
	Chad Shaw	Baylor College of Medicine	Nothing to declare
	Sidra Aslam	Banner Health	Nothing to declare
	Geidy Serrano	Banner Sun Health Research Institute	Nothing to declare
	Devin Sharp	Aligning Science Across Parkinson's	Nothing to declare
	Kensuke Daida	National Institute of Aging	Nothing to declare
	Rachel Saunders-Pullman	Icahn School of Medicine at Mount Sinai	Nothing to declare
	Michiko Kimura Bruno	The Queen's Medical Center	Nothing to declare
	Matt Farrer	University of Florida College of Medicine	Nothing to declare
	Haydeh Payami	The University of Alabama at Birmingham Heersink School of Medicine	Nothing to declare
	Ryan Pfingst	The Michael J Fox Foundation	Nothing to declare

	James B Leverenz	Cleveland Clinic	Nothing to declare
	Elizabeth Disbrow	LSU Health Shreveport	Nothing to declare
	Debi Brooks	The Michael J Fox Foundation	Nothing to declare
	Randy Schekman	University of California, Berkeley	Nothing to declare
	Un Kang	NYU Grossman School of Medicine	Nothing to declare
	Zbigniew K. Wszolek	Mayo Clinic College of Medicine	Nothing to declare
	Cyrus Zabetian	VA Puget Sound Health Care System	Nothing to declare
	Zach Chaney	The Michael J Fox Foundation	Nothing to declare
	Mark Cookson	National Institute of Health	Nothing to declare
	Christine Swanson-Fischer	National Institute of Health	Nothing to declare
	Conor Hennessey	The Michael J Fox Foundation	Nothing to declare
	Cassandra Barrett	The Michael J Fox Foundation	Nothing to declare
	Beate Ritz	University of California, Los Angeles	Nothing to declare
	Bradley Boeve	Mayo Clinic	Nothing to declare
	Ashley Rawls	University of Florida College of Medicine	Nothing to declare
**Vietnam**	Duan Nguyen	Hue University	Nothing to declare
	Toan Nguyen	Hue University	Nothing to declare
**Zambia**	Masharip Atadzhanov	University of Zambia	Nothing to declare
